# Probiotic Supplementation Prevents the Development of Ventilator-Associated Pneumonia for Mechanically Ventilated ICU Patients: A Systematic Review and Network Meta-analysis of Randomized Controlled Trials

**DOI:** 10.3389/fnut.2022.919156

**Published:** 2022-07-08

**Authors:** Cong Li, Fangjie Lu, Jing Chen, Jiawei Ma, Nana Xu

**Affiliations:** ^1^Department of Emergency Medicine, Affiliated Hospital of Xuzhou Medical University, Xuzhou, China; ^2^Laboratory of Morphology, Xuzhou Medical University, Xuzhou, China; ^3^Department of Critical Care Medicine, Changshu Hospital Affiliated to Nanjing University of Chinese Medicine, Changshu, China; ^4^Jiangsu Provincial Institute of Health Emergency, Xuzhou Medical University, Xuzhou, China; ^5^Department of Critical Care Medicine, The Affiliated Wuxi No. 2 People’s Hospital of Nanjing Medical University, Wuxi, China

**Keywords:** critical illness, probiotic, ventilator-associated pneumonia, mechanical ventilation, network meta-analysis

## Abstract

**Background:**

Ventilator-associated pneumonia (VAP) is one of the common critical complications of nosocomial infection (NI) in invasive mechanically ventilated intensive care unit (ICU) patients. The efficacy of total parenteral nutrition (TPN), enteral nutrition and/or adjuvant peripheral parenteral nutrition (EPN) supplemented with or without probiotic, prebiotic, and synbiotic therapies in preventing VAP among these patients has been questioned. We aimed to systematically and comprehensively summarize all available studies to generate the best evidence of VAP prevention for invasive mechanically ventilated ICU patients.

**Methods:**

Randomized controlled trials (RCTs) for the administration of TPN, EPN, probiotics-supplemented EPN, prebiotics-supplemented EPN, and synbiotics-supplemented EPN for VAP prevention in invasive mechanically ventilated ICU patients were systematically retrieved from four electronic databases. The incidence of VAP was the primary outcome and was determined by the random-effects model of a Bayesian framework. The secondary outcomes were NI, ICU and hospital mortality, ICU and hospital length of stay, and mechanical ventilation duration. The registration number of Prospero is CRD42020195773.

**Results:**

A total of 8339 patients from 31 RCTs were finally included in network meta-analysis. The primary outcome showed that probiotic-supplemented EPN had a higher correlation with the alleviation of VAP than EPN in critically invasive mechanically ventilated patients (odds ratio [OR] 0.75; 95% credible intervals [CrI] 0.58–0.95). Subgroup analyses showed that probiotic-supplemented EPN prevented VAP in trauma patients (OR 0.30; 95% CrI 0.13–0.83), mixed probiotic strain therapy was more effective in preventing VAP than EPN therapy (OR 0.55; 95% CrI 0.31–0.97), and low-dose probiotic therapy (less than 10^10^ CFU per day) was more associated with lowered incidence of VAP than EPN therapy (OR 0.16; 95% CrI 0.04–0.64). Secondary outcomes indicated that synbiotic-supplemented EPN therapy was more significantly related to decreased incidence of NI than EPN therapy (OR 0.34; 95% CrI 0.11–0.85). Prebiotic-supplemented EPN administration was the most effective in preventing diarrhea (OR 0.05; 95% CrI 0.00–0.71).

**Conclusion:**

Probiotic supplementation shows promise in reducing the incidence of VAP in critically invasive mechanically ventilated patients. Currently, low quality of evidence reduces strong clinical recommendations. Further high-quality RCTs are needed to conclusively prove these findings.

**Systamatic Review Registration:**

[https://www.crd.york.ac.uk/prospero/display_record.php?ID=CRD42020195773], identifier [CRD42020195773].

## Introduction

### Description of the Condition

Ventilator-associated pneumonia (VAP) is the most common fatal complication of nosocomial infection (NI) in intensive care unit (ICU) patients requiring invasive mechanical ventilation (MV) (1, 2). A high incidence of VAP is reported in critically ill patients with major traumatic brain injuries and lung contusion (3, 4), chronic obstructive pulmonary disease (5), and acute respiratory distress syndrome (6). Especially in critically ill patients undergoing extracorporeal membrane oxygenation during invasive MV, the incidence of VAP is as high as 35% (7). VAP is associated with increased duration of invasive MV, ICU length of stay (LOS) (1), hospital cost (8, 9), and risk of dying (10, 11).

The development of VAP involves dysbiosis and failure of host immune response (12). Gut dysbiosis is common in critically ill patients (13), especially in invasive mechanically ventilated patients (1) who are generally subjected to various types of stress, such as shock, trauma, and bleeding (14). These insults to the gut deteriorate beneficial commensal bacteria and promote the overgrowth of pathogens (15). In addition, antibiotic therapies exacerbate gut dysbiosis (16, 17). Beneficial commensal bacteria play critical roles in the maintenance of the intestinal barrier and host immunity (18, 19). Gut dysbiosis weakens the intestinal barrier, which comprises physical, secretory, and immunological barriers, by producting cytokines, mucins, and antimicrobial peptides through intestinal epithelial cells. These secretions in turn impair host immunity (12, 20). Hence, gut dysbiosis is believed to be involved in the occurrence of VAP in critically ill patients under invasive MV (12, 14).

### Recommendation of the Intervention

Microbes beneficial to hosts exert therapeutic effects on sites distant from habitats (21). Dietary interventions involving probiotic, prebiotic, and synbiotic supplementation can alleviate gut dysbiosis and enhance host immunity (22–24). Thus, in the European Union, probiotic and prebiotic supplements are recommended for critically ill patients to help maintain commensal microbiota (25). The American Society for Parenteral and Enteral Nutrition (A.S.P.E.N.) (26) and Canadian Clinical Practice Guidelines (CCPG) (19) recommend the use of probiotic and prebiotic supplementation to prevent dysbiosis in patients with critical illnesses. In addition, enteral nutrition and/or adjuvant peripheral parenteral nutrition (EPN) is more effective in protecting the intestinal barrier than total parenteral nutrition (TPN) (27, 28). The reducing effects of EPN on infectious morbidity as compared with those of TPN are well documented in meta-analyses involving a variety of populations with critical illnesses (29–33). Therefore, guidelines recommend the use of enteral nutrition for nutritional support therapy in critically ill patients and consider the use of supplemental parenteral nutrition (26, 30).

### Controversy of the Intervention

The use of these therapies to prevent VAP remains highly controversial. Previous randomized controlled trials (RCTs) (14, 34–37) and meta-analyses (38–42) based on high-quality RCTs suggested that probiotics, prebiotics, and synbiotics are associated with VAP prevention. Promising data seemed to support that probiotic or prebiotic supplementation is a strategy for preventing VAP. However, evidence needs to be considered when evaluating the benefits of probiotics for VAP prevention. Large multicenter RCTs (including 2653 critically ill patients requiring invasive MV) evaluated the effects of probiotics and showed no significant reduction in the incidence of VAP (43). In addition, probiotic or prebiotic supplements do not seem to be associated with a reduction in VAP in patients with trauma (44–46) or other critically illness (47–54). TPN did not exhibit lower efficacy than EPN in VAP treatment in patients in patients with shock (55), acute organophosphate poisoning (56), and other critically illnesses (57, 58). Our previous network meta-analysis (NMA) on NI in critically ill patients suggested that TPN and EPN supplemented with or without probiotic, prebiotic, and synbiotic therapies did not significantly prevent the incidence of VAP (59). Moreover, evidence has highlighted the higher risk of bacterial and fungal translocation resulting from synbiotic and probiotic therapies in critical patients with damaged intestinal mucosa and immunodeficiency (60–62). These patients even showed an increased risk of mortality (63). Thus, the guidelines only recommend probiotic therapy based on evidence-based medicine for surgical and medical patient populations (26).

### Importance of This Review

No study has compared the efficacy of TPN and EPN supplemented with or without probiotic, prebiotic, and synbiotic therapies in preventing VAP in critically ill patients under invasive MV. The traditional meta-analysis did not evaluate the risks and benefits of mixed treatments (64). Given that their efficacy in preventing VAP is uncertain, we conducted this systematic review and NMA to evaluate and rank probiotic-supplemented EPN, prebiotic-supplemented EPN, synbiotic-supplemented EPN, EPN, and TPN therapies in terms of their efficacy in preventing VAP in critically invasive mechanically ventilated patients. The effects of these therapies on NI, ICU LOS, duration of MV, mortality, and other clinically important outcomes were investigated. This study is intended to provide an evidence-based medical basis for exploring efficient and safe strategies for preventing or relieving VAP.

## Methods

### Approval

This study has been registered in Prospero (65), with number CRD42020195773.

### Eligibility Criteria for Considering Studies

#### Types of Studies

We included only full-text published RCTs in this NMA.

#### Types of Participants

Adult ICU patients (≥16 years) who underwent invasive MV.

#### Types of Interventions

We included studies comparing two or more of the five therapies (probiotic-supplemented EPN, prebiotic-supplemented EPN, synbiotic-supplemented EPN, EPN, and TPN). Probiotic therapies include mixed strains or a single strain, different routes of administration, and different dosage regimens.

#### Types of Outcome Measures

The primary outcome was the incidence of VAP. Secondary outcomes were incidence of NI, incidence of bloodstream infections (BSIs), incidence of urinary tract infection (UTI), incidence of diarrhea, hospital and ICU mortality, duration of MV, and hospital and ICU LOS.

### Exclusion Criteria

We excluded controlled clinical trials, quasi-RCTs, interrupted time series studies, controlled before and after studies, cluster-RCTs, and cross-over studies. Studies that used the same therapy in two study groups were excluded. Studies that were not published as full-text reports or did not report outcome variables or duplicate publications were also excluded.

### Search Strategy for Identifying Studies

#### Electronic Searches

We have systematically searched clinical trials from 2000 to 2021 in the electronic databases of Cochrane (CENTRAL), Embase, Pubmed, and Web of Science. Searching was not restricted by language. According to the relevant term combination proposed by Cochrane for RCT-related systematic reviews, a special search strategy was developed for each database (66).

We used the search MeSH terms “critically ill” OR “intensive care unit” OR “mechanical ventilation” AND “synbiotics” OR “probiotics” OR “prebiotics” OR “enteral nutrition” OR “parenteral nutrition” AND “ventilator-associated pneumonia” combined with RCTs for searching relevant literature. The search strategy is described in Supplementary File 1.

### Searching Other Resources

We searched Google Scholar to identify gray literature relevant to this topic and searched completed trials (latest search, 31 December 2021) in the following registers: ClinicalTrials.gov^1^; Chinese Clinical Trial Register^2^; World Health Organization (WHO) International Clinical Trials Registry Platform (ICTRP^3^); ISRCTN^4^; and Australian New Zealand Clinical Trials Registry^5^.

### Data Collection and Analysis

#### Study Selection

Three investigators independently selected studies according to the inclusion criteria. Any discrepancies among investigators were resolved through consensus and arbitration within the review team.

#### Definition of Interventions and Outcomes

The definitions of probiotics, prebiotics, and synbiotics were obtained from the expert consensus document of the International Scientific Association for Probiotics and Prebiotics (ISAPP). Synbiotic is a mixture comprising live microorganisms and substrate(s) selectively utilized by host microorganisms that confers a health benefit on hosts (67). Probiotics are live microorganisms that may confer health benefits on hosts, when administered in adequate amounts (68). By contrast, prebiotics are substrates that are selectively utilized by host microorganisms and confer a health benefit (23). All outcomes were based on the definitions used in the primary study. Hospital mortality was presumed when mortality had an unspecified location.

### Data Extraction

Three investigators independently extracted all the available data by using the Cochrane ARI Group’s data extraction form. The data included demographic information, intervention details, and data of primary and secondary outcomes. The following data were extracted from each study: author, published year, language, institution, funding, demographic information of participants (age range and gender), inclusion and exclusion criteria, methodological design (methods of blinding, allocation concealment, and randomization), intervention, treatment comparison (details of strains, dosage regimen, duration, and route of administration), and result (incidence of VAP and secondary outcomes). Discrepancies among investigators were resolved through consensus and arbitration within the review team. When necessary, we contacted the original authors to clarify unclear data and information on methodological quality.

### Risk of Bias Assessment

The methodological quality of each included study was evaluated according to the recommended approach in Cochrane reviews (69). The risk of bias (ROB) was evaluated in seven domains. Each domain was classified as high, unclear, or low and was adjudicated within each study. A study had low ROB when it had no high ROB domain or had only three or low unclear domains. A study was classified as moderate ROB when it had a high ROB domain or more than three unclear domains. All other studies were classified as high ROB. Three independent evaluators performed an assessment and reached a consensus on the results. The results were represented in a ROB table.

### Measures of Treatment Effect

#### Unit of Analysis Issues

The unit of analysis was each participant in a trial.

### Data Synthesis

Dichotomous outcomes were measured using the proportions of variables and estimated using odds ratio (OR). Continuous outcomes were measured using means and standard deviations and estimated using mean difference (MD). We expressed data as an OR or MD with 95% credible interval (CrI). A Bayesian random-effects model was conducted to assess the study effect sizes and synthesize evidence for overall outcomes. Dichotomous outcomes and continuous outcomes used binomial likelihood and normal likelihood, respectively. The first 20,000 iterations were annealing, and the subsequent 30,000 iterations were sampled. Continuous iterations were increased when the potential scale reduction factor was not close to 1.0 (70). We will conduct the surface under the cumulative ranking curve (SUCRA) for all outcomes to obtain a comprehensive ranking of each therapy (71).

### Assessment of Heterogeneity

The amount of heterogeneity variance of pairwise and network was calculated and conveyed using *I*^2^ statistic (72, 73). Heterogeneity was considered statistically significant when the *I*^2^ statistic was more than 50%, and the possible sources of heterogeneity for each outcome were discussed through subgroup analysis.

### Assessment of Inconsistency

Global and local methods, such as design-by-treatment tests and node splitting, were used in evaluating network consistency (74, 75). Inconsistency between direct and indirect comparison evidence was assumed when the *P*-value was less than 0.05.

### Assessment of Transitivity

The transitivity of network was assured by limiting the number of critically ill patients. The distributions of clinical variables, such as age and severity of illness at baseline, were used in investigating the transitivity assumption of NMA (71).

### Subgroup Analysis

Subgroup analyses including population, disease severity, dose, strains, the timing of initial nutrition, and quality, were used in assessing the impacts of key factors on the primary outcome and elucidating the source of possible heterogeneity. Specific populations, such as general ICU and trauma patients, were analyzed. In healthy people, the number of obligate anaerobes was around log^10^ colony-forming units (CFU)/g of feces (10^10^) on average (76). In some countries of the European Union and North America, the minimum recommended dose of probiotics was the minimum number of viable cells administered per day (10^9^ CFU) (77, 78). Therefore, according to the daily dose of enteral probiotics, we divided the subgroups into high-dose (probiotics of more than 10^10^ CFU per day) and low-dose (probiotics of less than 10^10^ CFU per day) groups. For the subgroup analysis of strains, we evaluated studies that used only *Lactobacillus rhamnosus* GG as probiotics and mixed strains as probiotics. Moreover, we divided the subgroups into high-severity of illness and low-severity illness groups according to the Acute Physiology And Chronic Health Evaluation (APACHE) II score of 20 at baseline or Simplified Acute Physiology Score (SPSA) II score of 35 at baseline (10, 79–81). According to the ROB results, the study was divided into high-quality (only low ROB) and low-quality (moderate and high ROB) groups. We divided the timing of initial nutrition according to Europe and American guidelines: within 24 h, within 48 h, and beyond 48 h (82, 83).

### Sensitivity Analysis

Sensitivity analysis was performed using the datasets of recruited centers (multicenter or single-center), quality of study (low and high quality), and diagnostic criteria of VAP (excluding studies with questionable diagnostic criteria). When the factors influencing the conclusions were identified, the potential causes of uncertainty were explored.

### Quality Assessment

We assessed the quality of evidence for each network estimate according to the Grading of Recommendations Assessment, Development, and Evaluation (GRADE) system (84). The following criteria were evaluated sequentially: study limitation, imprecision, inconsistency, indirectness, and publication bias. Study limitations were evaluated based on the assessment of ROB and the contribution matrix of network estimates. Imprecision was assessed according to the OR point estimate and confidence interval (CI). Inconsistency was assessed in accordance with heterogeneity and incoherence. Indirectness was evaluated in accordance with the transitivity of the network. Comparison-adjusted funnel plots were used in assessing publication bias (85, 86). The level of evidence was classified as high, moderate, low, or very low (84). The details of quality assessment are presented in Supplementary File 8.

### Statistical Software

R software (version 3.6.1) and Stata (version 14.0) were used for statistical analysis. The former was used (Netmeta 1.1–0 package, gemtc 0.8–2, and rjags 4–10 package) for Bayesian NMA, and the latter was used in drawing the comparison-adjusted funnel plots and network plots of the network. R software (ggplot2 3.2.1 package) was also used in drawing SUCRA graphics.

## Results

### Description of Included Studies

Characteristics related to each included study are presented in Tables 1, 2.

**TABLE 1:** Description of included studies.

ID	Author	Year	Country	Diseases	Design	N	Mean age (SD)	Male (%)	APACHE II Score	SAPS II Score	GCS Score	Intervention
1	Caparros et al. (47)	2001	Spain	MV Patients are expected to require enteral feeding for 7 days or longer	MC/SB	104	54.18 (25.65)	30	17.00 (6.01)	NR	NR	Prebiotics + EPN
						84	50.30 (24.06)		16.70 (7.54)	NR	NR	EPN
2	Kotzampassi et al. (109)	2006	Greece	Patients with severe multiple organ trauma predicted have a long ICU stay and need to be MV	MC/DB	35	52.90 (19.00)	80	19.36 (2.70)	NR	7.80 (4.24)	Synbiotics + EPN
						30	55.90 (18.00)	83	19.36 (2.10)	NR	7.64 (3.71)	Placebo + EPN
3	Radrizzani et al. (116)	2006	Italy	Patients are expected to require MV and nutrition for at least 4 days	MC/OP	142	51.50 (22.90)	71	NR	35.85 (13.48)	NR	EPN
						145	49.20 (26.00)	77	NR	35.95 (14.23)	NR	TPN
4	Spindler-Vesel et al. (108)	2006	United of kingdom	MV patients with severe multiple trauma and at least a 4-day ICU stay	SC/DB	26	48 (22.59)	78	13.5 (5.6)	NR	NR	Synbiotics + EPN
						29	36 (21.48)	NR	14 (5.2)	NR	NR	Prebiotics + ENP
						58	35 (20.8)	NR	12 (8.4)	NR	NR	EPN
5	Abdulmeguid and Hassan (117)	2007	Egypt	Patients requiring MV for >2 days after intensive care admission.	SC/OP	40	59	53	NR	35.78 (5.83)	NR	EPN
						40	58.3	63	NR	36.93 (5.83)	NR	TPN
6	Forestier et al. (48)	2008	France	MV patient with a ICU stay longer than 48 h	SC/DB	102	59.39 (14.57)	64	NR	44.60 (16.00)	NR	Probiotics + EPN
						106	56.13 (12.31)	76	NR	44.20 (15.30)	NR	Placebo + EPN
7	Giamarellos-Bourboulis et al. (37)	2009	Greece	Patients with severe multiple organ injuries necessitating emergency tracheal intubation and MV	MC/DB	36	52.90	NR	19.36	NR	7.64 (1.29)	Synbiotics + EPN
						36	55.90	NR	19.36	NR	7.80 (1.29)	EPN
8	Knight et al. (49)	2009	United of kingdom	Patients expected to require MV for a minimum of 48 h and with no contraindications to enteral nutrition	SC/DB	130	49.50 (19.60)	62	17.35 (8.25)	NR	NR	Synbiotics + EPN
						129	50.00 (18.50)	62	17.00 (7.50)	NR	NR	Placebo + EPN
9	Moses et al. (56)	2009	India	Patients with acute organophosphate poisoning needing MV support	SC/OP	29	29.41 (11.80)	76	NR	NR	NR	EPN
						30	30.83 (12.40)	73	NR	NR	NR	TPN
10	Barraud et al. (50)	2010	France	Patients under MV for a predicted period of at least 2 days	SC/DB	87	59.10 (15.90)	38	NR	58.60 (17.30)	NR	Probiotics + EPN
						80	61.80 (15.50)	44	NR	60.50 (19.60)	NR	Placebo + EPN
11	Frohmader et al. (118)	2010	Australia	MV patients are expected to require enteral feeding for at least 72 h	SC/DB	20	60.80 (15.60)	65	22.20 (8.90)	43.90 (15.00)	NR	Probiotics + EPN
						25	65.50 (9.80)	28	23.80 (10.20)	46.10 (19.40)	NR	Placebo + EPN
12	Morrow et al. (36)	2010	America	Patient would require MV for at least 72 h	SC/DB	73	67.50 (31.11)	59	22.70 (7.50)	NR	NR	Probiotics + EPN
						73	61.50 (26.67)	59	23.70 (8.00)	NR	NR	Placebo + EPN
13	Altintas et al. (58)	2011	Turkey	Patients need MV for at least 72 h	SC/OP	30	57.77 (19.88)	50	20.03 (7.43)	NR	NR	EPN
						41	57.95 (18.00)	56	22.66 (7.47)	NR	NR	TPN
14	Tan et al. (44)	2011	China	Patients with severe TBI need MV	SC/DB	16	NR	NR	NR	NR	NR	Probiotics + EPN
						19	NR	NR	NR	NR	NR	EPN
15	Aydoğmuş et al. (57)	2012	Turkey	Patients had received MV for at least 7 days	SC/OP	20	33.55 (14.14)	55	20.75 (4.74)	NR	8.40 (1.98)	EPN
						40	40.68 (17.64)	48	21.10 (5.85)	NR	7.35 (2.47)	TPN
16	Rongrungruang et al. (51)	2015	Thailand	Patients were expected to received MV for at least 72 h	SC/OP	75	68.95 (18.45)	60	19.88 (6.89)	NR	10.09 (3.70)	Probiotics + EPN
						75	73.09 (13.16)	57	19.41 (7.04)	NR	10.43 (4.05)	EPN
17	Malik et al. (119)	2016	Malaysia	Patients were expected to received MV in ICU	SC/DB	24	60.00 (14.40)	67	22.12 (6.00)	NR	NR	Probiotics + EPN
						25	55.00 (17.70)	68	23.00 (8.90)	NR	NR	Placebo + EPN
18	Zarinfar et al. (91)	2016	Iran	MV patients in ICU	SC/DB	30	41.18 (4.40)	70	NR	NR	NR	Probiotics + EPN
						30	48.18 (2.90)	10	NR	NR	NR	Placebo + EPN
19	Zeng et al. (52)	2016	China	Patients with an expected need of MV for at least 48 h	MC/OP	118	50.20 (18.20)	62	14.70 (3.90)	NR	NR	Probiotics + EPN
						117	54.60 (17.90)	56	16.60 (4.30)	NR	NR	EPN
20	Fazilaty et al. (45)	2018	Iran	Patients with severe multiple organ trauma need to received MV	SC/DB	20	41.56 (19.15)	90	61.73 (8.58)*	NR	6.28 (1.60)	Prebiotics + EPN
						20	33.62 (13.96)	90	59.84 (9.18)*	NR	6.00 (1.60)	Placebo + EPN
21	Kooshk et al. (35)	2018	Iran	MV patients for more than 48 h	SC/DB	30	54.37 (19.18)	40	22.70 (7.50)	NR	NR	Prebiotics + EPN
						30	59.53 (17.37)	63	23.70 (8.00)	NR	NR	EPN
22	Reignier et al. (55)	2018	French	Patients with shock expected to require more than 48 h of MV, concomitantly with vasoactive therapy	MC/OP	1202	66.00 (14.00)	67	NR	59.00 (19.00)	NR	EPN
						1208	66.00 (14.00)	67	NR	61.00 (20.00)	NR	TPN
23	Shimizu et al. (14)	2018	Japan	Patients were placed on a ventilator within 3 days after admission to the ICU, and who were diagnosed as having sepsis	SC/SB	35	73.29 (13.91)	71	19.00 (7.73)	NR	NR	Synbiotics + EPN
						37	72.93 (13.11)	59	20.00 (9.25)	NR	NR	EPN
24	Mahmoodpoor et al. (53)	2019	Iran	Patients had been undergoing mechanical ventilation for >48 h	MC/DB	48	59.10 (12.90)	54	24.10 (6.20)	NR	NR	Probiotics + EPN
						54	57.50 (14.50)	54	22.80 (4.70)	NR	NR	Placebo + EPN
25	Anandaraj et al. (54)	2019	India	Patient would require MV for at least 72 h	SC/DB	72	42.00 (17.00)	60	20.00 (8.00)	NR	NR	Probiotics + EPN
						74	43.00 (17.00)	57	19.00 (7.00)	NR	NR	Placebo + EPN
26	Jin et al. (97)	2019	China	Patients with severe stroke are expected to require MV	SC/OP	28	62.07 (10.94)	61	17.61 (3.56)	NR	7.71 (2.07)	Probiotics + EPN
						28	62.18 (11.12)	46	17.75 (3.71)	NR	8.11 (1.97)	EPN
27	Nseir et al. (120)	2019	France	Patients with shock who expected to require more than 48 h of MV, concomitantly with vasoactive therapy	MC/OP	78	65.40 (13.14)	76	14.8 (3.6)	57.80 (17.00)	NR	EPN
						73	65.45 (13.84)	75	14.3 (3.6)	57.29 (22.69)	NR	TPN
28	Habib et al. (46)	2020	Egypt	Patients with severe multiple trauma who expected to require more than 48 h of MV	SC/OP	32	39.08 (7.11)	75	NR	NR	9.06 (1.16)	Probiotics + EPN
						33	39.88 (7.91)	85	NR	NR	9.15 (1.21)	Placebo + EPN
29	Nazari et al. (34)	2020	Iran	Patients with severe multiple trauma submitted to MV for at least 48 h	SC/SB	73	52.18 (4.10)	67	NR	NR	6.22 (1.15)	Probiotics + EPN
						74	53.02 (3.99)	70	NR	NR	6.51 (1.10)	Placebo + EPN
30	Johnstone et al. (43)	2021	Canada	Critical patients expected to require MV more than 72 h	MC/DB	1318	60.10 (16.20)	59	22.30 (7.80)	NR	NR	Probiotics + EPN
						1332	59.60 (16.80)	61	21.70 (7.90)	NR	NR	Placebo + EPN
31	Tsilika et al. (92)	2021	Greece	Patients with severe multiple trauma who expected to require more than 10 days of MV	MC/DB	59	38.10 (17.20)	92	14.71 (5.26)	29.88 (8.99)	10.93 (3.42)	Probiotics + EPN
						53	43.80 (14.40)	76	15.40 (5.49)	31.30 (9.42)	9.87 (4.15)	Placebo + EPN

DB, double-blind; EPN, enteral nutrition and/or parenteral nutrition; GCS, Glasgow coma scale; MC, multicenter; MV, mechanical ventilation; NR, not reported; OP, open study; RCT, randomized controlled trials; SB, single-blind; SC, single-center; SD, mean difference; SAPS II, simplified acute physiology score II; TBI, traumatic brain injuries; TPN, total parenteral nutrition; *APACHE III.

**TABLE 2:** Description of included studies.

ID	Author	Intervention	Details of intervention	Dose or volume of intervention	Nutritional initiation	Duration of intervention	Drug administration
1	Caparros	Prebiotics + EPN	**Prebiotics:** fiber (8.9 g/L). **EPN:** Stresson Multifiber [75 g of proteins, with an 83:1 non-protein-calories-to-nitrogen ratio, and had a high content of arginine (11.8%), MCT (40%)]	**Prebiotics:** NR **EPN:** 25 kcal/kg per day.	<24 h	Until hospital discharge	Gastric or jejunal
		EPN	**EPN:** Nutrison Protein Plus (per liter, 62.5 g of proteins with a 102:1 non-protein calories-to-nitrogen ratio and was free of MCT and fiber)	**EPN:** 25 kcal/kg per day.			
2	Kotzampassi	Synbiotics + EPN	Synbiotics 2000 Forte **Probiotics:** *Pediococcus pentosaceus* 5–33:3, *Leuconostoc mesenteroides* 32–77:1, *L. paracasei* ssp 19, *L. plantarum* 2,362 **Prebiotics:** inulin, oat bran, pectin, resistant starch **EPN:** NR	**Probiotics:** 4 × 10^11^ CFUs QD **Prebiotics**:10 g QD **EPN:** NR	<24 h	15 days	Endoscopic gastrostomy or NG tube
		Placebo + EPN	**Placebo:** Maltodextrin **EPN:** NR	**Placebo:** Maltodextrin QD **EPN:** NR			
3	Radrizzani	EPN	**EN:** Perative (55% carbohydrate, 25% fat, 21% protein, 1.3 kcal/ml, containing per 100 ml: 0.8 g l-arginine, 0.15 g ω-3 fatty acids, 0.7 g ω-6 fatty acids, 2.9 mg vitamin E, 0.75 mg β-carotene, 2.2 mg zinc, and 7 μg selenium)	25–28 kcal/kg body weight per day	<24 h	NR	NR
		TPN	**TPN:** 59% carbohydrate, 23% fat, 18% protein, 1.2 kcal/ml				Central venous catheter
4	Spindler-Vesel	Synbiotics + EPN	Synbiotics 2000 **Probiotics:** Lactobacillus: *Pediococcus pentosaceus* 5–33:3, *Lactococcus raffinolactis* 32–77:1, *Lactobacillus paracasei* subsp *paracasei* 19, *Lactobacillus plantarum* 2362 **Prebiotics:** glucan, inulin, pectin, resistant starch **EPN:** Nutricomp standard (3.7 g protein, 13.7 g carbohydrate, 3.3 g fat per 100 mL)	**Probiotics:** 4 × 10^11^ CFUs QD **Prebiotics**:10 g QD **EPN:** 0.2 and 0.3 gN/kg body weight/d and an average of 25 non-protein kcal/kg body weight/d	<24 h	7 consecutive days. Until ICU discharge or EN discontinuation	NG tube feeding
		Prebiotics + EPN	**Prebiotics:** Nova Source (fermentable fibers) **EPN:** Nova Source (4.1 g protein, 14.4 g carbohydrate, 3.5 g fat, 2.2 g fermentable fibers as fermentable guar gum per 100 mL)	**Prebiotics:** NR **EPN:** 0.2 and 0.3 gN/kg body weight/d and an average of 25 non-protein kcal/kg body weight/d			
		EPN	**EPN:** Nutricomp peptide (4.5 g hydrolyzed protein, 16.8 g carbohydrate, 1.7 g fat per 100 mL), Alitraq (5.25 g protein, 16.5 g carbohydrate, 1.55 g fat and 1.55 g glutamine, 446 mg arginine, 154 mg ?-linolenic acid per 100 mL)	**EPN:** 0.2 and 0.3 gN/kg body weight/d and an average of 25 non-protein kcal/kg body weight/d			
5	Abdulmeguid	EPN	**EN:** standard feeding formula, which is a 1-calorie per mL	20–35 kcal/kg/day	<24 h	NR	NR
		TPN	**TPN:** NR				Central venous catheter
6	Forestier	Probiotics + EPN	**Probiotics:** *Lactobacillus casei* rhamnosus	**Probiotics**:1 × 10^9^ CFUs BID	<72 h	From the third day after admission to the ICU until discharge or death.	NG tube or oral after tube removal
		Placebo + EPN	**Placebo:** Growth medium without bacteria	**Placebo:** BID **EPN:** NR			
7	Giamarellos-Bourboulis	Synbiotics + EPN	Synbiotics 2000 Forte **Probiotics:** *Pediococcus pentosaceus* 5–33:3, *Leuconostoc mesenteroides* 32–77:1, *L. paracasei* ssp 19, *L. plantarum* 2,362 **Prebiotics:** inulin, oat bran, pectin, resistant starch **EPN:** Intestamin	**Probiotics:** 4 × 10^11^ CFUs QD **Prebiotics:** 10 g QD **EPN:** NR	<24 h	15 days	NG/gastrostomy tube
		EPN	**EPN:** Intestamin	**EPN:** NR			
8	Knight	Synbiotics + EPN	Synbiotics 2000 Forte^®^ **Probiotics:** *Pediococcus pentosaceus* 5–33:3, *Leuconostoc mesenteroides* 32–77:1, *L. paracasei* ssp 19, *L. plantarum* 2,362 **Prebiotics:** inulin, oat bran, pectin, resistant starch **EPN:** Nutrison Energy	**Probiotics:** 4 × 10^10^ CFUs BID **Prebiotics**:10 g BID **EPN:** NR	<24 h	A maximum of 28 days or ICU discharge or death	NG/OG tube
		Placebo + EPN	**Placebo:** Crystalline cellulose **EPN:** Nutrison Energy	**Placebo:** BID **EPN:** NR			
9	Moses	EPN	**EPN:** Hypocaloric EN	Maximum of 1000 cal/d and protein 28.32 g	<48 h	From the time of intubation to either the time of tracheostomy or extubation or transfer out of the medical ICU to the ward or death.	NG feeding
		TPN	**TPN:** Glucose and electrolyte	Maximum of 1000 cal/d and protein 28.32g			Central venous catheter
10	Barraud	Probiotics + EPN	**Probiotics:** Ergyphilus capsules (*Lactobacillus rhamnosus* GG, *Lactobacillus casei, Lactobacillus acidophilus, Bifidobacterium bifidum*) **EPN:** Fresubin^®^	**Probiotics:** 2 × 10^10^ CFUs QD **EPN:** 30–35 kcal/kg	<24 h	The entire period of mechanical ventilation but for a duration not exceeding 28 days	NG tube
		Placebo + EPN	**Placebo:** Excipient **EPN:** Fresubin^®^	**Placebo:** QD **EPN:** 30–35 kcal/kg			
11	Frohmader	Probiotics + EPN	**Probiotics:** VSL#3 (*Lactobacillus, Bifidobacterium, Streptococcus salivarius* subsp. *thermophilus*) **EPN:** Isosource or Renal or Diabetic Resource (Novartis, Melbourne, Australia)	**Probiotics:** 4.5 × 10^11^ CFUs BID **EPN:** 5 to 35 cal/kg per day and 0.8 to 1.5 g protein per kilogram per day.	<48 h	Until hospital discharge	NG/NJ tube
		Placebo + EPN	**Placebo:** Free of fiber and prebiotics additives **EPN:** Isosource or Renal or Diabetic Resource (Novartis, Melbourne, Australia)	**Placebo:** BID **EPN:** 25 to 35 cal/kg per day and 0.8 to 1.5 g protein per			
12	Morrow	Probiotics + EPN	**Probiotics:** *Lactobacillus rhamnosus* GG **EPN:** NR	**Probiotics:** 1 × 10^9^ CFUs BID **EPN:** NR	<48 h	Until extubation, tracheostomy placement, or death	Mixed with water Oropharynx and NG tube
		Placebo + EPN	**Placebo:** Inert inulin **EPN:** NR	**Placebo:** Inert inulin BID **EPN:** NR			
13	Altintas	EPN	**EN:** standard feeding formula, which is a 1-calorie per mL	25–30 kcal kg^–1^ d^–1^, Protein requirement was calculated as 1.2–1.5 g/kg/d (ideal body weight)	<48 h	NR	Gastric and postpyloric feeding
		TPN	**TPN:** 70% of the non-protein calories were met by carbohydrates and 30% by lipids.				Central or peripheral route
14	Tan	Probiotics + EPN	**Probiotics:** Golden Bifid: 0.5 × 10^8^ CFUs *Bifidobacterium longum*, 0.5 × 10^7^ CFUs *Lactobacillus bulgaricus*, 0.5 × 10^7^ CFUs *Streptococcus thermophilus* **EPN:** 3.8 g protein, 13.8 g carbohydrate, 3.4 g fat/100 ml, osmolarity 250 mOsm/l, no fibers	**Probiotics:**10^9^ CFUs per day **EPN:** 30 kcal/kg body weight/day and 0.2 gN/kg body weight/day	<48 h	21 days	NG tube
		EPN	**EPN:** 3.8 g protein, 13.8 g carbohydrate, 3.4 g fat/100 ml, osmolarity 250 mOsm/l, no fibers	**EPN:** 30 kcal/kg body weight/day and 0.2 gN/kg body weight/day			
15	Aydoğmuş	EPN	**EN:** first-line enteral nutrition (45% carbohydrate, 35% lipid and 20% protein) **PN:** NR	25–30 kcal kg^–1^ d^–1^	NR	NR	NG tube feeding
		TPN	**TPN:** a concentration of 1 mL/kcal that contained 20–30% dextrose, 20% lipid and 5.4–10% amino acid.				Central venous catheterization
16	Rongrungruang	Probiotics + EPN	**Probiotics:** *Lactobacillus casei* (Yakult) (Shirota strain) **EPN:**NR	**Probiotics:** 8 × 10^9^ CFUs for oral care after standard oral care QD. 8 × 10^9^ CFUs enteral feeding QD **EPN:** NR	NR	28 days or endotracheal tubes were removed	Feeding tube
		EPN	**EPN:** NR	**EPN:** NR			
17	Malik	Probiotics + EPN	**Probiotics:** *Lactobacillus acidophilus, Lactobacillus casei, Lactobacillus lactis, Bifidobacterium bifidum, Bifidobacterium longum, Bifidobacterium infantis* **EPN:** Osmolite 1 cal (standard formula), Glucerna (glucose intolerance formula), Peptamen (semielemental formula), and Novasource Renal (electrolyte and fluid restriction).	**Probiotics:**3 × 10^9^ CFUs BID **EPN:**25 kcal kg^–1^ d^–1^	<48 h	7 days	NG tube feeding
		Placebo + EPN	**Placebo:** Similar appearance and taste **EPN:** Osmolite 1 cal (standard formula), Glucerna (glucose intolerance formula), Peptamen (semielemental formula), and Novasource Renal (electrolyte and fluid restriction).	**Placebo:** 3g BID **EPN:**25 kcal kg^–1^ d^–1^			
18	ZarinfarN	Probiotics + EPN	**Probiotics:** *Lactobacillus* GG	TID	NR	NR	NG tube feeding
		Placebo + EPN	**Placebo:** NR	TID			
19	Zeng	Probiotics + EPN	**Probiotics:** Medilac-S: Bacillus subtilis 4.5 × 10^9^ CFUs/0.25 g and *Enterococcus faecalis* 0.5 × 10^9^ CFUs/0.25 g **EPN:** NR	**Probiotics:**0.5 g (1 × 10^10^ CFUs) TID **EPN:** NR	<24 h	Until tracheal extubation, discharge from the hospital or death, with a maximum duration of 14 days	NG tube feeding
		EPN	**EPN:** NR	**EPN:** NR			
20	Fazilaty	Prebiotics + EPN	**Prebiotics:** oat β-glucan **EPN:** high-protein enteral diet (20% protein, 30% lipid, and 50% carbohydrate)	**Prebiotics:** 3 g QD **EPN:** 25–30 kcal kg^–1^ d^–1^ based on weight and metabolic condition.	<48 h	NR	NG tube feeding
		Placebo + EPN	**Placebo:** maltodextrin **EPN:** high-protein enteral diet (20% protein, 30% lipid, and 50% carbohydrate)	**Prebiotics:** 3g QD **EPN:** 25–30 kcal kg^–1^ d^–1^ based on weight and metabolic condition.			
21	Kooshk	Prebiotics + EPN	**Prebiotics:** Fenugreek seed powder **EPN:** NR	**Prebiotics:**3 g BID **EPN:** NR	<24 h	NR	NG tube feeding
		EPN	**EPN:** NR	**EPN:** NR			
22	Reignier	EPN	**EN:** first-line enteral nutrition Isosmotic, isocaloric, normal-protein, polymeric preparations. **PN:** NR	Daily calorie target in kcal/kg of actual bodyweight was 20–25 during the first 7 days then 25–30 from day 8 to extubation.	<72 h	Until tracheal extubation or death	NG tube feeding
		TPN	**TPN:** NR				Central venous catheterization
23	Shimizu	Synbiotics + EPN	**Probiotics:** Yakult BL Seichoyaku (1 × 10^8^ CFUs/g B. breve strain/g and 1 × 10^8^ cfu/g *L. casei* strain Shirota) **Prebiotics:** galactooligosaccharides (Oligomate S-HP) **EPN:** standard polymeric diet Glucerna^®^-Ex 1 kcal/mL; 51:17:32 ratio of carbohydrate, protein, and fat; fiber 1.4 g/100 mL	**Probiotics:** 3 g (2 × 10^8^ CFUs) QD **Prebiotics:** 10 g QD **EPN:** 25–30 kcal/kg ideal body weight per day as the calorie goal.	<72 h	Until oral intake was initiated	Nasal tube
		EPN	**EPN:** standard polymeric diet Glucerna^®^-Ex 1 kcal/mL; 51:17:32 ratio of carbohydrate, protein, and fat; fiber 1.4 g/100 mL	**EPN:** 25–30 kcal/kg ideal body weight per day as the calorie goal.			
24	Mahmoodpoor	Probiotics + EPN	**Probiotics:** Lactocare: *Lactobacillus* species (casei, acidophilus, rhamnosus, bulgaricus), Bifidobacterium species (breve, longum), *Streptococcus thermophilus*. **EPN:** Standard formula (1 kcal/mL; Ensure)	**Probiotics:**10^10^ CFUs BID **EPN:**25 kcal/kg	<48 h	14 days or death	NG tube feeding
		Placebo + EPN	**Placebo:** Starch capsule **EPN:** Standard formula (1 kcal/mL; Ensure)	**Placebo:** BID **EPN:**25 kcal/kg			
25	Anandaraj	Probiotics + EPN	**Probiotics:** *Lactobacillus rhamnosus* **EPN:** NR	**Probiotics:** 2 × 10^9^ CFUs BID **EPN:** NR	NR	For a total of seven days or until extubation, whichever was earlier.	NG tube feeding
		Placebo + EPN	**Placebo:** Starch capsule **EPN:** NR	**Placebo:** BID **EPN:** NR			
26	Jin	Probiotics + EPN	**Probiotics:** Golden Bifid 0.5 g/tablet (0.5 × 10^7^ CFUs *Bifidobacterium longum*, 0.5 × 10^6^ CFUs *Lactobacillus bulgaricus*, 0.5 × 10^6^ CFUs *Streptococcus thermophilus*) **EPN:** TP-HE, TPF-T, TPF-D	**Probiotics:** 2 g (2.1 × 10^7^ CFUs) TID **EPN:** 25 kcal/kg ideal body weight per day as the calorie goal.	<48 h	NR	Nasal feeding
		EPN	**EPN:** TP-HE, TPF-T, TPF-D	**EPN:** 25 kcal/kg ideal body weight per day as the calorie goal.			
27	Nseir	EPN	**EN:** first-line enteral nutrition Isosmotic, isocaloric, normal-protein, polymeric preparations. **PN:** NR	Daily calorie target in kcal/kg of actual bodyweight was 20–25 during the first 7 days then 25–30 from day 8 to extubation.	<72 h	Until tracheal extubation or death	NG tube feeding
		TPN	**TPN:** NR				Central venous catheterization
28	Habib	Probiotics + EPN	**Probiotics:** Lacteol Forte^®^ *Lactobacillus* LB (*Lactobacillus delbrueckii* and *Lactobacillus fermentum*) **EPN:** NR	**Probiotics:**1 × 10^9^ CFUs TID **EPN:** NR	<24 h	NR	OG/NG tube feeding
		Placebo + EPN	**Placebo:** Starch capsule **EPN:** NR	**Placebo:** TID **EPN:** NR			
29	Nazari	Probiotics + EPN	**Probiotics:** Lactocare: *Lactobacillus* species (casei, acidophilus, rhamnosus, bulgaricus), Bifidobacterium species (breve, longum), *Streptococcus thermophilus*. **EPN:** NR	**Probiotics:** 1 × 10^10^ CFUs Q12h **EPN:** NR	NR	NR	NG tube feeding
		Placebo + EPN	**Placebo:** Starch capsule **EPN:** NR	**Placebo:** Q12h **EPN:** NR			
30	Johnstone	Probiotics + EPN	**Probiotics:** L rhamnosus GG **EPN:** NR	**Probiotics:**1 × 10^10^ CFUs BID **EPN:** NR	<24 h	Up to 60 days or until discharge from the ICU or until *Lactobacillus* species was isolated from a sterile site or cultured as the sole or predominant organism from a non-sterile site	Enteral feeding
		Placebo + EPN	**Placebo:** Microcrystalline cellulose **EPN:** NR	**Placebo:** BID **EPN:** NR			
31	Maria	Probiotics + EPN	**Probiotics:** *L. acidophilus* LA-5, *L. plantarum* UBLP-40, *B. animalis* subsp. *lactis* BB-12, *S. boulardii* Unique-28 **EPN:** NR	**Probiotics:** *L. acidophilus* LA-5 (1.75 × 10^9^ CFUs), *L. plantarum* UBLP-40 (0.5 × 10^9^ CFUs), *B. animalis* subsp. *lactis* BB-12 (1.75 × 10^9^ CFUs), *S. boulardii* Unique-28 (1.5 × 1010^9^ CFUs) BID **EPN:** NR	<24 h	15 days	Nasogastric or gastrostomy tube
		Placebo + EPN	**Placebo:** Powdered glucose polymer **EPN:** NR	**Placebo:** BID **EPN:** NR			

*CFUs, colony forming units; EN, enteral nutrition; EPN, enteral nutrition and/or adjuvant peripheral parenteral nutrition; MCT, medium-chain triglycerides; NG, nasogastric; NR, not reported; OG, orogastric; PN, parenteral nutrition; TPN, total parenteral nutrition; TBI, traumatic brain injuries; TPN, total parenteral nutrition.*

### Results of the Search

**FIGURE 1:**
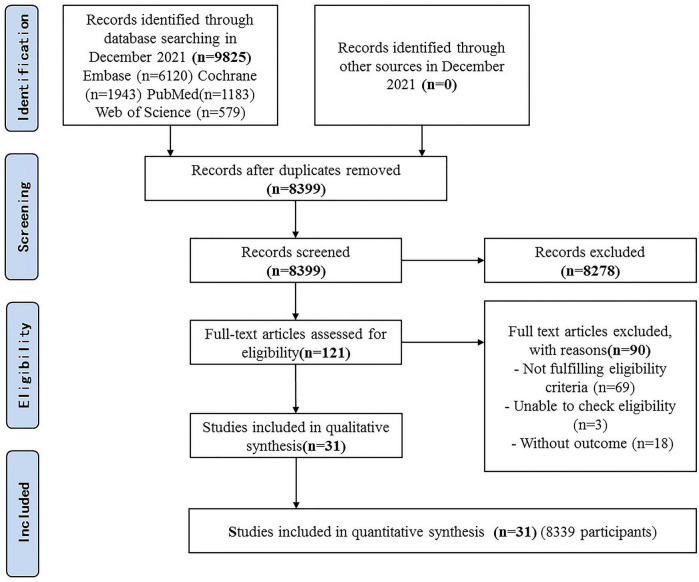
Flow diagram of included studies.

A total of 9825 articles were identified, of which 121 articles were potentially eligible articles. Overall, 31 RCTs were included after full texts were retrieved (Figure 1).

### Included Studies

Thirty-one studies were published between 2001 and 2021 in 16 countries and comprised 8339 patients. Among them, 29 articles were published in English, and one article each was published in Chinese and Arabic. Fourteen trials (45%) recruited patients from Asia; fourteen trials (45%), from Europe; two (6%) trials, from Americas; and one (4%) trial, from Oceania. Sample size ranged from 33 to 2650, with a mean of 132 participants (SD = 300). The mean age was 53 years (SD = 11). In total, 5167 (62%) males were included in the sample population. One study (4%) randomly assigned participants to three groups. Ten studies (32%) were multicenter studies, seventeen (55%) were double-blind studies, and eleven (35%) were open-label studies. The most included diseases were general diseases in the ICU, followed by sepsis or septic shock, severe multiple trauma, severe stroke, brain trauma, and poisoning. The mean APACHE II score was 21 (SD = 9), the mean SAPS II score was 46 (SD = 11), and the mean GCS score was 8 (SD = 1). The details were presented in Table 1.

Sixteen trials compared probiotics-supplemented EPN with EPN, seven trials compared EPN with TPN, five trials compared synbiotics-supplemented EPN with EPN, four trials comparing prebiotics-supplemented EPN with EPN, and one trial compared synbiotics-supplemented EPN with prebiotics-supplemented EPN. No trial compared synbiotics-supplemented EPN with probiotics-supplemented EPN, synbiotics-supplemented EPN with TPN, probiotics-supplemented EPN with TPN, probiotics-supplemented EPN with prebiotics-supplemented EPN, and prebiotics-supplemented EPN with TPN. The dose of probiotics varied from 6 × 10^7^ CFU per day to 9 × 10^11^ CFU per day. Participants in the majority of trials received initial nutritional support therapy within 72 h. The details were presented in Table 2.

A total of 25 studies reported VAP, 12 reported NI, 9 reported BSIs; 12 reported UTI, and 15 reported diarrhea. Fourteen reported ICU mortality, eighteen reported hospital mortality, twenty-five reported ICU LOS, seventeen reported hospital mortality, and eighteen reported duration of MV. Data for all outcomes was presented in Table 3.

**TABLE 3:** Reported clinical outcomes of included studies.

ID	Intervention	VAP (n/N)	Nl (n/N)	BSIs (n/N)	UTI (n/N)	Diarrhea (n/N)	Hospital Mortality (n/N)	ICU Mortality (n/N)	Hospital LOS Mean (SD) (day)	ICU LOS Mean (SD) (day)	MV LOS Mean (SD) (day)
1	Prebiotics + EPN	50/104	76/104	14/104	14/104	NR	21/104	16/104	38.28 (31.57)	18.58 (11.65)	12.35 (8.27)
	EPN	22/84	47/84	9/84	4/84	NR	26/84	18/84	31.82 (16.85)	15.06 (8.30)	10.53 (6.41)
2	Synbiotics + EPN	NR	22/35	NR	6/35	5/35	5/35	5/35	NR	27.70 (15.20)	16.70 (9.50)
	Placebo + EPN	NR	27/30	NR	13/30	10/30	9/30	9/30	NR	41.30 (20.50)	29.70 (16.50)
3	EPN	NR	7/142	1/142	0/142	NR	17/142	NR	32.20 (28.66)	17.60 (16.98)	NR
	TPN	NR	19/145	2/145	1/145	NR	20/145	NR	36.80 (28.66)	21.60 (16.98)	NR
4	Synbiotics + EPN	NR	5/26	0/26	0/26	NR	NR	2/26	NR	14.07 (10.04)	12.17 (8.86)
	Prebiotics + EPN	NR	17/29	2/29	0/29	NR	NR	2/29	NR	15.64 (8.58)	11.64 (5.46)
	EPN	NR	29/58	2/58	1/58	NR	NR	3/58	NR	14.01 (11.20)	9.97 (8.13)
5	EPN	NR	14/40	NR	NR	NR	7/40	NR	10.82 (3.30)	7.60 (4.21)	6.25 (4.07)
	TPN	NR	20/40	NR	NR	NR	11/40	NR	12.95 (3.30)	10.32 (4.21)	8.65 (4.07)
6	Probiotics + EPN	24/102	NR	NR	NR	NR	NR	NR	NR	NR	NR
	Placebo + EPN	24/106	NR	NR	NR	NR	NR	NR	NR	NR	NR
7	Synbiotics + EPN	15/36	NR	5/36	6/36	NR	5/36	NR	NR	NR	NR
	EPN	16/36	NR	13/36	11/36	NR	10/36	NR	NR	NR	NR
8	Synbiotics + EPN	12/130	NR	NR	NR	7/130	35/130	28/130	21.11 (20.99)	6.70 (6.00)	5.35 (5.25)
	Placebo + EPN	17/129	NR	NR	NR	9/129	42/129	34/129	19.05 (18.74)	8.05 (8.25)	6.41 (6.00)
9	EPN	12/29	14/29	NR	2/29	0/29	NR	3/29	14.82 (8.19)	9.79 (5.46)	10.39 (6.63)
	TPN	10/30	15/30	NR	5/30	1/30	NR	3/30	11.47 (5.84)	8.53 (5.84)	8.57 (6.23)
10	Probiotics + EPN	23/87	30/87	NR	4/87	48/87	NR	21/87	26.60 (22.30)	18.70 (12.40)	NR
	Placebo + EPN	15/80	30/80	NR	4/80	42/80	NR	21/80	28.90 (26.40)	20.20 (20.80)	NR
11	Probiotics + EPN	NR	NR	NR	NR	NR	5/20	NR	NR	5.97 (5.30)	6.00 (5.20)
	Placebo + EPN	NR	NR	NR	NR	NR	3/25	NR	NR	5.54 (4.07)	6.71 (5.25)
12	Probiotics + EPN	17/73	NR	NR	NR	46/73	12/73	NR	21.40 (14.90)	14.80 (11.80)	9.50 (6.30)
	Placebo + EPN	33/73	NR	NR	NR	57/73	15/73	NR	21.70 (17.40)	14.60 (11.60)	9.60 (7.20)
13	EPN	5/30	NR	NR	NR	2/30	13/30	8/30	32.98 (30.16)	15.36 (10.12)	7.00 (3.50)
	TPN	11/41	NR	NR	NR	0/41	20/41	18/41	31.19 (22.28)	17.19 (13.06)	9.18 (6.53)
14	Probiotics + EPN	7/16	7/16	0/16	0/16	NR	NR	NR	NR	NR	NR
	EPN	13/19	14/19	0/19	1/19	NR	NR	NR	NR	NR	NR
15	EPN	9/20	NR	NR	NR	NR	NR	NR	NR	NR	NR
	TPN	17/40	NR	NR	NR	NR	NR	NR	NR	NR	NR
16	Probiotics + EPN	18/75	NR	NR	NR	19/75	NR	NR	24.61 (21.71)	33.28 (19.62)	NR
	EPN	22/75	NR	NR	NR	14/75	NR	NR	28.22 (35.07)	18.80 (5.22)	NR
17	Probiotics + EPN	NR	NR	NR	NR	NR	NR	NR	NR	10.90 (3.90)	8.40 (3.50)
	Placebo + EPN	NR	NR	NR	NR	NR	NR	NR	NR	15.80 (7.80)	14.00 (8.00)
18	Probiotics + EPN	7/30	NR	NR	NR	1/30	5/30	NR	24.10 (5.60)	14.20 (4.70)	NR
	Placebo + EPN	15/30	NR	NR	NR	6/30	16/30	NR	27.40 (6.60)	17.60 (6.50)	NR
19	Probiotics + EPN	48/118	NR	NR	NR	NR	26/118	15/118	13.50 (12.40)	21.52 (13.51)	13.06 (9.76)
	EPN	62/117	NR	NR	NR	NR	25/117	9/117	10.60 (10.20)	30.09 (33.78)	19.46 (11.26)
20	Prebiotics + EPN	4/20	5/20	NR	0/20	NR	1/20	NR	NR	27.55 (7.80)	15.90 (9.97)
	Placebo + EPN	4/20	11/20	NR	4/20	NR	4/20	NR	NR	31.2 (15.80)	26.11 (22.94)
21	Prebiotics + EPN	7/30	NR	NR	NR	1/30	2/30	NR	24.10 (5.60)	14.20 (4.80)	16.06 (4.81)
	EPN	15/30	NR	NR	NR	10/30	6/30	NR	27.40 (6.60)	17.60 (6.70)	20.26 (6.05)
22	EPN	113/1202	173/1202	38/1202	18/1202	432/1202	498/1202	429/1202	19.10 (17.81)	10.05 (8.16)	NR
	TPN	118/1208	194/1208	55/1208	16/1208	393/1208	479/1208	405/1208	20.10 (17.81)	10.70 (8.90)	NR
23	Synbiotics + EPN	5/35	10/35	5/35	NR	2/35	3/35	NR	NR	26.56 (23.19)	NR
	EN	18/37	25/37	5/37	NR	10/37	4/37	NR	NR	30.13 (21.59)	NR
24	Probiotics + EPN	7/48	NR	NR	NR	7/48	NR	5/48	14.20 (8.60)	11.60 (8.00)	8.75 (4.79)
	Placebo + EPN	13/54	NR	NR	NR	15/54	NR	6/54	21.10 (5.70)	18.60 (6.30)	12.08 (7.13)
25	Probiotics + EPN	7/72	NR	NR	NR	NR	28/72	22/72	12.56 (9.08)	7.18 (3.40)	6.00 (3.03)
	Placebo + EPN	8/74	NR	NR	NR	NR	30/74	20/74	16.12 (13.61)	9.06 (5.29)	7.35 (5.29)
26	Probiotics + EPN	6/28	NR	NR	NR	1/28	NR	3/28	NR	NR	NR
	EPN	9/28	NR	NR	NR	7/28	NR	4/28	NR	NR	NR
27	EPN	8/78	NR	NR	NR	NR	NR	NR	18.40 (18.73)	10.16 (8.08)	NR
	TPN	10/73	NR	NR	NR	NR	NR	NR	20.65 (12.86)	12.71 (9.08)	NR
28	Probiotics + EPN	5/32	NR	NR	NR	NR	NR	11/32	NR	14.60 (4.78)	11.60 (4.78)
	Placebo + EPN	7/33	NR	NR	NR	NR	NR	12/33	NR	12.63 (3.68)	9.10 (3.64)
29	Probiotics + EPN	9/73	NR	NR	NR	NR	NR	NR	NR	13.35 (1.45)	8.19 (1.21)
	Placebo + EPN	33/74	NR	NR	NR	NR	NR	NR	NR	14.88 (1.79)	8.00 (1.51)
30	Probiotics + EPN	289/1318	414/1318	106/1318	2/1318	861/1318	363/1318	279/1318	25.85 (21.52)	12.70 (8.91)	8.05 (6.68)
	Placebo + EPN	284/1332	418/1332	101/1332	3/1332	855/1332	381/1332	296/1332	25.15 (20.04)	12.70 (7.42)	8.05 (6.68)
31	Probiotics + EPN Placebo + EPN	7/59	NR	2/59	16/59	0/59	3/59	NR	NR	NR	NR
		15/53	NR	4/53	15/53	2/53	2/53	NR	NR	NR	NR

*BSIs, bloodstream infections; EPN, enteral nutrition and/or adjuvant peripheral parenteral nutrition; LOS, length of stay; MV, mechanical ventilation; NI, nosocomial Infection; NR, not reported; SD, standard deviation; TPN, total parenteral nutrition; UTI, urinary tract infection; VAP, ventilator-associated pneumonia.*

The diagnostic criteria for VAP included Centers for Disease Control and Prevention (CDC) criteria (87), American College of Chest Physicians (ACCP) clinical Criteria (88), American Thoracic Society (ATS) and Infectious Diseases Society of America (IDSA) criteria (89), clinical pulmonary infection score (CPIS) criteria (90), and diagnostic criteria of clinical symptoms, laboratory and chest radiographic tests, and pathogenic bacteria (Supplementary Table 2.1). The diagnostic criteria for NI, BSIs and UTI used the CDC criteria or criteria of combined clinical symptoms, laboratory tests and pathogenic bacteria (Supplementary Tables 2.2, 2.3). NI included pneumonia, BSIs, catheter-related bloodstream infections (CRBSI), UTI, sepsis, surgical infections, wound infections, skin or soft-tissue infection, and other infections (Supplementary Table 2.5). The diagnostic criteria for diarrhea were duration, frequency, weight, and combination of consistency and frequency (Supplementary Table 2.4).

### Risk of Bias in Included Studies

**FIGURE 2:**
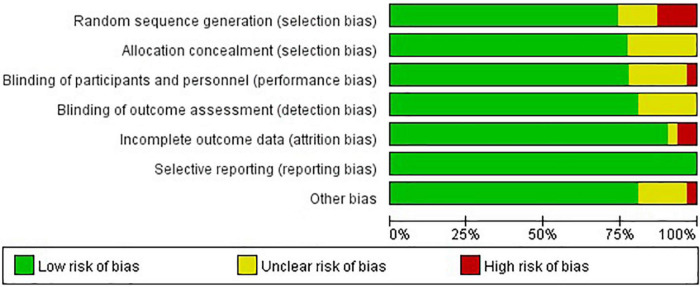
Risk of bias assessment graph for included studies. Review authors’ judgments (low, unclear, and high) for each risk of bias item shown as percentages across all included studies.

**FIGURE 3:**
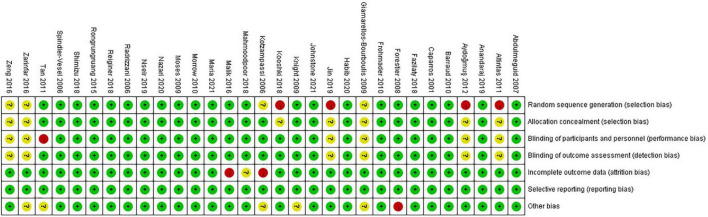
Risk of bias summary for included studies. Studies were classified as having low ROB if none was rated as high ROB, and three or less were rated as unclear risk. Studies had moderate ROB if one was rated as high ROB or none was rated as high ROB but four or more were rated as unclear risk. All other cases were assumed to pertain to high ROB.

Twenty (65%) of 31 trials were rated as low ROB, six (19%) trials were deemed high, and five (16%) were considered moderate (Figures 2, 3). Adequately random sequence generation was performed in 23 trials, unclear in four trials, and high risk in four trials. Adequate allocation concealment was reported in 24 trials, and unclear in seven trials. Blinding of participants and personnel was adequately reported in 24 trials, unclear in six trials, and high risk in one trial. Blinding of outcome assessors was adequately reported in four trials, and unclear in seven trials. Twenty-eight trials adequately addressed incomplete outcome data, and we considered two “high” risk, and the remaining study was rated as “unclear.” We did not have sufficient information to assess selective reporting bias because the protocols for the included studies were not available. Therefore, all studies were rated as “low” risk. Twenty-five trials were rated as “low” risk, five trials were rated as “unclear,” and one trial was rated as “high” risk.

### Effects of Interventions in the Network

#### Primary Outcome (Incidence of Ventilator-Associated Pneumonia)

**FIGURE 4:**
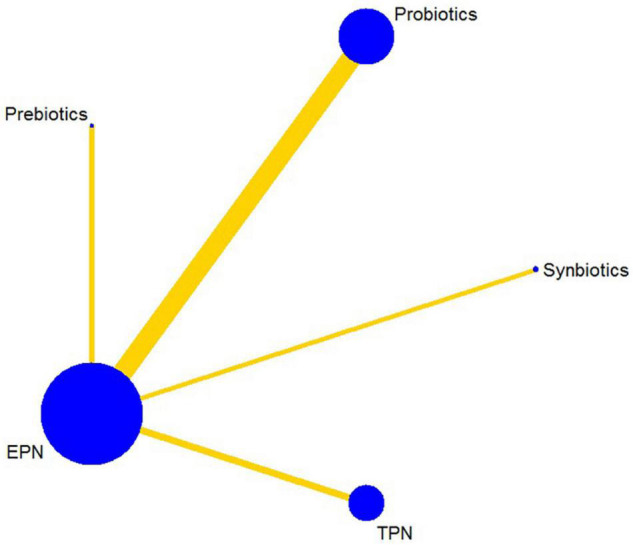
Network plot of all intervention comparisons for ventilator-associated pneumonia. The size of the nodes corresponds to the total number of participants that study the treatments. The (directly) comparable treatments are linked with a line. The thickness of the line corresponds to the standard error of trials that study this comparison. The colors of the line correspond to the quality of trials that study this comparison. Moderate risk of bias [yellow]. EPN, enteral nutrition and/or adjuvant peripheral parenteral nutrition. TPN, total parenteral nutrition.

**FIGURE 5:**
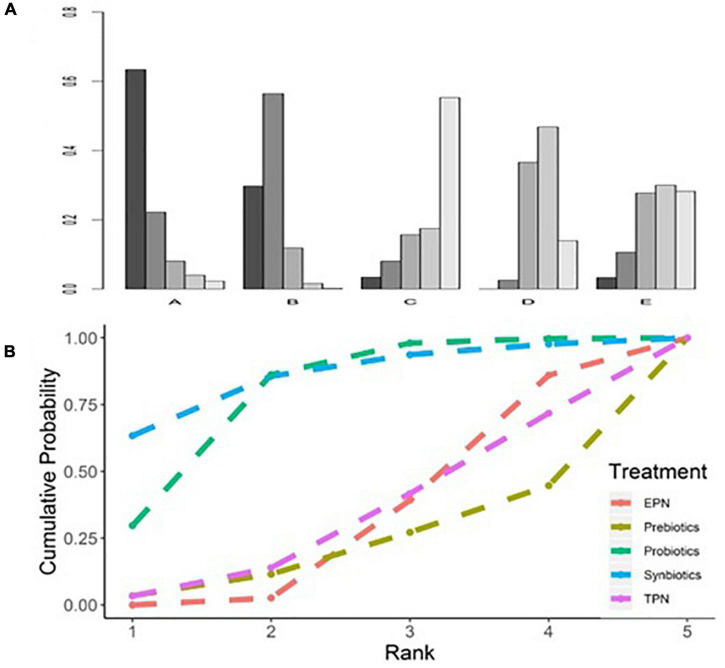
Rankogram and SUCRA ranking curve for ventilator-associated pneumonia. **(A)** Rankogram for ventilator-associated pneumonia. A = Synbiotics. B = Probiotics. C = Probiotics. D = EPN. E = TPN. **(B)** SUCRA ranking for ventilator-associated pneumonia. The number on the *X*-axis represents the rank. As the number goes up, the rating goes down. EPN, enteral nutrition and/or adjuvant peripheral parenteral nutrition. TPN, total parenteral nutrition.

The analysis of the primary outcome was based on 25 studies comprising 7721 patients. A head-to-head trial between EPN and any other intervention was obtained, but no study directly compared synbiotic-supplemented EPN therapy with TPN, prebiotic-supplemented EPN, and probiotic-supplemented EPN therapies in the network (Figure 4). Probiotic-supplemented EPN therapy was more significantly associated with a low incidence of VAP than EPN therapy (OR 0.75; 95% CrI 0.58–0.95), whereas synbiotic-supplemented EPN (OR 0.66; 95% CrI 0.37–1.15), prebiotic-supplemented EPN (OR 1.14; 95% CrI 0.63–1.98), and TPN (OR 1.01; 95% CrI 0.67–1.54) therapies were not correlated to a low incidence of VAP (Table 4). The SUCRA ranking curve showed that synbiotic-supplemented EPN and probiotic-supplemented EPN therapies were the top two treatments for VAP prevention (Figure 5).

**TABLE 4:** Results from pairwise meta-analysis and network meta-analysis on ventilator-associated pneumonia.

**Synbiotics**	−	−	2.00 (0.87, 4.80)	−

0.89 (0.48, 1.63)	**Probiotics**	−	**1.70 (1.20, 2.50)**	−
0.59 (0.26, 1.28)	0.66 (0.36, 1.24)	**Prebiotics**	0.91 (0.38, 2.30)	−
0.66 (0.37, 1.15)	**0.75 (0.58, 0.95)**	1.14 (0.63, 1.98)	**EPN**	1.10 (0.57, 2.10)
0.66 (0.32, 1.3)	0.74 (0.45, 1.2)	1.12 (0.54, 2.22)	0.99 (0.65, 1.5)	**TPN**

*Data are the OR (95% CrI) in the column-defining treatment compared with the row-defining treatment. With treatment as the boundary, the lower left part of the table is the result of network meta-analysis, and the upper right part of the table is the result of pairwise meta-analysis. For network meta-analysis, OR lower than 1 favor the column-defining treatment [e.g., column 2 vs. row 4 in the lower left part of the table (probiotics vs. EPN) is the result of network meta-analysis (OR 0.75 95% CrI 0.58–0.95), so is favor the probiotics]. For pairwise meta-analysis, OR higher than 1 favor the row-defining treatment [e.g., column 4 vs. row 2 in the upper right part of the table (EPN vs. probiotics) is the result of pairwise meta-analysis (OR 1.70 95% CrI 1.20–2.50), so is favor the probiotics]. To obtain OR for comparisons in the opposite direction, reciprocals should be taken. Significant results are in bold and underscored. OR, odds ratio; CrI, credible interval; EPN, enteral nutrition and/or adjuvant peripheral parenteral nutrition; TPN, total parenteral nutrition.*

#### Secondary Outcomes

Analyses of NI, BSIs, UTI, and diarrhea outcomes were based on 12 studies (including 6183 patients), 9 studies (including 5939 patients), 12 studies (including 6198 patients), and 15 studies (including 6439 patients), respectively. Analyses of hospital and ICU mortality outcomes were based on 18 studies (including 6998 patients) and 14 studies (including 6586 patients), respectively. Analyses of hospital LOS, ICU LOS, and MV duration were based on 17 studies (including 7021 patients), 25 studies (including 7817 patients), and 18 studies (including 4520 patients), respectively.

A head-to-head trial between EPN therapy and any other intervention was found in all networks. No study directly compared probiotic-supplemented EPN therapy with TPN and synbiotic-supplemented EPN therapies in networks of NI, BSIs, UTI, ICU mortality, ICU LOS, and MV duration. Similarly, prebiotic-supplemented EPN therapy was not directly compared with TPN and probiotic-supplemented EPN therapies in these networks (Supplementary File 4).

In terms of preventing the development of NI, synbiotic-supplemented EPN therapy was more effective than EPN therapy (OR 0.19; 95% CrI 0.08–0.47). In terms of preventing diarrhea, prebiotic-supplemented EPN therapy was more effective than EPN therapy (OR 0.05; 95% CrI 0.00–0.71). Synbiotic-supplemented EPN, probiotic-supplemented EPN, prebiotic-supplemented EPN, and TPN therapies were not correlated with the low incidence of BSIs and UTI, or the low mortality of hospital and ICU, compared with EPN therapy. Similarly, these therapies were not correlated with the shorter length of hospital stay, length of ICU stay, and duration of MV, compared with EPN therapy.

**FIGURE 6:**
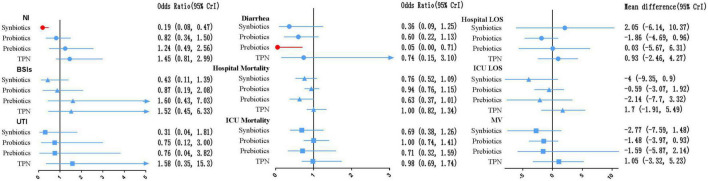
Forest plot of the effect estimate for each active intervention vs. EPN on secondary outcomes. Estimates are presented as odds ratios (OR) or mean difference (MD) and 95% CrI. OR < 1 favor the treatment. MD < 0 favor the treatment. BSIs, bloodstream infections; CrI, credible interval; EPN, enteral nutrition and/or adjuvant peripheral parenteral nutrition; LOS, length of stay; MV, duration of mechanical ventilation; NI, nosocomial infections; TPN, total parenteral nutrition; UTI, urinary tract infection.

The SUCRA ranking curve showed that synbiotic-supplemented EPN therapy ranked first among all therapies in networks of NI, BSIs, UTI, ICU mortality, ICU LOS, and MV duration. Prebiotic-supplemented EPN therapy ranked first among all therapies in networks of diarrhea and hospital mortality. Probiotic-supplemented EPN therapy ranked first among all therapies in the network of hospital LOS. Figure 6 and Supplementary Files 3, 9 detail the results.

### Direct Meta-analysis

Table 4 shows the results of the pairwise on VAP. The data of pairwise meta-analysis for all secondary outcomes are shown in Supplementary File 3.

### Heterogeneity, Inconsistency and Transitivity

Heterogeneity analyses revealed moderate-to-high global heterogeneity for VAP incidence (*I^2^* = 73.43%), NI incidence (*I^2^* = 58.27%), diarrhea incidence (*I^2^* = 84.83%), hospital LOS (*I^2^* = 74.57%), ICU LOS (*I^2^* = 83.60%) and MV duration (*I^2^* = 90.55%) (Supplementary Figure 5.1).

Global inconsistency and inconsistencies between indirect and direct comparisons were not found for all outcomes (Supplementary Figure 5.2).

Transitivity assessment for primary outcome showed that the mean age among all comparisons was the same (Supplementary Figure 6.1). In addition, the mean severity of illness at baseline showed a relatively high mean APACHE II score for the comparison between prebiotics and EPN (Supplementary Figure 6.2).

### Subgroup Analyses for the Incidence of Ventilator-Associated Pneumonia

Analyses in the subgroups of general ICU patients and trauma patients subgroup were based on 19 studies (including 7250 patients), and 6 studies (including 471 patients), respectively. Analyses in the subgroups of higher disease severity and lower disease severity were based on 11 studies (including 6061 patients) and 11 studies (including 1506 patients), respectively. Analyses in the subgroups of only *Lactobacillus* and a mixture of strains were based on 14 studies (including 6256 patients), and 19 studies (including 4504 patients), respectively. Analyses in the subgroups of low-dose and high-dose were based on 16 studies (including 3917 patients), and 16 studies (including 6783 patients), respectively. Analysis in the subgroup of high-quality studies was based on 16 studies (including 6864 patients). Analyses in the subgroups of nutrition therapy within 24 h, nutrition therapy within 48 h, and nutrition therapy beyond 48 h were based on 9 studies (including 3808 patients), 15 studies (including 4205 patients), and 4 studies (including 2841 patients), respectively.

In terms of trauma patients, probiotic-supplemented EPN therapy was significantly associated with a low incidence of VAP, in contrast to EPN therapy (OR 0.30; 95% CrI 0.13–0.83). Similarly, probiotic-supplemented EPN therapy was significantly associated with a low incidence of VAP in the routine low-dose subgroup (OR 0.17; 95% CrI 0.03–0.73) and the mixed probiotic strain subgroup (OR 0.55; 95% CrI 0.31–0.97), in contrast to EPN therapy. In terms of other subgroups, synbiotic-supplemented EPN, probiotic-supplemented EPN, prebiotic-supplemented EPN, and TPN therapies were not associated with a low incidence of VAP, in contrast to EPN therapy. Similarly, these therapies were not correlated with the shorter length of hospital stay, length of ICU stay, and duration of MV, compared with EPN therapy (Table 5).

**TABLE 5:** Subgroup analyses for ventilator-associated pneumonia.

	Synbiotics	Probiotics	Prebiotics	EPN	TPN	Number of studies	Participants
	**OR (95% CrI)**	**OR (95% CrI)**	**OR (95% CrI)**	**OR (95% CrI)**	**OR (95% CrI)**		
Overall patients	0.66 (0.37, 1.15) Rank 1	**0.75 (0.58, 0.95)** Rank 2	1.14 (0.63, 1.98) Rank 5	reference Rank 3	1.01 (0.67, 1.54) Rank 4	25	7721
General ICU patients	0.50 (0.20, 1.18) Rank 1	0.65 (0.41, 1.00) Rank 2	1.34 (0.54, 3.01) Rank 5	reference Rank 3	1.10 (0.56, 2.18) Rank 4	19	7250
Trauma patients	0.90 (0.15, 5.40) Rank 2	**0.30 (0.13, 0.83)** Rank 1	0.99 (0.10, 9.54) Rank 4	reference Rank 3	−	6	471
Higher disease severity	−	0.65 (0.31, 1.31) Rank 2	0.48 (0.11, 2.12) Rank 1	reference Rank 3	1.16 (0.41, 3.48) Rank 4	11	6061
Lower disease severity	0.51 (0.24, 1.03) Rank 1	0.61 (0.37, 1.02) Rank 2	2.64 (0.91, 7.60) Rank 5	reference Rank 3	1.37 (0.39, 5.24) Rank 4	11	1506
Only *Lactobacillus* GG	−	0.66 (0.36, 1.12) Rank 1	1.14 (0.45, 2.62) Rank 4	Reference Rank 2	1.09 (0.57, 2.11) Rank 3	14	6256
Mixed strains	0.49 (0.19, 1.26) Rank 1	**0.55 (0.31, 0.97)** Rank 2	1.07 (0.38, 2.78) Rank 3	Reference Rank 4	1.10 (0.53, 2.34) Rank 5	19	4504
Low-dose	**0.17 (0.03, 0.73)** Rank 1	0.65 (0.37, 1.07) Rank 2	1.21 (0.49, 2.54) Rank 5	Reference Rank 3	1.09 (0.62, 1.97) Rank 4	16	3917
High-dose	0.76 (0.24, 2.48) Rank 2	0.60 (0.30, 1.12) Rank 1	1.06 (0, 37, 2.89) Rank 4	Reference Rank 3	1.10 (0.51, 2.37) Rank 5	16	6783
High quality studies only (low risk of bias)	0.37 (0.12, 1.10) Rank 1	0.61 (0.36, 1.01) Rank 2	2.00 (0.58, 6.17) Rank 5	Reference Rank 3	1.02 (0.43, 2.45) Rank 4	16	6864
Nutrition therapy within 24 h	0.76 (0.23, 2.61) Rank 1	0.78 (0.36, 1.62) Rank 2	1.09 (0.29, 3.42) Rank 4	Reference Rank 3	−	9	3808
Nutrition therapy within 48 h	0.76 (0.26, 2.20) Rank 2	0.69 (0.39, 1.13) Rank 1	1.12 (0,42, 2.71) Rank 5	Reference Rank 3	1.12 (0.35, 3.68) Rank 4	15	4205
Nutrition therapy beyond 48 h	0.16 (0.02, 1.24) Rank 1	1.06 (0.17, 6.80) Rank 2	−	Reference Rank 3	1.12 (0.32, 4.46) Rank 4	4	2841

*Significant results are in bold and underscored. OR, odds ratio; CrI, credible interval; EPN, enteral nutrition and/or adjuvant peripheral parenteral nutrition; TPN, total parenteral nutrition.*

### Sensitivity Analyses for the Incidence of Ventilator-Associated Pneumonia

Analyses of studies with low-moderate ROB and studies with robust diagnostic criteria for VAP were based on 20 studies (including 7439 patients), and 23 studies (including 7596 patients), respectively. Analyses of multicentric studies and single-center studies were based on 8 studies (including 5920 patients) and 17 studies (including 1801 patients), respectively. In terms of studies with low-moderate ROB and studies with robust diagnostic criteria for VAP, probiotic-supplemented EPN therapy remained significantly associated with low VAP incidence, in contrast to EPN therapy, whereas synbiotic-supplemented EPN, prebiotic-supplemented EPN, and TPN therapies were not correlated with a low incidence of VAP. No significant differences were found among the five therapies for incidence of VAP in multicentric studies and single-center studies. The data are shown in Supplementary File 10.

### Grades of Recommendation, Assessment, Development and Evaluation Assessments

Publication bias was found in the incidence of VAP and NI, hospital mortality, hospital and ICU LOS, and MV duration (Supplementary File 7).

In summary, the GRADE scores of the relative therapeutic effects and ranking for VAP suggested that the certainty of evidence varied. Comparisons between synbiotic-supplemented EPN and probiotic-supplemented EPN therapies, synbiotic-supplemented EPN and prebiotic-supplemented EPN therapies, and probiotics-supplemented EPN and TPN therapies were high, whereas those between synbiotics-supplemented EPN and EPN therapies and between prebiotic-supplemented EPN and TPN therapies were low. Moreover, the comparison between probiotic-supplemented EPN and TPN therapies was very low, and other comparisons were moderate. The ranking of treatment was low. Downgrading was due to imprecision, publication bias, or inconsistency (Table 6). Supplementary File 8 presents the GRADE and ranking of treatment for all secondary outcomes.

**TABLE 6:** Result of GRADE for nosocomial infection.

	Nature of the evidence	Study limitations	Imprecision	Inconsistency	Indirectness	Publication bias	Confidence	Downgrading due to
A vs. B	Indirect estimated	No downgrade	No downgrade	No downgrade	No downgrade	No downgrade	HIGH	−
A vs. C	Indirect estimated	No downgrade	No downgrade	No downgrade	No downgrade	No downgrade	HIGH	−
A vs. D	Mixed estimated	No downgrade	Downgrade because point estimate >1.0 but lower limit <0.80	Downgrade because pair heterogeneity I^2^ = 62.7%	No downgrade	No downgrade	LOW	Imprecision Inconsistency
A vs. E	Indirect estimated	No downgrade	Downgrade because point estimate >1.0 but lower limit <0.80	No downgrade	No downgrade	No downgrade	MODERATE	Imprecision
B vs. C	Indirect estimated	No downgrade	Downgrade because point estimate >1.0 but lower limit <0.80	No downgrade	No downgrade	No downgrade	MODERATE	Imprecision
B vs. D	Mixed estimated	No downgrade	Downgrade because point estimate >1.0 but lower limit <0.80	No downgrade Downgrade because pair heterogeneity I^2^ = 77.7%	No downgrade	Downgrade	VERY LOW	Imprecision Inconsistency Publication bias
B vs. E	Indirect estimated	No downgrade	No downgrade	No downgrade	No downgrade	No downgrade	HIGH	−
C vs. D	Mixed estimated	No downgrade	Downgrade because point estimate >1.0 but lower limit <0.80	Downgrade because pair heterogeneity I^2^ = 84.4%	No downgrade	No downgrade	LOW	Imprecision Inconsistency
C vs. E	Indirect estimated	No downgrade	No downgrade	No downgrade	No downgrade	Downgrade	MODERATE	Publication bias
D vs. E	Mixed estimated	No downgrade	Downgrade because point estimate <1.0 but upper limit >1.25	No downgrade	No downgrade	No downgrade	MODERATE	Imprecision
Ranking of treatments		No downgrade	No downgrade	Downgrade because global heterogeneity I^2^ = 73.43%	No downgrade	Downgrade	LOW	Inconsistency Publication bias

*A, synbiotics; B, probiotics; C, prebiotics; D, enteral nutrition and/or adjuvant peripheral parenteral nutrition; E, total parenteral nutrition.*

## Discussion

### Summary of Main Results

This systematic review evaluated the effects of TPN and EPN supplemented with or without probiotic, prebiotic, and synbiotic therapies on VAP, using 31 RCTs (including 8339 patients). Overall, the results of NMA indicated that probiotic supplementation was significantly associated with increased incidence of VAP in critically invasive mechanically ventilated patients. This result was consistent with previous RCTs (14, 34, 36, 37, 91, 92) and meta-analysis (38–42). Subgroup analysis showed that probiotic supplementation therapy significantly prevented the incidence of VAP in trauma patients. Mixed strains and low-dose probiotic therapies were associated with a low incidence of VAP. Moreover, this NMA found that synbiotic supplementation therapy was significantly related to decreased incidence of NI and prebiotics supplementation were the most effective in preventing diarrhea.

### Applicability of Evidence

The availability of evidence that probiotic supplementation alleviates VAP in critically ill patients was influenced by several complex risk factors. The possible underlying mechanism areas were discussed below: First, probiotic supplementation may maintain the intestinal microbiota. Probiotic therapy increases the number of intestinal microbiota while increasing their genus groups and promoting the growth of other microbiota (14). Second, probiotic supplementation increases the nutritional support of host epithelial cells. Probiotic supplementation significantly increases the levels of short-chain fatty acids, especially acetate, which provides an additional energy source for intestinal epithelial cells and may attenuate the occurrence of VAP (93). Third, probiotic supplements maintain the intestinal epithelial barrier. Probiotic supplementation may inhibit the release of enteric toxins and maintain tight connections by promoting an increase in acetate and lactate levels (94). Finally, probiotics regulate innate and adaptive immune systems, which in turn promote extra-intestinal organ function and reduce systemic inflammation (94).

### Disagreements With Other Studies

Notably, the primary finding was inconsistent with the results of previous meta-analysis (59, 95, 96) and RCTs (43, 44, 46, 49, 50, 52, 54, 97), which showed that probiotics cannot alleviate VAP in invasive mechanically ventilated patients. The reasons that probiotic supplementation did not improve VAP were complex, and we believe that the following reasons can be discussed: First, the principal limitation of these studies stems from small sample size. The sample sizes used by Tan et al. (44), Habib et al. (46), and Jin et al. (97) were all less than 100. Increasing the population size may provide additional information about the prevention of VAP (98). In addition, Barraud et al. (50) showed that 740 patients are needed to demonstrate the benefits of probiotics, but their study was prematurely stopped after an interim analysis because of safety concerns. Only 167 patients were actually enrolled in the study. Second, some large clinical trials used a single probiotic strain. Only *Lactobacillus rhamnosus* was used by Johnstone et al. (43) and Anandaraji et al. (54). Accumulating evidence shows that different strains of probiotics exert beneficial effects through multiple mechanisms and have synergistic effects when supplemented as combinations of strains (99). Third, the improving effects of probiotics may vary with the study population. Jin et al. (97) showed that probiotics prevented the incidence of VAP in stroke patients, Tan et al. (44) showed an opposite result in patients with traumatic brain injuries. Therefore, the effect of critically ill population heterogeneity on probiotics should be fully considered.

### Analysis of Subgroup Results

The results of the subgroup analysis were as follows: First, probiotic-supplemented therapy had a significant effect on preventing the incidence of VAP in trauma patients and ranked first. Major traumatic injuries influence intestinal microbiota inhibiting the proliferation of beneficial commensal bacteria by inducing the overgrowth of pathogenic bacteria (100), and leading to changes in immune function after trauma (4). Starting probiotic therapy immediately can minimize dysbiosis. Probiotics may play a potential suppressive role in gut inflammation by strengthening intestinal mucosal and epithelial barriers and antagonizing the colonization of virulent species (101). Future high-quality RCTs with large sample sizes should focus on trauma patients. Second, mixed probiotic strain therapy was associated with low VAP. Gut dysbiosis involves loss of diversity and abundance and altered metabolic capacity of a flora. The depletion of beneficial commensal bacteria such as *Bifidobacterium* and *Lactobacillus* was significantly associated with these effects (17). Probiotic strains had a greater effect on ecological performance possibly because of competitive interactions among the members of the microbiota (102). Some clinical therapeutic benefits are likely derived from shared mechanisms, suggesting that probiotic effects may be subspecies specific, species-specific, or genus specific (99, 103). Third, low-dose probiotic therapy was associated with a lower incidence of VAP. The physiologically required administration of probiotics seems to be associated with a low incidence of infectious complications, whereas the administration of excessive probiotic microorganisms led to an increase in infectious complications due to bacteremia and fungemia in critically ill adults, postoperative, and immuno-compromised patients (22). Hence, in our view, the safety of probiotics in future RCTs should be explored, particularly in specialized critically ill patients. Furthermore, disease severity at baseline has found no association between probiotic-supplemented EPN therapy and a low incidence of VAP as compared to EPN therapy. Good scoring systems should be able to predict the severity of disease and functional status, be well-calibrated, validated and highly discernible, and be applicable to all the general population of critically ill patients. However, APACHE II and SAPS II scoring predictions are very complex, requiring multiple variables to calculate their score (104). Their cut-offs, sensitivity, and specificity for predicting the mortality risk of ICU patients vary with patient populations (79). In addition, they are weak to measure organ dysfunction (105) and are affected by lead-time bias and treatment (106, 107). For these reasons, the above results should be interpreted with caution.

### Analysis for Secondary Outcomes

Results of analysis for secondary outcomes were as follows: In terms of preventing the incidence of NI, synbiotic-supplemented EPN therapy showed a better effect than EPN therapy. However, these findings may be inconclusive. Although these results were consistent with our previous results (59), they should be interpreted with caution. The analysis involved only three small-sample studies involving synbiotics. Among them, Spindler-Vesel et al.’s (108) and Kotzampassi et al.’s studies (109) involving trauma patients and Shimizu et al.’s study (14) involving sepsis patients suggested that synbiotics supplementation can alleviate NI. They indicated that synbiotic-supplemented therapy should be considered to prevent NI in specific invasive mechanically ventilated patients. Additionally, in terms of preventing the incidence of diarrhea, prebiotics-supplemented EPN therapy showed a better effect than EPN therapy. However, these findings may be equally inconclusive and should be interpreted with caution. The diagnostic criteria for diarrhea were based on the original literature, which was based on duration, frequency, weight, and the combination of consistency and frequency. Given that ensuring consistency among different definitions is difficult (110), the incidence of diarrhea varied according to the diagnostic criterion used for calculations. In addition, owing to the limited number of studies, we were unable to perform further grouping analysis.

### Strengths of This Network Meta-analysis

This study had four strengths. Firstly, this is the first NMA that is based on a Bayesian framework and that assessed the relative effectiveness of different symbiotic regimens for alleviating VAP in critically invasive mechanically ventilated patients. The results of this study can help clinicians identify differences in relative efficacy among treatments without head-to-head comparison. Second, this study offered the most updated assessment of symbiotic therapy for patients with critically invasive mechanically ventilated patients. A structured search strategy was used in retrieving all published studies since 2000, focusing on symbiotic therapy for improving VAP. Third, this study examined the largest number of studies on symbiotic therapy (31 RCTs) from 16 countries in Europe, America, Asia, and Africa and enrolled 8339 patients. Fourth, this study evaluated several relevant important clinical outcomes among critically invasive mechanically ventilated patients, including the incidence of NI, the incidence of BSIs, the incidence of UTI, the incidence of diarrhea, mortality, LOS, and MV duration. Hence, our study is of great value to clinicians exploring the characteristics of different therapies. Lastly, sensitivity and subgroup analysis provided evidence of the robustness of estimates.

### Limitations of This Network Meta-analysis

This study had several limitations. First, patients with sepsis, shock, severe multiple injuries, severe traumatic brain injury, severe stroke, acute organophosphorus poisoning, and general ICU were enrolled in this study. These patients presented significant differences in pathogenic factors, pathophysiology, and clinical manifestations. Second, the definitions of VAP in the included studies varied. Ensuring the consistency of different definitions was difficult. Given that these criteria vary in sensitivity and accuracy for diagnosing VAP (111), reliance upon different criteria may result in potential variations in the incidence of VAP (2). Some included studies did not provide an accurate definition of secondary outcomes, such as incidence of diarrhea, and were inconsistent because they were vague (112) and subject to different interpretations (110). Third, variations in dose, genus, species, strain, and duration of probiotics were found among the studies. Furthermore, the gastrointestinal conditions of patients before the treatment could contribute to the heterogeneity of the patients and affect the treatment efficacy. The aforementioned four limitations were common among the studies in this field, which may contribute to the slight heterogeneity (13, 40, 113, 114). These heterogeneities could not be eliminated by statistical methods. To decrease these heterogeneities among the studies, whenever possible, we performed subgroup analysis based on population, disease severity, dose, strains, the timing of initial nutrition, and study quality. Nevertheless, more subgroup analyses were limited by the small number of studies. The results of subgroup analysis showed that these heterogeneities were significantly decreased. Similarly, sensitivity analyses of studies with low-moderate ROB and studies with robust diagnostic criteria for VAP showed that the conclusions concerning the overall effects of probiotics were robust. Overall, even the Cochrane review, which is internationally recognized as the gold standard of evidence-based information in health care, could not eliminate the effect of these heterogeneities (113, 114). Because of this, none of the recommendations that advocated probiotics supplementation in critically ill patients in evidence-based guidelines were actually based on high-quality evidence. Clinical evidence surrounding the impact of the aforementioned limitations on the treatment effects of probiotics is the focus of this field, and future clinical studies and reviews should focus on addressing this issue.

Beyond this, other potential limitations should also be concerned: Nearly 90% of all included studies were from Europe and Asia, and thus our ability to generalize the results of this study to all patient populations was limited. Antibiotic consumption and length of antibiotic therapy were not assessed because of inadequate information and inconsistent reporting across trials. Based on the GREAD system, confidence in the estimates was low or very low, restricting the interpretation and evaluation in further clinical practice.

### Suggestions and Perspectives

In further works, the mechanisms of probiotics and hosts need to be further explored and clarified. Well-conducted, large-scale, multicenter, concealed, and stratified RCTs focus on specific populations, such as trauma, sepsis, and high infectious risk or high antimicrobial exposure patients, are needed to confirm these findings. Factors, including probiotic genus, species, strain, optimal dose, route, and duration of administration, should be carefully considered in the evaluation of the effectiveness in alleviating VAP.

Postbiotics are inanimate microorganisms and/or their components confer health benefits on hosts (115). They improve the immune system in animal experiments and may play important roles in antimicrobial and targeted anti-inflammatory activities and immune response modulation and influence intestinal secretion and movement. Moreover, they exert beneficial metabolic effects through interactions with dietary components. We are looking forward to high-quality human clinical RCTs to provide the ultimate proof (115).

## Conclusion

Based on pooled results, this study suggests that probiotic supplementation shows promise in reducing the incidence of VAP in critically invasive mechanically ventilated patients. Probiotic supplementation for trauma patients, as well as routine low-dose and mixed probiotic strains supplementation, seems to be plausible. Currently, low quality of evidence reduces strong clinical recommendations. Further high-quality RCTs are needed to conclusively prove these findings.

## Data Availability Statement

The original contributions presented in this study are included in the article/Supplementary Material, further inquiries can be directed to the corresponding author/s.

## Author Contributions

CL and NX had the idea for and designed the study. NX supervised the study. CL, NX, JM, FL, and JC did search clinical trials, study select, data extract, and statistical analysis. CL wrote the manuscript. All authors contributed to acquisition, analysis, interpretation of data, revised the report, and approved the final version before submission.

## Conflict of Interest

The authors declare that the research was conducted in the absence of any commercial or financial relationships that could be construed as a potential conflict of interest.

## Publisher’s Note

All claims expressed in this article are solely those of the authors and do not necessarily represent those of their affiliated organizations, or those of the publisher, the editors and the reviewers. Any product that may be evaluated in this article, or claim that may be made by its manufacturer, is not guaranteed or endorsed by the publisher.

## Footnotes

1
https://clinicaltrials.gov/

2
http://www.chictr.org.cn/index.aspx

3
http://www.who.int/ictrp/en/

4
https://www.isrctn.com/

5
http://www.anzctr.org.au/

## Supplementary Material

Supplementary File 1 Search strategy.pdf.

Supplementary File 2 Diagnostic criteria for outcomes.pdf.

Supplementary File 3 Results of meta-analysis of pairwise comparisons and network.pdf.

Supplementary File 4 Network plot of all intervention comparisons.pdf.

Supplementary File 5 Assessment of heterogeneity and inconsistency in networks.pdf.

Supplementary File 6 Assessment of transitivity in networks for primary outcome.pdf.

Supplementary File 7 Comparison-adjusted funnel plot for the network.pdf.

Supplementary File 8 GRADE for each outcome in networks.pdf.

Supplementary File 9 Treatment ranking and SUCRA ranking curve for each outcome.pdf.

Supplementary File 10 Sensitivity analyses for the risk of primary outcome.pdf.

## Abbreviations

BSIs: bloodstream infections
CENTRAL: Cochrane Central Register for Controlled Trials
CFU: colony-forming units
CRBSI: catheter-related bloodstream infections
CrI: credible interval
DB: double-blind
EN: enteral nutrition
EPN: enteral nutrition and/or adjuvant peripheral parenteral nutrition
GCS: Glasgow coma scale
GRADE: Grades of Recommendation, Assessment, Development and Evaluation
ICU: intensive care unit
LOS: length of stay
MC: multicenter
MD: mean difference
MV: mechanical ventilation
NI: nosocomial infection
NMA: network meta-analysis
NR: not reported
OP: open study
OR: odds ratio
PN: parenteral nutrition
PRISMA: Preferred Reporting Items for Systematic Review and Meta-analysis
PROSPERO: prospective register of systematic reviews
RCTs: randomized controlled trial studies
SB: single-blind
SC: single-center
SD: standard deviation
SUCRA: surface under the cumulative ranking curve
TPN: total parenteral nutrition
UTI: urinary tract infection
VAP: ventilator-associated pneumonia.
