# Supplementary material for: Probiotic Supplementation Prevents the Development of Ventilator-Associated Pneumonia for Mechanically Ventilated ICU Patients: A Systematic Review and Network Meta-analysis of Randomized Controlled Trials
Source: Front Nutr. 2022 Jul 8;9:919156. doi: 10.3389/fnut.2022.919156 (PMC9307490; doi:10.3389/fnut.2022.919156)
Supplement: Supplementary File 1 — Search strategy.pdf. [file Data_Sheet_1.PDF]

Supplementary 1

Search strategy for pubmed

1) **(((("Critical illness"[MeSH Terms]) OR (Critical illness*[Title/Abstract])) OR (illness, critical[Title/Abstract])) OR (illnesses, critical[Title/Abstract])) OR (Critically ill*[Title/Abstract])**
2) **(((("Intensive care units"[MeSH Terms]) OR (Intensive care unit*[Title/Abstract])) OR (unit,intensive care[Title/Abstract])) OR (ICU intensive care units[Title/Abstract])) OR (ICU[Title/Abstract])**
3) **(((((((("Critical Care"[MeSH Terms]) OR (Critical Care[Title/Abstract])) OR (care, critical[Title/Abstract])) OR (intensive care[Title/Abstract])) OR (care, intensive[Title/Abstract])) OR (surgical intensive care[Title/Abstract])) OR (care, surgical intensive[Title/Abstract])) OR (intensive care, surgical[Title/Abstract])) OR (SICU[Title/Abstract])**
4) **(((((("Coronary care units"[MeSH Terms]) OR (Care unit, coronary[Title/Abstract])) OR (Care units, coronary[Title/Abstract])) OR (Coronary care unit*[Title/Abstract])) OR (Unit,coronary care[Title/Abstract])) OR (Units, coronary care[Title/Abstract])) OR (CCU[Title/Abstract])**
5) **(((((("Respiratory care units"[MeSH Terms]) OR (Care unit, respiratory[Title/Abstract])) OR (Care units, respiratory[Title/Abstract])) OR (respiratory care unit*[Title/Abstract])) OR (Unit, respiratory care[Title/Abstract])) OR (Units, respiratory care[Title/Abstract])) OR (RICU[Title/Abstract])**
6) **(medical intensive Care Unit*[Title/Abstract]) OR (MICU[Title/Abstract])**
7) **(Emergency intensive Care Unit*[Title/Abstract]) OR (EICU[Title/Abstract])**
8) **((((((("Burn units"[MeSH Terms]) OR (Burn unit*[Title/Abstract])) OR (Unit,burn[Title/Abstract])) OR (units,burn[Title/Abstract])) OR (burn center*[Title/Abstract])) OR (center,burn[Title/Abstract])) OR (centers,burn[Title/Abstract])) OR (burn intensive care unit*[Title/Abstract])**
9) **(Nephrotic intensive care unit*[Title/Abstract]) OR (UICU[Title/Abstract])**
10) **(Obstetric intensive care unit*[Title/Abstract]) OR (OICU[Title/Abstract])**
11) **(Anesthesia intensive care unit*[Title/Abstract]) OR (AICU[Title/Abstract])**
12) **(Transplant intensive care unit*[Title/Abstract]) OR (TICU[Title/Abstract])**
13) **(Cardiopulmonary intensive care unit*[Title/Abstract]) OR (CPICU[Title/Abstract])**
14) **(Cardiac surgery intensive care unit*[Title/Abstract]) OR (CSICU[Title/Abstract])**
15) **(Neurosurgical intensive care unit*[Title/Abstract]) OR (NSICU[Title/Abstract])**
16) **(Neurological intensive care unit*[Title/Abstract]) OR (NICU[Title/Abstract])**
17) **(stroke intensive care unit*[Title/Abstract]) OR (stroke ICU[Title/Abstract])**
18) **(General intensive care unit*[Title/Abstract]) OR (GICU[Title/Abstract])**
19) **(((((("Multiple Organ Failure"[MeSH Terms]) OR (Multiple Organ Dysfunction Syndrome[Title/Abstract])) OR (Organ Failure, Multiple[Title/Abstract])) OR (Failure, Multiple Organ[Title/Abstract])) OR (Multiple Organ Failure*[Title/Abstract])) OR (MODS[Title/Abstract])) OR (Organ Dysfunction Syndrome, Multiple[Title/Abstract])**

20) **((((((("Respiration, Artificial"[MeSH Terms]) OR (mechanical ventilation*[Title/Abstract])) OR (Artificial Respiration*[Title/Abstract])) OR (Respiration, Artificial[Title/Abstract])) OR (Respirations, Artificial[Title/Abstract])) OR (Ventilation, Mechanical[Title/Abstract])) OR (Ventilations, Mechanical[Title/Abstract])) OR (MV[Title/Abstract])**
21) **(((((("Intubation, intratracheal"[MeSH Terms]) OR (intratracheal intubation*[Title/Abstract])) OR (Intubation, intratracheal[Title/Abstract])) OR (intubations, intratracheal[Title/Abstract])) OR (intubation, endotracheal[Title/Abstract])) OR (endotracheal intubation*[Title/Abstract])) OR (intubations, endotracheal[Title/Abstract])**
22) **Or/1-21**
23) **("Synbiotics"[MeSH Terms]) OR (synbiotic*[Title/Abstract])**
24) **("Probiotics"[MeSH Terms]) OR (Probiotic*[Title/Abstract])**
25) **((("Bifidobacterium"[MeSH Terms]) OR (Bifidobacterium[Title/Abstract])) OR (bifidus[Title/Abstract])) OR (bifidobacter*[Title/Abstract]);**
26) **((((((("Lactobacillus"[MeSH Terms]) OR (Lactobacillus[Title/Abstract])) OR (Lactobacil*[Title/Abstract])) OR (Lacteol[Title/Abstract])) OR (Calcium Carbonate[Title/Abstract])) OR (Drug Combinations[Title/Abstract])) OR (Lactose[Title/Abstract])) OR (Lacteol[Supplementary Concept])**
27) **("streptococcus"[MeSH Terms]) OR (streptococc*[Title/Abstract])**
28) **("lactococcus"[MeSH Terms]) OR (lactococc*[Title/Abstract])**
29) **("leuconostoc"[MeSH Terms]) OR (leuconostoc[Title/Abstract])**
30) **("pediococcus"[MeSH Terms]) OR (pediococc*[Title/Abstract])**
31) **("bacillus"[MeSH Terms]) OR (Bacillus bacterium[Title/Abstract])**
32) **(("Enterococcus"[MeSH Terms]) OR (Enterococcus[Title/Abstract])) OR (enterococcus faec*[Title/Abstract])**
33) **("Saccharomyces"[MeSH Terms]) OR (Saccharomyce*[Title/Abstract])**
34) **("Clostridium"[MeSH Terms]) OR (Clostridium[Title/Abstract])**
35) **Propionibacterrium[Title/Abstract]**
36) **("yeasts"[MeSH Terms]) OR (yeast*[Title/Abstract])**
37) **(((((((((("Fermented Foods and Beverages"[MeSH Terms]) OR (Fermented Foods[Title/Abstract] AND Beverages[Title/Abstract])) OR (Fermented Beverage*[Title/Abstract])) OR (Beverage, Fermented[Title/Abstract])) OR (Beverages, Fermented[Title/Abstract])) OR (Fermented Food*[Title/Abstract])) OR (Food, Fermented[Title/Abstract])) OR (Foods, Fermented[Title/Abstract])) OR (Cultured Food*[Title/Abstract])) OR (Food, Cultured[Title/Abstract])) OR (Foods, Cultured[Title/Abstract])**
38) **((((((((((((((("Cultured Milk Products"[MeSH Terms]) OR (Cultured Milk Product*[Title/Abstract])) OR (Milk Product, Cultured[Title/Abstract])) OR (Milk Products, Cultured[Title/Abstract])) OR (Product, Cultured Milk[Title/Abstract])) OR (Products, Cultured Milk[Title/Abstract])) OR (Fermented Milk Product*[Title/Abstract])) OR (Milk Product, Fermented[Title/Abstract])) OR (Milk Products, Fermented[Title/Abstract])) OR (Product, Fermented Milk[Title/Abstract])) OR (Products, Fermented Milk[Title/Abstract])) OR (Fermented Dairy**

Product*[Title/Abstract])) OR (Dairy Product, Fermented[Title/Abstract])) OR (Dairy Products, Fermented[Title/Abstract])) OR (Product, Fermented Dairy[Title/Abstract])) OR (Products, Fermented Dairy[Title/Abstract])

39) **((("Buttermilk"[MeSH Terms]) OR (Buttermilk[Title/Abstract])) OR (Butter milk[Title/Abstract])) OR (milk, butter[Title/Abstract])**

40) **(("Yogurt"[MeSH Terms]) OR (Yogurt[Title/Abstract])) OR (yoghurt[Title/Abstract])**

41) **(((("Kefir"[MeSH Terms]) OR (Kefir[Title/Abstract])) OR (Kefir Grain*[Title/Abstract])) OR (Grain, Kefir[Title/Abstract])) OR (Grains, Kefir[Title/Abstract])**

42) **((((((((((((((((((((((((("Fecal Microbiota Transplantation"[MeSH Terms]) OR (Fecal Microbiota Transplantation*[Title/Abstract])) OR (Microbiota Transplantation, Fecal[Title/Abstract])) OR (Microbiota Transplantations, Fecal[Title/Abstract])) OR (Transplantation, Fecal Microbiota[Title/Abstract])) OR (Transplantations, Fecal Microbiota[Title/Abstract])) OR (Intestinal Microbiota Transfer*[Title/Abstract])) OR (Microbiota Transfer, Intestinal[Title/Abstract])) OR (Microbiota Transfers, Intestinal[Title/Abstract])) OR (Transfer, Intestinal Microbiota[Title/Abstract])) OR (Transfers, Intestinal Microbiota[Title/Abstract])) OR (Fecal Transplantation*[Title/Abstract])) OR (Transplantation, Fecal[Title/Abstract])) OR (Transplantations, Fecal[Title/Abstract])) OR (Fecal Transplant*[Title/Abstract])) OR (Transplant, Fecal[Title/Abstract])) OR (Transplants, Fecal[Title/Abstract])) OR (Donor Feces Infusion*[Title/Abstract])) OR (Feces Infusion, Donor[Title/Abstract])) OR (Feces Infusions, Donor[Title/Abstract])) OR (Infusion, Donor Feces*[Title/Abstract])) OR (FMT[Title/Abstract])) OR (fecal suspension[Title/Abstract])) OR (fecal transfer[Title/Abstract])) OR (fecal infusion[Title/Abstract])) OR (bacteriotherapy[Title/Abstract])) OR (fecal donation[Title/Abstract])) OR (selective decontamination[Title/Abstract])**

43) **("Prebiotics"[MeSH Terms]) OR (Prebiotic*[Title/Abstract])**

44) **((((((((((((((((((((((("oligosaccharides"[MeSH Terms]) OR (oligosaccharide*[Title/Abstract])) OR (fructooligosaccharide*[Title/Abstract])) OR (fructo?oligosaccharide*[Title/Abstract])) OR (galactooligosaccharide*[Title/Abstract])) OR (galacto?oligosaccharide*[Title/Abstract])) OR (RP-G28[Title/Abstract])) OR (acidicoligosaccharide*[Title/Abstract])) OR (acidic?oligosaccharide*[Title/Abstract])) OR (fructooligosaccharide[Supplementary Concept])) OR (fructooligosaccharide*[Title/Abstract])) OR (fructo?oligosaccharide*[Title/Abstract])) OR (galactooligosaccharide*[Title/Abstract])) OR (galacto?oligosaccharide*[Title/Abstract])) OR (Soybeanoligosaccharide*[Title/Abstract])) OR (Soybean?oligosaccharide*[Title/Abstract])) OR (xyloseoligosaccharide*[Title/Abstract])) OR (xylose?oligosaccharide*[Title/Abstract])) OR (Lactosylfructoside*[Title/Abstract])) OR (Lactosyl?fructoside*[Title/Abstract])) OR (Isomaltooligosaccharide*[Title/Abstract])) OR (Isomalto?oligosaccharide*[Title/Abstract])) OR**

(Gentiooligsaccharide*[Title/Abstract])) OR (Gentio?oligsaccharide*[Title/Abstract])) OR (Mannose?oligosaccharides[Title/Abstract])) OR (Mannoseoligosaccharides*[Title/Abstract])

45) (((("Lactulose"[MeSH Terms]) OR (Lactulose*[Title/Abstract])) OR (Duphalac[Title/Abstract])) OR (Normase[Title/Abstract])) OR (Amivalex[Title/Abstract])
46) ((((((isomaltulose[Supplementary Concept]) OR (isomaltulose[Title/Abstract])) OR (palatinose[Title/Abstract])) OR (isomaltulose anhydrous[Title/Abstract])) OR (6-O alpha-D-glucopyranosyl-D-fructose[Title/Abstract])) OR (isomaltulose monohydrate[Title/Abstract])) OR (palatinose monohydrate[Title/Abstract])) OR (D-fructose, 6-O-alpha-D-glucopyranosyl-, monohydrate[Title/Abstract])
47) (((stachyose[Supplementary Concept]) OR (stachyose[Title/Abstract])) OR (stachyose tetrahydrate[Title/Abstract])) OR (stachyose hydrate[Title/Abstract])
48) ((("Raffinose"[MeSH Terms]) OR (Melitose[Title/Abstract])) OR (Melitriose[Title/Abstract])) OR (Gossypose[Title/Abstract])
49) ("Trehalose"[MeSH Terms]) OR (Trehalose*[Title/Abstract])
50) ("inulin"[MeSH Terms]) OR (inulin[Title/Abstract])
51) ((((((((((((((("Enteral nutrition"[MeSH Terms]) OR (Enteral nutrition*[Title/Abstract])) OR (Nutrition, Enteral[Title/Abstract])) OR (Enteral Feeding[Title/Abstract])) OR (Feeding, Enteral[Title/Abstract])) OR (Force Feeding*[Title/Abstract])) OR (Feeding, Force[Title/Abstract])) OR (Feedings, Force[Title/Abstract])) OR (Tube Feeding[Title/Abstract])) OR (Feeding, Tube[Title/Abstract])) OR (Feeding Tube, Gastric[Title/Abstract])) OR (Feeding Tubes, Gastric[Title/Abstract])) OR (Gastric Feeding Tube*[Title/Abstract])) OR (Tube, Gastric Feeding[Title/Abstract])) OR (Tubes, Gastric Feeding[Title/Abstract])) OR (EN[Title/Abstract])) OR (EPN[Title/Abstract])
52) (((((((("Parenteral Nutrition, Total"[MeSH Terms]) OR (Parenteral Nutrition, Total[Title/Abstract])) OR (Parenteral Hyperalimentation[Title/Abstract])) OR (Total Parenteral Nutrition*[Title/Abstract])) OR (Hyperalimentation, Parenteral[Title/Abstract])) OR (Nutrition, Total Parenteral[Title/Abstract])) OR (Intravenous Hyperalimentation[Title/Abstract])) OR (Hyperalimentation, Intravenous[Title/Abstract])) OR (TPN[Title/Abstract])
53) ((((((((("Parenteral Nutrition"[MeSH Terms]) OR (Parenteral Nutrition*[Title/Abstract])) OR (Nutrition, Parenteral[Title/Abstract])) OR (Parenteral Feeding*[Title/Abstract])) OR (Feeding, Parenteral[Title/Abstract])) OR (Feedings, Parenteral[Title/Abstract])) OR (Intravenous Feeding*[Title/Abstract])) OR (Feeding, Intravenous[Title/Abstract])) OR (Feedings, Intravenous[Title/Abstract])) OR (PN[Title/Abstract])
54) (((("Parenteral Nutrition Solutions"[MeSH Terms]) OR (Parenteral Nutrition Solutions[Title/Abstract])) OR (Intravenous Feeding Solutions[Title/Abstract])) OR (Feeding Solutions, Intravenous[Title/Abstract])) OR (Solutions, Intravenous Feeding[Title/Abstract])
55) Or/23-54
56) ((("Pneumonia, Ventilator-Associated"[MeSH Terms]) OR (Pneumonia, Ventilator-Associated[Title/Abstract])) OR (Ventilator-Associated

Pneumonia[Title/Abstract])) OR (VAP[Title/Abstract])

57) (((((((((((((((((("Pneumonia"[MeSH Terms]) OR (pneumon*[Title/Abstract])) OR (pleuropneumon*[Title/Abstract])) OR (bronchopneumon*[Title/Abstract])) OR (bronchit*[Title/Abstract])) OR (tracheobronchit*[Title/Abstract])) OR (Lobar Pneumonia*[Title/Abstract])) OR (Pneumonias, Lobar[Title/Abstract])) OR (Pneumonia, Lobar[Title/Abstract])) OR (Experimental lung inflammation*[Title/Abstract])) OR (inflammation, Experimental lung[Title/Abstract])) OR (lung inflammation, Experimental[Title/Abstract])) OR (lung inflammations, Experimental[Title/Abstract])) OR (Pulmonary inflammation*[Title/Abstract])) OR (inflammation, Pulmonary[Title/Abstract])) OR (inflammations, Pulmonary[Title/Abstract])) OR (lung inflammation*[Title/Abstract])) OR (inflammation, lung[Title/Abstract])) OR (inflammations, lung[Title/Abstract])

58) ((((((((((((("Cross Infection"[MeSH Terms]) OR (Cross Infection*[Title/Abstract])) OR (Infection, Cross[Title/Abstract])) OR (Infections, Cross[Title/Abstract])) OR (Healthcare Associated Infection*[Title/Abstract])) OR (Infection, Healthcare Associated[Title/Abstract])) OR (Infections, Healthcare Associated[Title/Abstract])) OR (Health Care Associated Infection*[Title/Abstract])) OR (Hospital Infection*[Title/Abstract])) OR (Infections, Hospital[Title/Abstract])) OR (Infection, Hospital[Title/Abstract])) OR (Nosocomial Infection*[Title/Abstract])) OR (Infections, Nosocomial[Title/Abstract])) OR (Infection, Nosocomial[Title/Abstract])

59) ((("Pneumonia, Bacterial"[MeSH Terms]) OR (Pneumonia, Bacterial[Title/Abstract])) OR (Pneumonias, Bacterial[Title/Abstract])) OR (Bacterial Pneumonia*[Title/Abstract])

60) Or/56-59

61) 22 and 55 and 60

62) (((((((((((randomized controlled trial[Publication Type]) OR (controlled clinical trial[Publication Type])) OR (randomised[Title])) OR (randomized[Title])) OR (randomization[Title])) OR (randomisation[Title])) OR (randomisation[Title])) OR (placebo[Title])) OR (randomly[Title])) OR (trial[Title])) OR (groups[Title])) OR (drug therapy[MeSH Subheading])

63) ("animals"[MeSH Terms]) NOT ("humans"[MeSH Terms])

64) (((Editorial[Publication Type]) OR (Letter[Publication Type])) OR (Case Reports[Publication Type])) OR (Comment[Publication Type])

65) 62 not 63 not 64

66) 61 and 65
