# Supplementary material for: Probiotic Supplementation Prevents the Development of Ventilator-Associated Pneumonia for Mechanically Ventilated ICU Patients: A Systematic Review and Network Meta-analysis of Randomized Controlled Trials
Source: Front Nutr. 2022 Jul 8;9:919156. doi: 10.3389/fnut.2022.919156 (PMC9307490; doi:10.3389/fnut.2022.919156)
Supplement: Supplementary File 2 — Diagnostic criteria for outcomes.pdf. [file Data_Sheet_2.PDF]

Supplementary 2

Diagnostic criteria for outcomes

Table S 2.1 Diagnostic criteria for ventilator-associated Pneumonia in studies included in network meta-analysis

ID	Study	Ventilator-associated Pneumonia
1	Caparros, 2001	The CDC criteria: the chest radiographic examination showed new or progressive infiltrate, consolidation, or cavitation and at least 2 of the following were present: temperature above 38.5 ℃ or below 35 ℃; a white blood cell count greater than 10×10^9/L or $<3\times10^9$/L; isolation of pathogens from the sputum or bronchial aspirates or bronchial brushing; isolation of a pathogen from blood cultures; or diagnostic single antibody titer (IgM) or 4-fold increase in paired serum L samples (IgG) for pathogens.
6	Kotzampassi, 2006	The CDC criteria: these criteria require there to be at least one positive sample (protected specimen brush or plugged telescoping catheter for bronchoalveolar minilavage [$>10^3$ colony-forming units (CFUs)/ml] or endotracheal aspirate with [$>10^5$ CFUs/ml and >25 leucocytes/high-power field]); also required is the presence of one or several new abnormal radiographical and progressive parenchymatous infiltrates and one of the following signs: purulent sputum production, fever (temperature > 38.5 ℃), pathogenic bacteria in blood culture without other infection source, and bronchoalveolar minilavage with more than 5% cells with intethnicityllular bacteria.
7	Giamarellos-Bourboulis, 2009	VAP was diagnosed in patients presenting with all of the following: (a) new or persistent consolidation in lung X-ray, (b) purulent tracheobroncial secretions(Cultures of tracheobroncial secretions yielding a pathogen at a count $\geq10^6$ CFU/mL), and (c) CPIS more than 6.
8	Knight, 2009	There were new progressive, or persistent (＞24 h) infiltration on chest radiograph plus at least two of the following: (1)Temperature ＞38.0℃, (2) Leucocytosis (WBC count＞ 12×10^3uL^{-1}) or leucopoenia (WBC count＜4 $\times10^3$uL^{-1}), (3) Purulent tracheobronchial secretions.
9	Moses, 2009	The appearance of a new or progressive pulmonary infiltrate and any two of the following: (a) temperature >38.0 ℃ or <36.0 ℃, (b) WBC count >10,000 or <4,000/uL, (c) purulent tracheobronchial secretions. Quantitative endotracheal aspirates with cultures were performed in patients suspected to have VAP, with $\geq 10^5$ colony-forming units taken as significant
10	Barraud, 2010	VAP was defined by the presence of (a) a new and persistent infiltrate on chest radiograph associated with at least one of the following: purulent tracheal secretions, temperature 38.3 ℃ or higher, and a leukocyte count of 10,000 uL^{-1} or higher; and (b) positive quantitative cultures of distal pulmonary secretions obtained from bronchoalveolar lavage (significant threshold more than 10^4colonyforming units/mL).
12	Morrow, 2010	The ACCP clinical criteria require a new and persistent infiltrate on chest radiographs with two of three supporting findings: fever (＞38.5 ℃ or ＜35.0 ℃), leukocytosis (white blood cells＞10,000/mm^3 or＜3,000/mm^3), and purulent sputum.
13	Altintas, 2011	VAP was defined as the presence of otherwise unexplained new and persistent or progressive infiltration on chest x-ray after >48 hours of mechanical ventilation and the presence of 2 of the following: >38 ℃ fever with no other explanation, leukopenia (<4,000/mL)/leukocytosis (≥12,000/mL), new-onset purulent airway secretions, increase in the amount of oxygen needed, and deterioration of the PaO_2 /FiO_2 ratio(<250). Endotracheal aspirate cultures were used to confirm VAP.
14	Tan, 2011	The ATS/IDSA criteria:As pneumonia occurring more than 48 hours after endotracheal intubation, and was diagnosed by the presence of both a new or progressive radiographic infiltrate plus at least two clinical features - fever > 38.0 ℃, leucocytosis (white blood cells count > 12×10^9/l), leucopenia (white blood cells count < 4×10^9/l), or purulent tracheobronchial secretions -and positive semiquantitative cultures of tracheobronchial secretions.
15	Aydoğmuş, 2012	There were new progressive, or persistent (＞24 h) infiltration on chest radiograph plus at least two of the following: (1)Temperature ＞38.0℃, (2) Leucocytosis (WBC count＞ 10×10^3uL^{-1}) or leucopoenia (WBC count＜4 $\times10^3$uL^{-1}), (3) Purulent tracheobronchial secretions.
16	Rongrungruang, 2015	The ATS/IDSA criteria:A diagnosis of VAP was made if the patient had a new, persistent, or progressive infiltrate visible on a chest radiograph in combination with at least 3 of the following 4 criteria: 1) body temperature greater than 38 ℃ or less than 35.5 ℃, 2) leukocytosis (>10,000 leukocytes/mm^3) or leukopenia (<3,000 leukocytes/mm^3), 3) purulent tracheal aspirate, and 4) a semi-quantitative culture of tracheal aspirate samples that was positive for pathogenic bacteria.
18	Zarinfar N, 2016	Clinical manifestations plus laboratory test results.
19	Zeng, 2016	The ACCP clinical criteria: VAP was based on the presence of a new, persistent or progressive infiltrate on chest radiographs that persisted for at least 48 h combined with at least two of the following criteria: (1) a temperature of >38.0 ℃ or <35.5 ℃; (2) a blood leukocytosis count of $>12\times10^3$/mm^3 or $<3\times10^3$/mm^3 and/or left shift; (3)purulent tracheal aspirates.
20	Fazilaty, 2018	The ATS/IDSA criteria:A new infiltrate on chest X-rays occurring more than 48 hours after endotracheal intubation plus two or more of the following: fever (body temperate >38.3 ℃), leukocytosis (white blood cell count $>12\times10^9$/ml), leucopenia (white blood cell count $<4\times10^9$/ml), and purulent tracheobronchial secretions.
21	Kooshk, 2018	The ACCP clinical criteria: VAP was based on the presence of a new, persistent or progressive infiltrate on chest radiographs that persisted for at least 48 h combined with at least two of the following criteria: (1) a temperature of >38.0 ℃ or <35.5 ℃; (2) a blood leukocytosis count of $>12\times10^3$/mm^3 or $<3\times10^3$/mm^3 and/or left shift; (3)purulent tracheal aspirates.

Table S 2.1 Diagnostic criteria for ventilator-associated Pneumonia in studies included in network meta-analysis (Continued)

ID	Study	Ventilator-associated Pneumonia
22	Reiginer,2018	New and persistent or progressive lung infiltrates on the chest radiograph, combined with at least two of the following criteria: body temperature ≥38.5 ℃ or ≤35.5 ℃, peripheral leukocytosis (>10,000/mm^3) or leukopenia (<4,000/mm^3), and purulent tracheal aspirates. The diagnosis must be confirmed in each participating ICU on the basis of a positive semiquantitative bacteriological result from a distal respiratory specimen: bronchoalveolar lavage fluid (positive if there are ≥10^4 colony-forming units (cfu)/ml), protected specimen brush (positive if there are ≥10^3 cfu/ml) or tracheobronchial aspirate (positive if there are ≥10^5 cfu/ml).
23	Shimizu, 2018	The ATS/IDSA criteria:A new infiltrate on chest X-rays occurring more than 48 hours after endotracheal intubation plus two or more of the following: fever (body temperate >38.3 ℃), leukocytosis (white blood cell count >12×10^9/ml), leucopenia (white blood cell count <4×10^9/ml), and purulent tracheobronchial secretions.
24	Mahmoodpoor, 2019	The ACCP clinical criteria: VAP was based on the presence of a new, persistent or progressive infiltrate on chest radiographs that persisted for at least 48 h combined with at least two of the following criteria: (1) a temperature of >38.0 ℃ or <35.5 ℃; (2) a blood leukocytosis count of >12 × 10^3/mm^3 or <3 × 10^3/mm^3 and/or left shift; (3)leukocytosis or leucopenia, or purulent sputum underwent bronchoalveolar lavage (BAL). patients were considered VAP-positive if the quantitative BAL culture had at least 10^4 colony-forming units/mL in patients who were mechanically ventilated for >48 hours.
25	Anandaraj, 2019	The appearance of a new or progressive pulmonary infiltrate and any two of the following: (a) temperature >38.0 ℃ or <36.0 ℃, (b) WBC count >10,000 or <4,000/uL, (c) purulent tracheobronchial secretions. Quantitative endotracheal aspirates with cultures were performed in patients suspected to have VAP, with ≥ 10^5 colony-forming units taken as significant.
26	Jin, 2019	VAP was based on the presence of a new, persistent or progressive infiltrate on chest radiographs that persisted for at least 48 h combined with at least two of the following criteria: (1) a temperature of >38.0 ℃ or <36.0 ℃; (2) a blood leukocytosis count of >12 × 10^3/mm^3 or <4 × 10^3/mm^3 and/or left shift; (3)purulent tracheal aspirates.
27	Nseir, 2019	New and persistent or progressive lung infiltrates on the chest radiograph, combined with at least two of the following criteria: body temperature ≥38.5 ℃ or ≤35.5 ℃, peripheral leukocytosis (>10,000/mm^3) or leukopenia (<4,000/mm^3), and purulent tracheal aspirates. The diagnosis must be confirmed in each participating ICU on the basis of a positive semiquantitative bacteriological result from a distal respiratory specimen: bronchoalveolar lavage fluid (positive if there are ≥10^4 colony-forming units (cfu)/ml), protected specimen brush (positive if there are ≥10^3 cfu/ml) or tracheobronchial aspirate (positive if there are ≥10^5 cfu/ml)
28	Habib, 2020	Using clinical, invasive diagnostic strategy, and surveillance for VAP.
29	Nazari, 2020	VAP was based on the presence of a new, persistent or progressive infiltrate on chest radiographs that persisted for at least 48 h combined with the following criteria: fever; a blood leukocytosis with a left shift along with another synptom including increase in the pulmonary, foul-smelling pulmonary secretions; increased need for respiratory support without systemic problems or metabolic disorders.
30	Johnstone, 2021	The ACCP clinical criteria: VAP informed by the presence of a new,progressive,or persistent radiographic infiltrate on chest radiograph after at least 2 days of mechanical ventilation,plus any 2 of the following: (1) fever (core temperature >38 ℃) or hypothermia(temperature<36 ℃);(2)white blood cell count less than 3.0 × 10^6/L or exceeding 10 × 10^6/L, and (3) purulent sputum.
31	Maria, 2021	VAP was defined as any episode of lower respiratory tract infection that met all the following criteria: (a) onset > 48 h after initiation of mechanical ventilation; (b) increase in SOFA score by ≥1 point; (c) new infiltrate in chest X-ray or chest computed tomography; (d) core temperature > 38 ℃; (e) purulent tracheobronchial secretions; (f) clinical pulmonary infection score > 6; and (g) isolation of a pathogen from BAL fluid at > 1 × 10^4 cfu/mL.

Abbreviation: ACCP: American College of Chest Physicians; ATS:American Thoracic Society; CDC: Centers for Disease Control and Prevention; CFUs: Colony forming units; CPIS: clinical pulmonary infection score; IDSA:Infectious Diseases Society of America; LCBI:Laboratory-confirmed bloodstream infection;

Table S 2.2 Diagnostic criteria for bloodstream infection in studies included in network meta-analysis

ID	Study	Bloodstream infection
1	Caparros, 2001	A pathogen was isolated from the blood of a patient with a temperature above 38.5 ℃ or below 35 ℃, or with a white blood cell count greater than 10 × 10^9/L or <3 × 10^9/L, and the pathogen was not related to infection at another site.
3	Radrizzani, 2006	The CDC criteria: LCBI must meet at least 1 of the following criteria: 1.Patient has a recognized pathogen cultured from 1 or more blood cultures AND organism cultured from blood is not related to an infection at another site. 2. Patient has at least 1 of the following signs or symptoms: fever (>38 ℃), chills, or hypotension AND signs and symptoms and positive laboratory results are not related to an infection at another site AND common skin contaminant (ie, diphtheroids [Corynebacterium spp], Bacillus [not B anthracis] spp, Propionibacterium spp, coagulase-negative staphylococci [including S epidermidis], viridans group streptococci, Aerococcus spp, Micrococcus spp) is cultured from 2 or more blood cultures drawn on separate occasions.
4	Spindler-Vesel,2006	Positive blood culture with clinical significance.
7	Giamarellos-Bourboulis, 2009	Positive blood culture with clinical significance. Primary bacteremia was defined as any case of bacteremia yielding an isolate that was not isolated from any other source.
14	Tan, 2011	The CDC criteria: LCBI must meet at least 1 of the following criteria: 1.Patient has a recognized pathogen cultured from 1 or more blood cultures AND organism cultured from blood is not related to an infection at another site. 2. Patient has at least 1 of the following signs or symptoms: fever (>38 ℃), chills, or hypotension AND signs and symptoms and positive laboratory results are not related to an infection at another site AND common skin contaminant (ie, diphtheroids [Corynebacterium spp], Bacillus [not B anthracis] spp, Propionibacterium spp, coagulase-negative staphylococci [including S epidermidis], viridans group streptococci, Aerococcus spp, Micrococcus spp) is cultured from 2 or more blood cultures drawn on separate occasions.
22	Reiginer,2018	Positive blood culture with clinical significance.
23	Shimizu, 2018	Bacteremia was defined as a positive blood culture after the first 3 days.
30	Johnstone, 2021	Patient must meet the following two criteria: Patient has a recognized pathogen (defined as a microorganism not usually regarded as a common skin contaminant, i.e., diphtheroids, Bacillus species, Propionibacterium species, coagulase-negative staphylococci, or micrococci) cultured from one or more blood cultures OR a common skin contaminant (e.g., diphtheroids, Bacillus species, Propionibacterium species, coagulase-negative staphylococci, or micrococci) cultured from two or more blood cultures drawn on separate occasions (including one drawn by venipuncture) AND the organism cultured from blood is not related to an infection at another site, including intravascular-access devices
31	Maria, 2021	The isolation of one Gram-positive or Gram-negative pathogen in peripheral blood culture, not meeting the definition of central catheter-associated infection

Abbreviation: CDC: Centers for Disease Control and Prevention; LCBI:Laboratory-confirmed bloodstream infection;

Table S 2.3 Diagnostic criteria for urinary tract infection in studies included in network meta-analysis

ID	Study	Urinary tract infection
1	Caparros 2001	The CDC criteria:A symptomatic urinary tract infection must meet at least 1 of the following criteria: 1.Patient has at least 1 of the following signs or symptoms with no other recognized cause: fever (>38 ℃), urgency, frequency, dysuria, or suprapubic tenderness AND patient has a positive urine culture, that is, ≥ 10^5 microorganisms per cc of urine with no more than 2 species of microorganisms. 2. Patient has at least 2 of the following signs or symptoms with no other recognized cause: fever(>38 ℃), urgency, frequency, dysuria, or suprapubic tenderness AND at least 1 of the following: a. positive dipstick for leukocyte esterase and/or nitrate; b. pyuria (urine specimen with ≥10 white blood cell [WBC]/mm^3 or ≥3 WBC/high-power field of unspun urine); c. organisms seen on Gram's stain of unspun urine; d. at least 2 urine cultures with repeated isolation of the same uropathogen (gram-negative bacteria or Staphylococcus saprophyticus) with ≥ 10^2 colonies/mL in nonvoided specimens e. ≤10^5 colonies/mL of a single uropathogen (gram-negative bacteria or S saprophyticus) in a patient being treated with an effective antimicrobial agent for a urinary tract infection; f. physician diagnosis of a urinary tract infection; g. physician institutes appropriate therapy for a urinary tract infection.
2	Kotzampassi, 2006	The urine culture showed at least 10^5 colonies of a pathogen.
3	Radrizzani,2006	The CDC criteria:A symptomatic urinary tract infection must meet at least 1 of the following criteria: 1.Patient has at least 1 of the following signs or symptoms with no other recognized cause: fever (>38 ℃), urgency, frequency, dysuria, or suprapubic tenderness AND patient has a positive urine culture, that is, ≥ 10^5 microorganisms per cc of urine with no more than 2 species of microorganisms. 2. Patient has at least 2 of the following signs or symptoms with no other recognized cause: fever(>38 ℃), urgency, frequency, dysuria, or suprapubic tenderness AND at least 1 of the following: a. positive dipstick for leukocyte esterase and/or nitrate; b. pyuria (urine specimen with ≥10 white blood cell [WBC]/mm^3 or ≥3 WBC/high-power field of unspun urine); c. organisms seen on Gram's stain of unspun urine; d. at least 2 urine cultures with repeated isolation of the same uropathogen (gram-negative bacteria or Staphylococcus saprophyticus) with ≥ 10^2 colonies/mL in nonvoided specimens e. ≤10^5 colonies/mL of a single uropathogen (gram-negative bacteria or S saprophyticus) in a patient being treated with an effective antimicrobial agent for a urinary tract infection; f. physician diagnosis of a urinary tract infection; g. physician institutes appropriate therapy for a urinary tract infection.
4	Spindler-Vesel, 2007	The CDC criteria:A symptomatic urinary tract infection must meet at least 1 of the following criteria: 1.Patient has at least 1 of the following signs or symptoms with no other recognized cause: fever (>38 ℃), urgency, frequency, dysuria, or suprapubic tenderness AND patient has a positive urine culture, that is, ≥ 10^5 microorganisms per cc of urine with no more than 2 species of microorganisms. 2. Patient has at least 2 of the following signs or symptoms with no other recognized cause: fever(>38 ℃), urgency, frequency, dysuria, or suprapubic tenderness AND at least 1 of the following: a. positive dipstick for leukocyte esterase and/or nitrate; b. pyuria (urine specimen with ≥10 white blood cell [WBC]/mm^3 or ≥3 WBC/high-power field of unspun urine); c. organisms seen on Gram's stain of unspun urine; d. at least 2 urine cultures with repeated isolation of the same uropathogen (gram-negative bacteria or Staphylococcus saprophyticus) with ≥ 10^2 colonies/mL in nonvoided specimens e. ≤10^5 colonies/mL of a single uropathogen (gram-negative bacteria or S saprophyticus) in a patient being treated with an effective antimicrobial agent for a urinary tract infection; f. physician diagnosis of a urinary tract infection; g. physician institutes appropriate therapy for a urinary tract infection.
7	Giamarellos-Bourboulis,2009	Urine samples yielding a pathogen at a concentration ≥10^5 CFUs /mL were considered positive.
9	Moses, 2009	≥10^5 CFUs/mL of one or two organisms first identified after at least 48 h of stay in ICU
10	Barraud, 2010	1. Lower urinary tract infection:The presence of suggestive signs and symptoms including fever (>38 ℃), urgency, frequency, dysuria, pyuria, hematuria, positive Gram stain, pus, suggestive imaging AND Positive dipstick for leukocyte esterase and/or nitrate or pyuria (≥10 white blood cells/uL or ≥3 white blood cells/high-power field of unspun urine) or organisms seen on Gram stain of unspun urine or frank pus expressed around the urinary catheter or >10^3 CFUs/mL or if the patient can report symptoms, modified CDC criteria have to be met; 2. Upper urinary tract infection (kidney, ureter, bladder, urethra, or tissue surrounding the retroperitoneal or perinephric space) Must meet one of the following criteria: Organism isolated from culture of fluid (other than urine) or tissue from the affected site; an abscess or other evidence of infection seen on direct examination, during surgery, or by histopathologic examination or two of the following: Fever (>38 ℃), urgency, localized pain or tenderness at involved site, and any of the following: purulent drainage from the affected site, pyuria, hematuria, organism isolated from culture, positive Gram stain, radiographic evidence of infection (e.g., ultrasound, computed tomography, magnetic resonance imaging, radiolabeled scan) Modified CDC criteria (1) 1) One of the following: fever (>38 ℃), urgency, frequency, dysuria or suprapubic tenderness, and a urine culture ≥10^5 CFUs/mL with no more than two species of organisms OR 2) Two of the following: fever (>38 ℃), urgency, frequency, dysuria or suprapubic tenderness, and any of the following: a) positive dipstick for leukocyte esterase and/or nitrate; b) pyuria (≥10 white blood cells/uL or ≥3 white blood cells/high-power field of unspun urine); c) organisms seen on Gram stain of unspun urine; d) two urine cultures with repeated isolation of the same uropathogen with ≥ 10^2 CFUs/mL in nonvoided specimen; e) two urine cultures with ≤10^5 CFUs/mL of single uropathogens in a patient being treated with appropriate antimicrobial therapy.

Table S 2.3 Diagnostic criteria for urinary tract infection in studies included in network meta-analysis (Continued)

ID	Study	Urinary tract infection
14	Tan, 2011	The CDC criteria:A symptomatic urinary tract infection must meet at least 1 of the following criteria: 1.Patient has at least 1 of the following signs or symptoms with no other recognized cause: fever (>38 ℃), urgency, frequency, dysuria, or suprapubic tenderness AND patient has a positive urine culture, that is, ≥ 10^5 microorganisms per cc of urine with no more than 2 species of microorganisms. 2. Patient has at least 2 of the following signs or symptoms with no other recognized cause: fever(>38 ℃), urgency, frequency, dysuria, or suprapubic tenderness AND at least 1 of the following: a. positive dipstick for leukocyte esterase and/or nitrate; b. pyuria (urine specimen with ≥10 white blood cell [WBC]/mm^3 or ≥3 WBC/high-power field of unspun urine); c. organisms seen on Gram's stain of unspun urine; d. at least 2 urine cultures with repeated isolation of the same uropathogen (gram-negative bacteria or Staphylococcus saprophyticus) with ≥ 10^2 colonies/mL in nonvoided specimens e. ≤10^5 colonies/mL of a single uropathogen (gram-negative bacteria or S saprophyticus) in a patient being treated with an effective antimicrobial agent for a urinary tract infection; f. physician diagnosis of a urinary tract infection; g. physician institutes appropriate therapy for a urinary tract infection.
20	Fazilaty, 2018	Clinical manifestations plus positive urine culture
22	Reiginer,2018	Clinical manifestations plus laboratory test results
30	Johnstone, 2021	Microbiologically confirmed abscess or other radiographic or surgical evidence of upper urinary tract infection with or without positive urine culture (positive urine culture alone not included).
31	Maria, 2021	The detection of one micro-organism at > 10^5 cfu/mL from quantitative urine culture accompanied by signs of infection that cannot be attributed to any other infection

Abbreviation: CDC: Centers for Disease Control and Prevention; CFUs: Colony forming units;

Table S 2.4 Diagnostic criteria for diarrhea in studies included in network meta-analysis

ID	Study	Diarrhea
2	Kotzampassi, 2006	No accurate definition is provided
8	Knight, 2009	No accurate definition is provided
9	Moses, 2009	No accurate definition is provided
10	Barraud, 2010	The occurrence of at least 3 liquid stools/day.
12	Morrow, 2010	Diarrhea: three or more loose stools per 24-h period or placement of a fecal management system for continuous liquid stool. ICU-associated diarrhea: Patients with diarrhea but three negative C. difficile cytotoxin assays.
13	Altintas, 2011	An increase in stool amount (>1 L) and frequency (>3 per day) with loss of stool consistency
16	Rongrungruang, 2015	No accurate definition is provided
18	Zarinfar N, 2016	Clostridium difficile-associated diarrhea
21	Kooshk, 2018	Clostridium difficile-associated diarrhea (three or more loose stools per 24 h period), ICU-associated diarrhea (presumablybecause of acute disorder, antibiotic administration and dietary changes).
22	Reiginer,2018	More than 300 ml of liquid stool or more than four loose stools per day
23	Shimizu, 2018	The acute onset of continuous liquid stools for more than 12 h
24	Mahmoodpoor, 2019	>3 times in a day, weight >250 g, volume >500 mL
26	Jin, 2019	No accurate definition is provided
30	Johnstone, 2021	World Health Organization definition (⩾3 loose or watery bowel movements per day).
31	Maria, 2021	No accurate definition is provided

Table S 2.5 Nosocomial infection in studies included in network meta-analysis

ID	Study	Nosocomial infection
1	Caparros,2001	Pneumonia, Bloodstream infection, Catheter-related bloodstream infection, Surgical infections,Urinary tract infection
2	Kotzampassi,2006	Bacterial pneumonia, Bacteremia, Urinary tract infection, Catheter-related sepsis,Wound infection
3	Radrizzani,2006	Pneumonia, Bacteremia, Urinary tract infection, Abdominal infection,Bone infection, Urinary tract infection, Lower respiratory tract infection, Pneumonia and Bacteremia
4	Spindler-Vesel,2006	Pneumonia, Bloodstream infection, Catheter-related bloodstream infection, Urinary tract infection
5	Abdulmeguid,2007	Pneumonia, intra-abdominal abscess, empyema, line sepsis, or fasciitis with wound dehiscence
9	Moses,2009	Ventilator-associated pneumonia, Catheter-related bloodstream infection, Urinary tract infection, Sepsis.
10	Barraud,2010	Ventilator-associated pneumonia, Bloodstream infection, Catheter-related bloodstream infection.
14	Tan,2011	Pneumonia,Ventilator-associated pneumonia, Bloodstream infection,Urinary tract infection, Wound infection, Sepsis
20	Fazilaty,2018	Ventilator-associated pneumonia, Catheter-related bloodstream infection, Urinary tract infection, Sepsis,Wound infection
22	Reiginer,2018	Ventilator-associated pneumonia, Bloodstream infection, Catheter-related bloodstream infection, Urinary tract infection, Soft-tissue infection,other infection
23	Shimizu,2018	Ventilator-associated pneumonia, Infection complication(bloodstream infection, enteritis), Enteritis
30	Johnstone,2021	Ventilator-associated pneumonia, Pneumonia,Iintra-abdominal abscess,Skin or soft-tissue infection, Clostridioides fifficile infection, Urinary tract infection, Meningitis,encephalitis,osteomyelitis,septicarthritis,sinusitis, mediastinitis.
