# Supplementary material for: Probiotic Supplementation Prevents the Development of Ventilator-Associated Pneumonia for Mechanically Ventilated ICU Patients: A Systematic Review and Network Meta-analysis of Randomized Controlled Trials
Source: Front Nutr. 2022 Jul 8;9:919156. doi: 10.3389/fnut.2022.919156 (PMC9307490; doi:10.3389/fnut.2022.919156)
Supplement: Supplementary File 3 — Results of meta-analysis of pairwise comparisons and network.pdf. [file Data_Sheet_3.PDF]

# Supplementary file 3
# Results from pairwise network meta-analysis and network network meta-analysis

Data are the OR or MD (95% CrI) in the column-defining treatment compared with the row-defining treatment. With treatment as the boundary, the lower left part of the table is the result of network network meta-analysis, and the upper right part of the table is the result of pairwise network meta-analysis. For network network meta-analysis, OR lower than 1 or MD lower than 0 favour the column-defining treatment. For pairwise network meta-analysis, OR higher than 1 or MD higher than 0 favour the row-defining treatment. To obtain OR or MD for comparisons in the opposite direction, reciprocals should be taken. Significant results are in bold and underscored. OR:odds ratio. MD:mean difference. CrI:credible interval. EPN: enteral nutrition and/or adjuvant peripheral parenteral nutrition. TPN: total parenteral nutrition.

**Table S 3.1 Pairwise and network estimated odds ratio (with 95% CrI) of interventions on nosocomial infection**

| | | | | |
|---|---|---|---|---|
| **Synbiotics** | -- | 6.60 (0.73, 60.49) | **5.20 (2.10, 14.1)** | -- |
| 0.24 (0.08, 0.83) | **Probiotics** | -- | 1.20 (0.66, 2.9) | -- |
| 0.16 (0.05, 0.52) | 0.65 (0.23, 1.90) | **Prebiotics** | 0.80 (0.38, 2.03) | -- |
| **0.19 (0.08, 0.47)** | 0.82 (0.34, 1.50) | 1.24 (0.49, 2.56) | **EPN** | 1.50 (0.81, 3.01) |
| 0.13 (0.04, 0.38) | 0.55 (0.17, 1.26) | 0.85 (0.25, 2.10) | 0.69 (0.33, 1.23) | **TPN** |

**Table S 3.2 Pairwise and network estimated odds ratio (with 95% CrI) of interventions on bloodstream infection**

| | | | | |
|---|---|---|---|---|
| **Synbiotics** | **--** | 1.8e+04(1.9e-05, 2.7e+13) | 2.10 (0.40, 12.39) | **-** |
| 0.50 (0.11, 3.47) | **Probiotics** | **--** | 1.20 (0.41, 10.52) | **--** |
| 0.27 (0.04, 1.38) | 0.53 (0.05, 2.50) | **Prebiotics** | 0.68 (0.11, 3.90) | **--** |
| 0.43 (0.11, 1.39) | 0.87 (0.19, 2.08) | 1.60 (0.43, 7.03) | **EPN** | 1.50 (0.30, 10.54) |
| 0.28 (0.04, 1.47) | 0.57 (0.06, 2.36) | 1.03 (0.16, 7.22) | 0.66 (0.16, 2.21) | **TPN** |

**Table S 3.3 Pairwise and network estimated odds ratio (with 95% CrI) of interventions on urinary tract infections**

| | | | | |
|---|---|---|---|---|
| **Synbiotics** | **--** | 1.8e-06(1.0e-38, 1.8e+25) | 3.20 (0.22, 100.05) | **--** |
| 0.42 (0.04, 5.24) | **Probiotics** | **--** | 1.40 (0.21, 26.77) | **--** |
| 0.41 (0.04, 11.9) | 0.95 (0.11, 22.81) | **Prebiotics** | 1.20 (0.14, 420.12) | **--** |
| 0.31 (0.04, 1.81) | 0.75 (0.12, 3.00) | 0.76 (0.04, 3.82) | **EPN** | 1.70 (0.23, 83.27) |
| 0.20 (0.01, 1.80) | 0.47 (0.02, 3.34) | 0.47 (0.01, 4.06) | 0.63 (0.07, 2.83) | **TPN** |

**Table S 3.4 Pairwise and network estimated odds ratio (with 95% CrI) of interventions on diarrhea**

| | | | | |
|---|---|---|---|---|
| **Synbiotics** | **--** | **--** | 2.80 (0.80, 11.27) | **--** |
| 0.60 (0.15, 3.13) | **Probiotics** | **--** | 1.70 (0.89, 4.50) | **--** |
| 7.87 (0.37, 370.18) | 12.53 (0.66, 490.91) | **Prebiotics** | **20.79 (1.40, 980.73)** | **--** |
| 0.36 (0.09, 1.25) | 0.60 (0.22, 1.13) | **0.05 (0, 0.71)** | **EPN** | 0.75 (0.14, 3.10) |
| 0.50 (0.07, 3.77) | 0.84 (0.13, 4.09) | 0.06 (0, 1.51) | 1.35 (0.32, 6.89) | **TPN** |

**Table S 3.5 Pairwise and network estimated odds ratio (with 95% CrI) of interventions on hospital mortality**

| | | | | |
|---|---|---|---|---|
| **Synbiotics** | **--** | **--** | 1.60 (0.96, 2.90) | **--** |
| 0.81 (0.53, 1.23) | **Probiotics** | **--** | 1.10 (0.83, 1.60) | **--** |
| 1.22 (0.66, 2.30) | 1.50 (0.89, 2.61) | **Prebiotics** | 2.20 (1.10, 4.80) | **--** |
| 0.76 (0.52, 1.09) | 0.94 (0.76, 1.15) | 0.63 (0.37, 1.01) | **EPN** | 1.00 (0.75, 1.70) |
| 0.76 (0.46, 1.15) | 0.95 (0.64, 1.23) | 0.62 (0.34, 1.04) | 1.00 (0.74, 1.22) | **TPN** |

**Table S 3.6 Pairwise and network estimated odds ratio (with 95% CrI) of interventions on ICU mortality**

| | | | | |
|---|---|---|---|---|
| **Synbiotics** | -- | 0.92 (0.04, 21.77) | 1.50 (0.79, 2.80) | - |
| 0.69 (0.34, 1.35) | **Probiotics** | - | 1.00 (0.71, 1.30) | - |
| 0.96 (0.36, 2.59) | 1.41 (0.60, 3.33) | **Prebiotics** | 0.80 (0.07, 9.02) | -- |
| 0.69 (0.38, 1.26) | 1.00 (0.74, 1.41) | 0.71 (0.32, 1.59) | **EPN** | 0.98 (0.70, 1.70) |
| 0.69 (0.30, 1.37) | 1.01 (0.54, 1.63) | 0.72 (0.27, 1.66) | 1.02 (0.58, 1.44) | **TPN** |

**Table S 3.7 Pairwise and network estimated mean difference (with 95% CrI) of interventions on hospital lengtn of stay**

| | | | | |
|---|---|---|---|---|
| **Synbiotics** | -- | -- | -2.10 (-10.29, 6.20) | -- |
| 3.90 (-4.73, 12.61) | **Probiotics** | -- | 1.90 (-0.95, 4.70) | -- |
| 2.05 (-8.41, 11.79) | -1.87 (-8.82, 4.50) | **Prebiotics** | -0.01 (-6.30, 5.70) | -- |
| 2.05 (-6.14, 10.37) | -1.86 (-4.69, 0.96) | 0.03 (-5.67, 6.31) | **EPN** | 0.95 (-2.40, 4.30) |
| 1.12 (-7.83, 10.01) | -2.81 (-7.18, 1.63) | -0.89 (-7.59, 6.21) | -0.93 (-4.27, 2.46) | **TPN** |

**Table S 3.8 Pairwise and network estimated mean difference (with 95% CrI) of interventions on ICU length of stay**

| | | | | |
|---|---|---|---|---|
| **Synbiotics** | -- | 1.60 (-9.10, 12.74) | 3.80 (-1.40, 9.30) | -- |
| -3.43 (-9.20, 2.10) | **Probiotics** | -- | 0.58 (-1.90, 3.10) | -- |
| -1.86 (-8.92, 4.83) | 1.54 (-4.37, 7.67) | **Prebiotics** | 1.70 (-4.00, 7.50) | -- |
| -4.00 (-9.35, 0.90) | -0.59 (-3.07, 1.92) | -2.14 (-7.70, 3.32) | **EPN** | 1.70 (-2.04, 5.40) |
| -5.72 (-12.22, 0.30) | -2.29 (-6.72, 2.09) | -3.86 (-10.5, 2.72) | -1.7 (-5.49, 1.91) | **TPN** |

**Table S 3.9 Pairwise and network estimated mean difference (with 95% CrI) of interventions on the duration of mechanical ventilation**

| | | | | |
|---|---|---|---|---|
| **Synbiotics** | -- | -0.51 (-9.20, 8.10) | 2.80 (-1.80, 7.90) | -- |
| -1.27 (-6.76, 3.61) | **Probiotics** | -- | 1.50 (-0.93, 4.00) | -- |
| -1.17 (-6.69, 4.37) | 0.07 (-4.36, 5.03) | **Prebiotics** | 1.10 (-2.80, 5.40) | -- |
| -2.77 (-7.59, 1.48) | -1.48 (-3.97, 0.93) | -1.59 (-5.87, 2.14) | **EPN** | 1.10 (-3.30, 5.40) |
| -3.78 (-10.26, 2.16) | -2.55 (-7.35, 2.44) | -2.63 (-8.65, 3.03) | -1.05 (-5.23, 3.32) | **TPN** |
