# Supplementary material for: Probiotic Supplementation Prevents the Development of Ventilator-Associated Pneumonia for Mechanically Ventilated ICU Patients: A Systematic Review and Network Meta-analysis of Randomized Controlled Trials
Source: Front Nutr. 2022 Jul 8;9:919156. doi: 10.3389/fnut.2022.919156 (PMC9307490; doi:10.3389/fnut.2022.919156)

# Supplementary file 4

# Results from network network meta-analysis

The size of the nodes corresponds to the total number of participants that study the treatments. The (directly) comparable treatments are linked with a line. The thickness of the line corresponds to the standard error of trials that study this comparison. The colours of the line corresponds to the quality of trials that study this comparison. low risk of bias [green] ,moderate risk of bias [yellow]. EPN: enteral nutrition and/or adjuvant peripheral parenteral nutrition. TPN: total parenteral nutrition.

**Figure S 4.1 Network plot of all intervention comparisons for nosocomial infection**

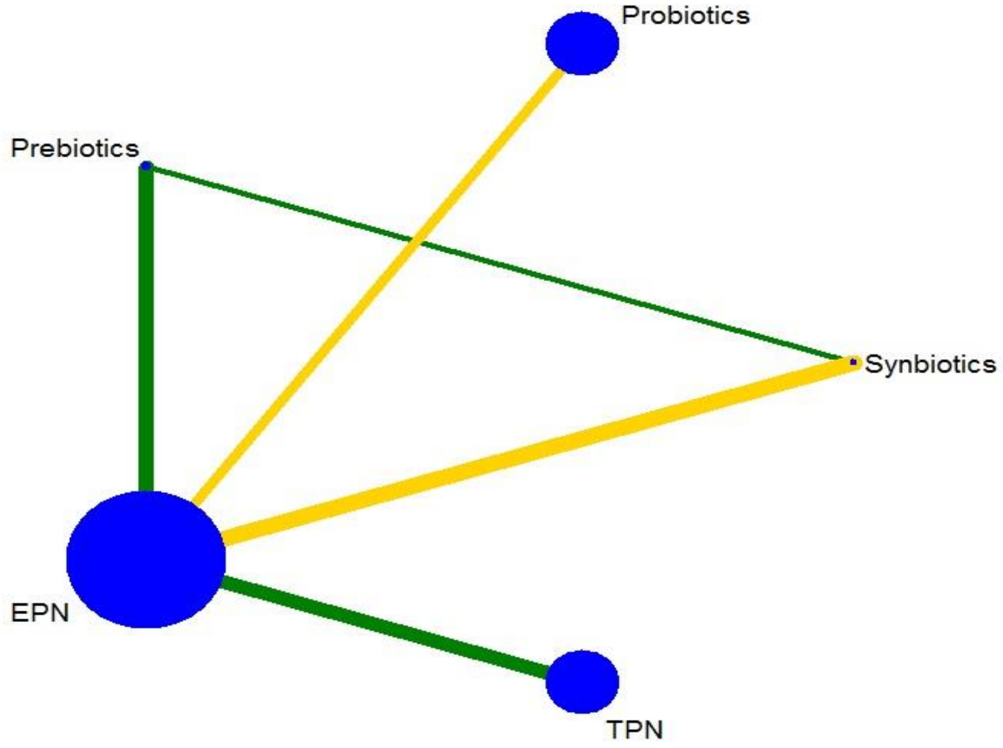

**Figure S 4.2 Network plot of all intervention comparisons for bloodstream infection**

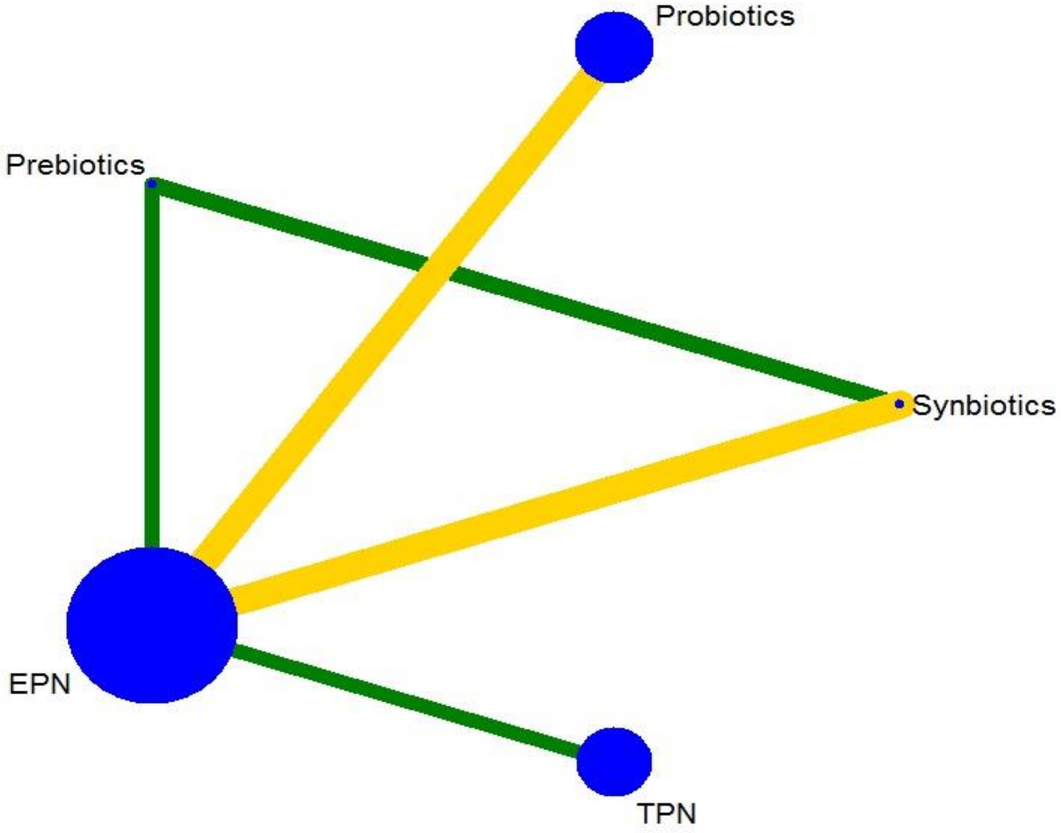


**Figure S 4.3 Network plot of all intervention comparisons for urinary tract infection**

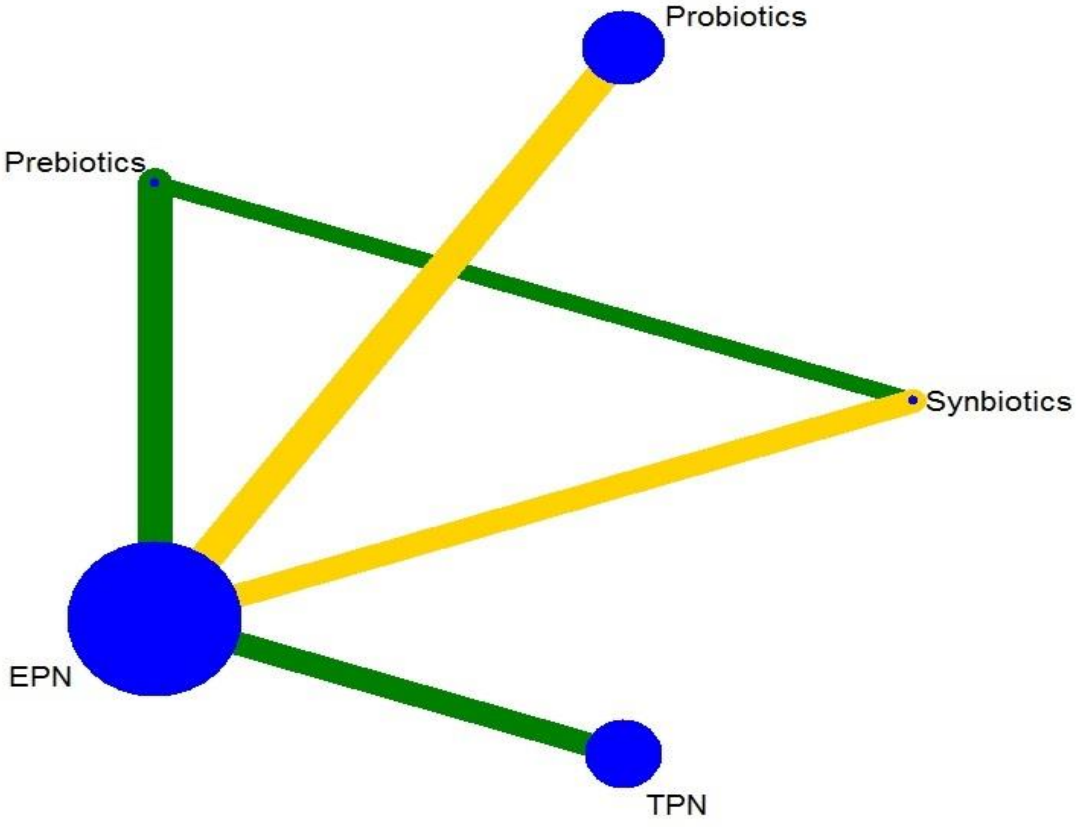

**Figure S 4.4 Network plot of all intervention comparisons for diarrhea**

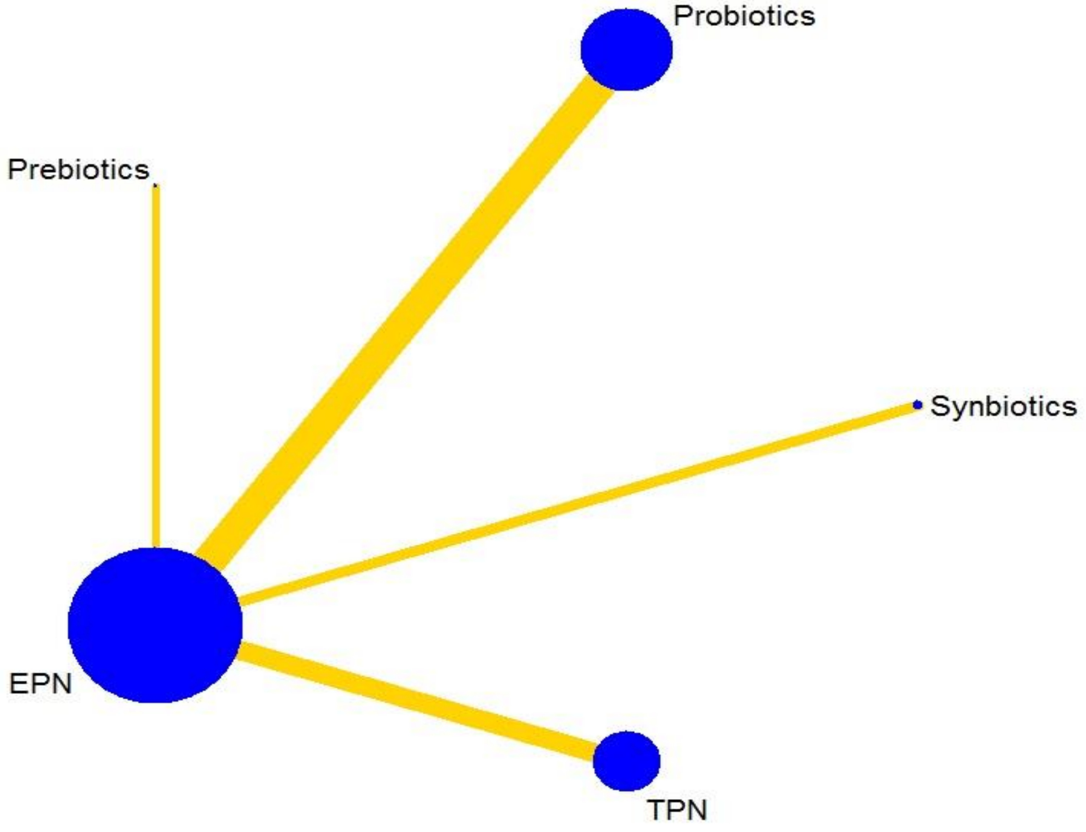


**Figure S 4.5 Network plot of all intervention comparisons for hospital mortality**

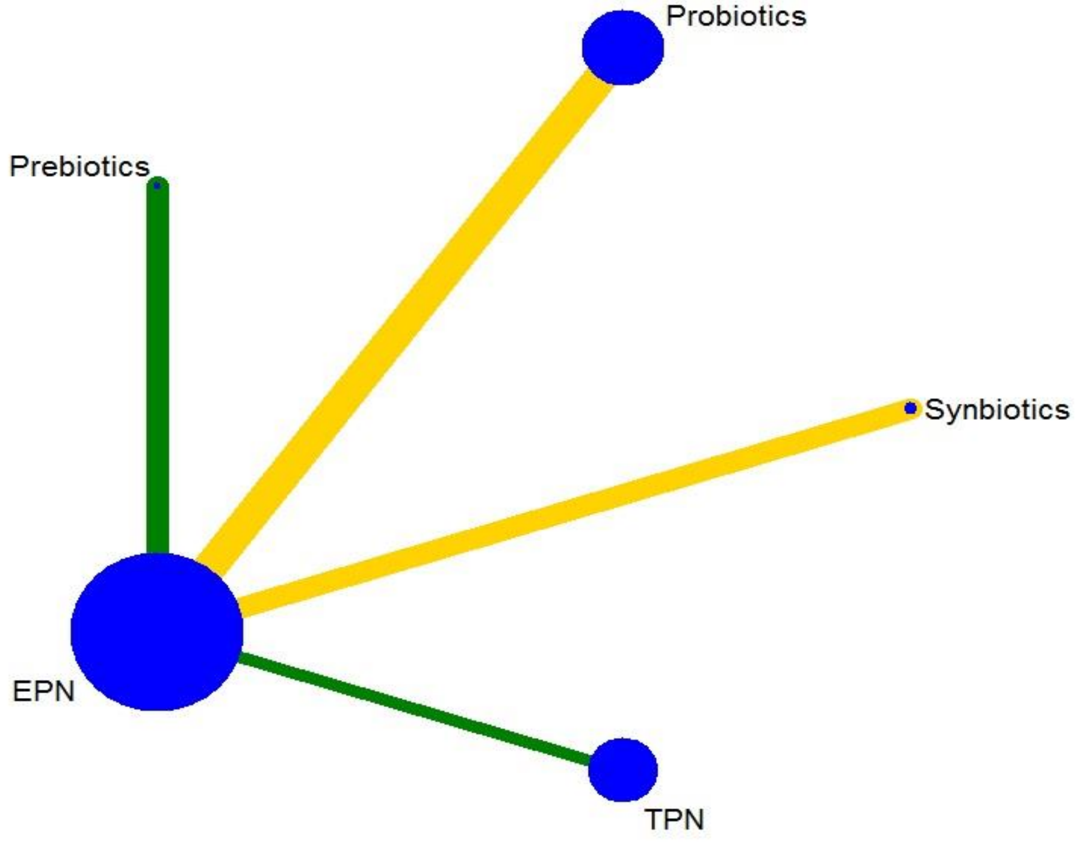

**Figure S 4.6 Network plot of all intervention comparisons for ICU mortality**

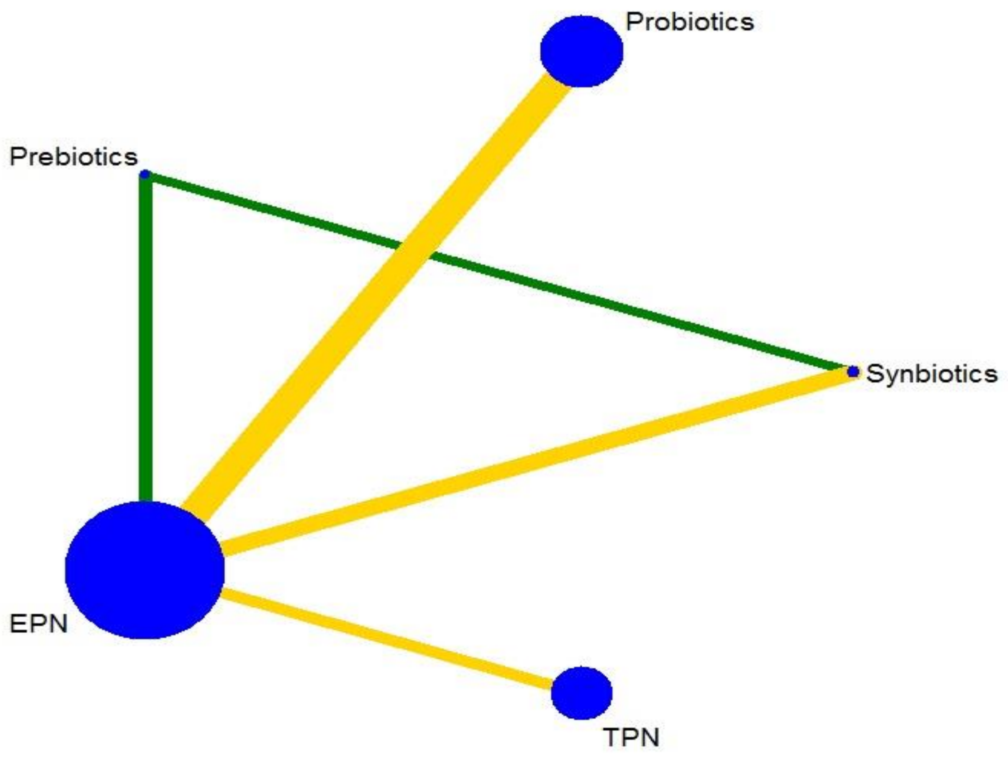


**Figure S 4.7 Network plot of all intervention comparisons for hospital lengtn of stay**

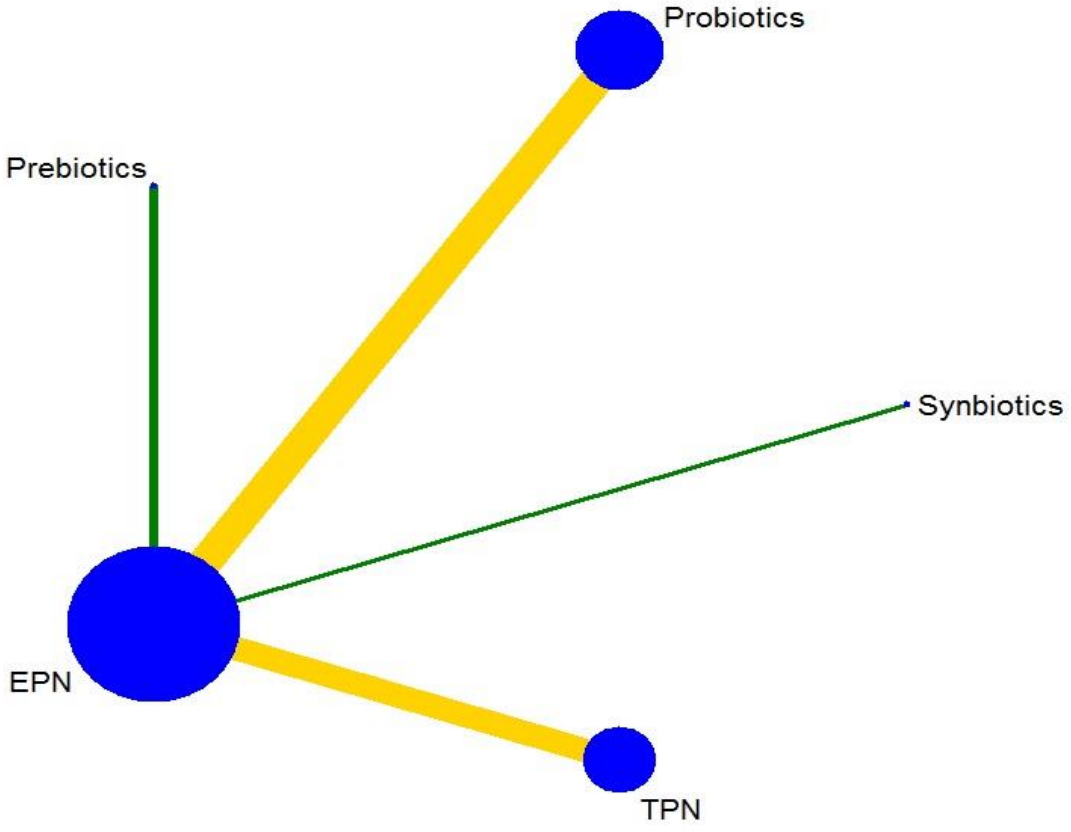

**Figure S 4.8 Network plot of all intervention comparisons for ICU length of stay**

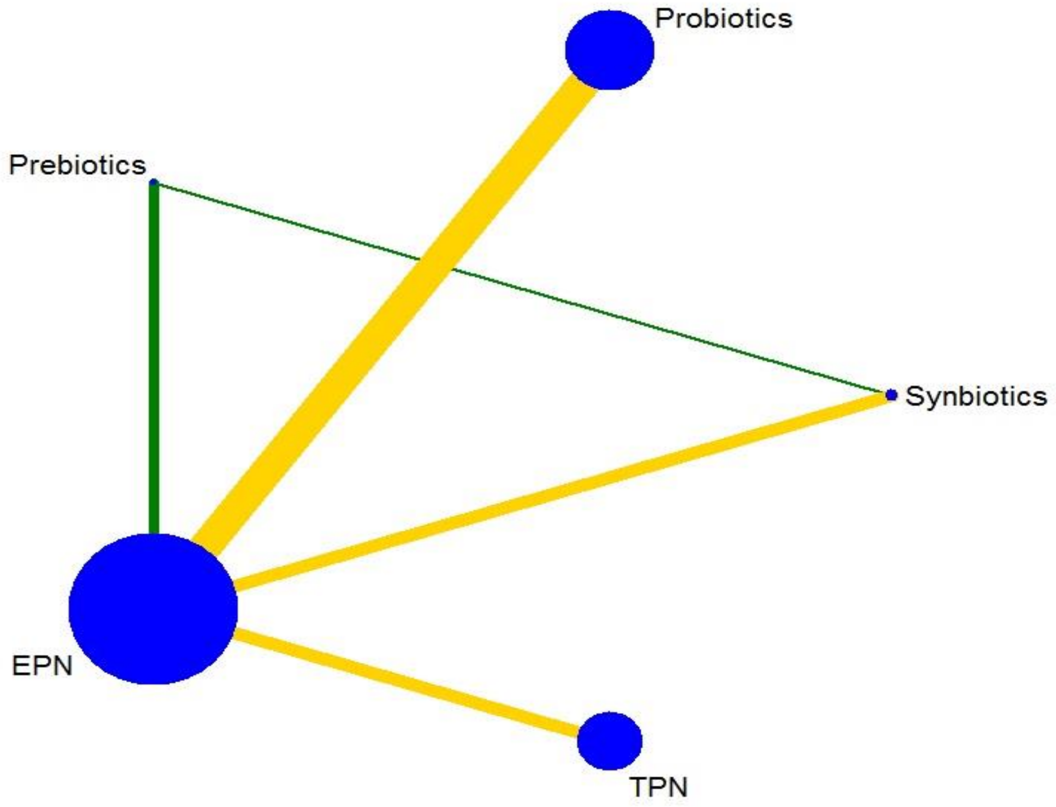


**Figure S 4.9 Network plot of all intervention comparisons for the duration of mechanical ventilation**

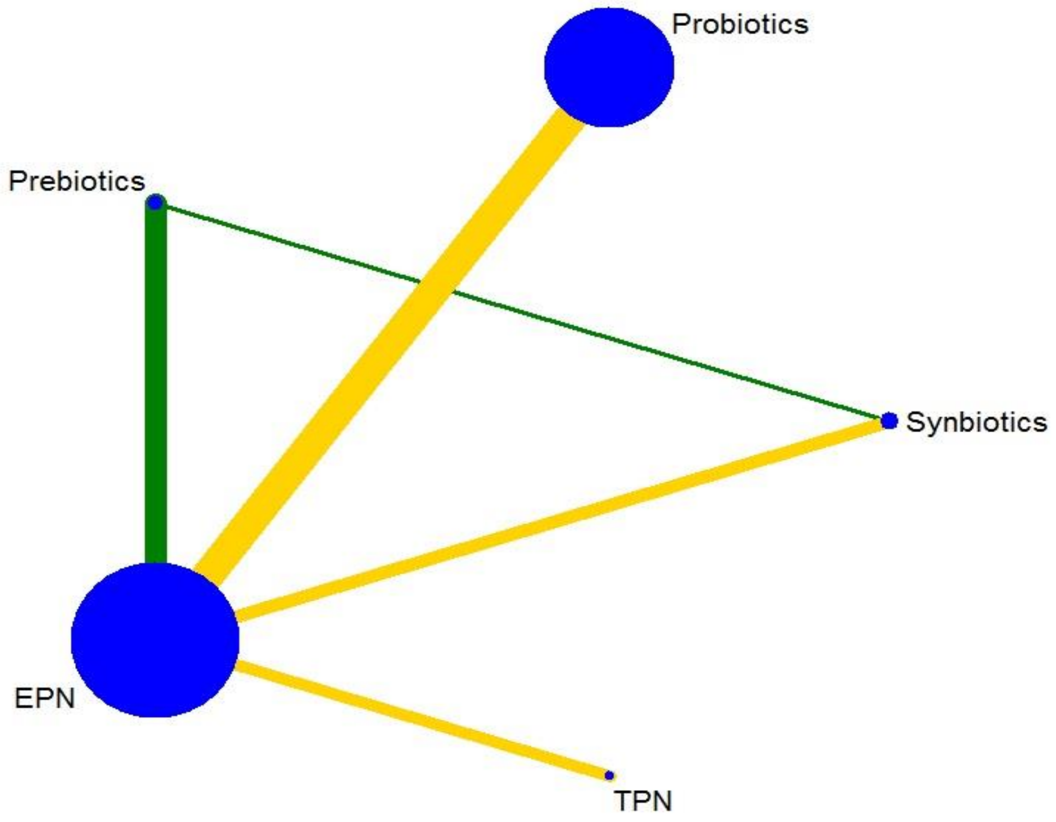

Supplement: Supplementary File 4 — Network plot of all intervention comparisons.pdf. [file Data_Sheet_4.PDF]
