# Supplementary material for: Probiotic Supplementation Prevents the Development of Ventilator-Associated Pneumonia for Mechanically Ventilated ICU Patients: A Systematic Review and Network Meta-analysis of Randomized Controlled Trials
Source: Front Nutr. 2022 Jul 8;9:919156. doi: 10.3389/fnut.2022.919156 (PMC9307490; doi:10.3389/fnut.2022.919156)
Supplement: Supplementary File 5 — Assessment of heterogeneity and inconsistency in networks.pdf. [file Data_Sheet_5.PDF]

Supplementary file 5
Assessment of heterogeneity in treatment for each outcome network

We assess heterogeneity between direct comparisons and global heterogeneity by looked at the I^2 weather it is high compared to the expected value (50%). The following graphs all show the results of heterogeneity between direct comparisons in each outcome network. The study number in the third column of each figure corresponds to the serial number of the references in Table 1. A:Synbiotics, B:Probiotics, C:Probiotics, D:Enteral nutrition and/or adjuvant peripheral parenteral nutrition, E: Total parenteral nutrition.

Figure S 5.1 Assesment of heterogeneity in networks

Outcome	Treatment	Study	Per-comparison I-squared(%)		Global I-squared(%)	
			Pair-wise	Network	Pair	Compairson
Ventilator-associated pneumonia	D vs A	7,8,23	62.7	62.7	73.33	73.43
	D vs B	6,10,12,14,16,18,19,24,25, 26,28,29,30	77.7	77.8		
	D vs C	1,20,21	84.4	75.0		
	E vs D	9,13,15,22,27	0	0		
Nosocomial infection	C vs A	4	--	0	63.37	58.27
	D vs A	2,4,23	0	0		
	D vs B	10,14,30	76.8	75.6		
	D vs C	1,4,20	75.8	75.7		
	E vs D	3,5,9,22	60.5	59.7		
Bloodstream infection	C vs A	4	--	0	27.25	15.27
	D vs A	4,7,23	31.1	32.3		
	D vs B	14,30,31	60.3	53.3		
	D vs C	1,4	0	0		
	E vs D	3,22	0	0		
Urinary tract infection	C vs A	4	--	0	49.01	45.02
	D vs A	2,4,7	0	0		
	D vs B	10,14,30,31	0	0		
	D vs C	1,4,20	76.1	77.9		
	E vs D	3,9,22	61.2	58.5		
Diarrhea	D vs A	2,8,23	36.3	36.3	84.67	84.83
	D vs B	10,12,16,18,24,26,30,31	88.8	88.9		
	D vs C	21	--	--		
	E vs D	9,13,22	66.2	68.3		
Hospital mortality	D vs A	2,7,8,23	0	0	13.11	13.51
	D vs B	11,12,18,19,25,30	40.7	40.9		
	D vs C	1,20,21	0	0		
	E vs D	3,5,13,22	0	0		
ICU mortality	C vs A	4	0	0	0	0
	D vs A	2,4,8	0	0		
	D vs B	10,19,24,25,26,28,30	0	0		
	D vs C	1,4	0	0		
	E vs D	9,13,22	37.6	37.8		
Hospital length of stay	D vs A	8	--	--	74.60	74.57
	D vs B	10,12,16,18,19,24,25,30	79.5	79.4		
	D vs C	1,21	86.8	86.8		
	E vs D	3,5,9,13,22,27	47.6	47.6		
ICU length of stay	C vs A	4	--	0	84.22	83.60
	D vs A	2,4,8,23	70.6	73.0		
	D vs B	1,10,11,12,16,17,18,19,24, 25,28,29,30	89.1	89.1		
	D vs C	4,20,21	39.2	36.6		
	E vs D	3,5,9,13,22,27	68.5	68.6		
Duration of mechanical ventilation	C vs A	4	--	0	91.04	90.55
	D vs A	2,4,8	89.3	89.0		
	D vs B	11,12,17,19,24,25,28,29,30	93.6	93.6		
	D vs C	1,4,20,21	83.2	85.1		
	E vs D	5,9,13	66.7	66.6		

Assessment of inconsistency in treatment for each outcome network

We use Node-splitting analysis to assess inconsistencies between direct and indirect comparisons, and‘design-by-treatment’ interaction model to assess global inconsistencies ($p<0.05$). A:Synbiotics, B:Probiotics, C:Probiotics, D:Enteral nutrition and/or adjuvant peripheral parenteral nutrition, E: Total parenteral nutrition.

Table S 5.2 Assessment of global heterogeneity in networks

Outcome	Node-splitting analysis of inconsistency		Global inconsistency	
	Comparison	P value	Chi-square	P value
Nosocomial infection	C vs A	0.9680	0.11	0.9449
Bloodstream infection	C vs A	0.1008	0.22	0.8972
Urinary tract infection	C vs A	0.7368	0.75	0.6861
ICU mortality	C vs A	0.9236	0.87	0.6463
ICU length of stay	C vs A	0.9764	1.30	0.5209
Duration of mechanical vetilation	C vs A	0.5445	2.59	0.2736
