# Supplementary material for: Probiotic Supplementation Prevents the Development of Ventilator-Associated Pneumonia for Mechanically Ventilated ICU Patients: A Systematic Review and Network Meta-analysis of Randomized Controlled Trials
Source: Front Nutr. 2022 Jul 8;9:919156. doi: 10.3389/fnut.2022.919156 (PMC9307490; doi:10.3389/fnut.2022.919156)
Supplement: Supplementary File 6 — Assessment of transitivity in networks for primary outcome.pdf. [file Data_Sheet_6.PDF]

Supplementary file 6

Assessment of transitivity

Figure S 6.1 Transitivity of mean age in primary outcome network

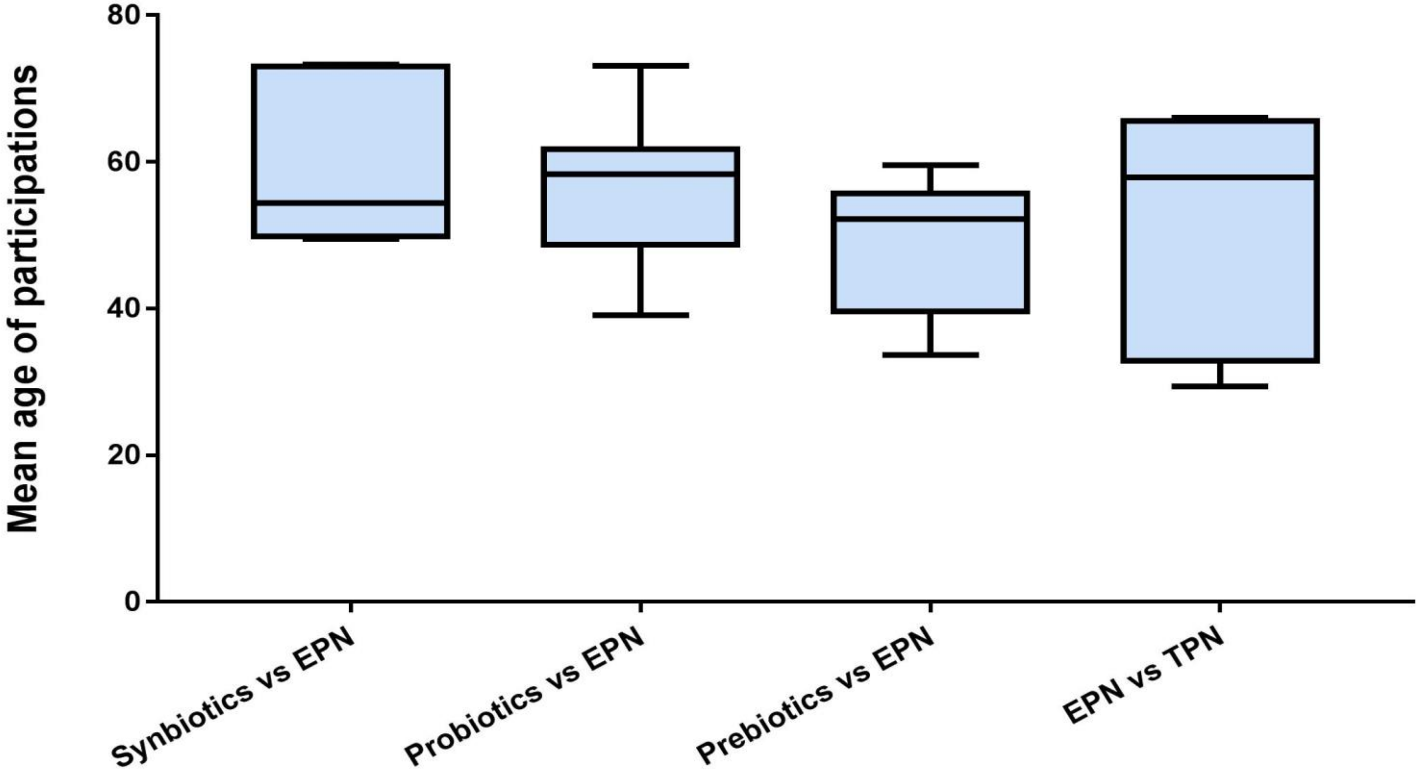

EPN: enteral nutrition and/or adjuvant peripheral parenteral nutrition. TPN: total parenteral nutrition.

Figure S 6.2 Transitivity of mean APACHE II score in primary outcome network

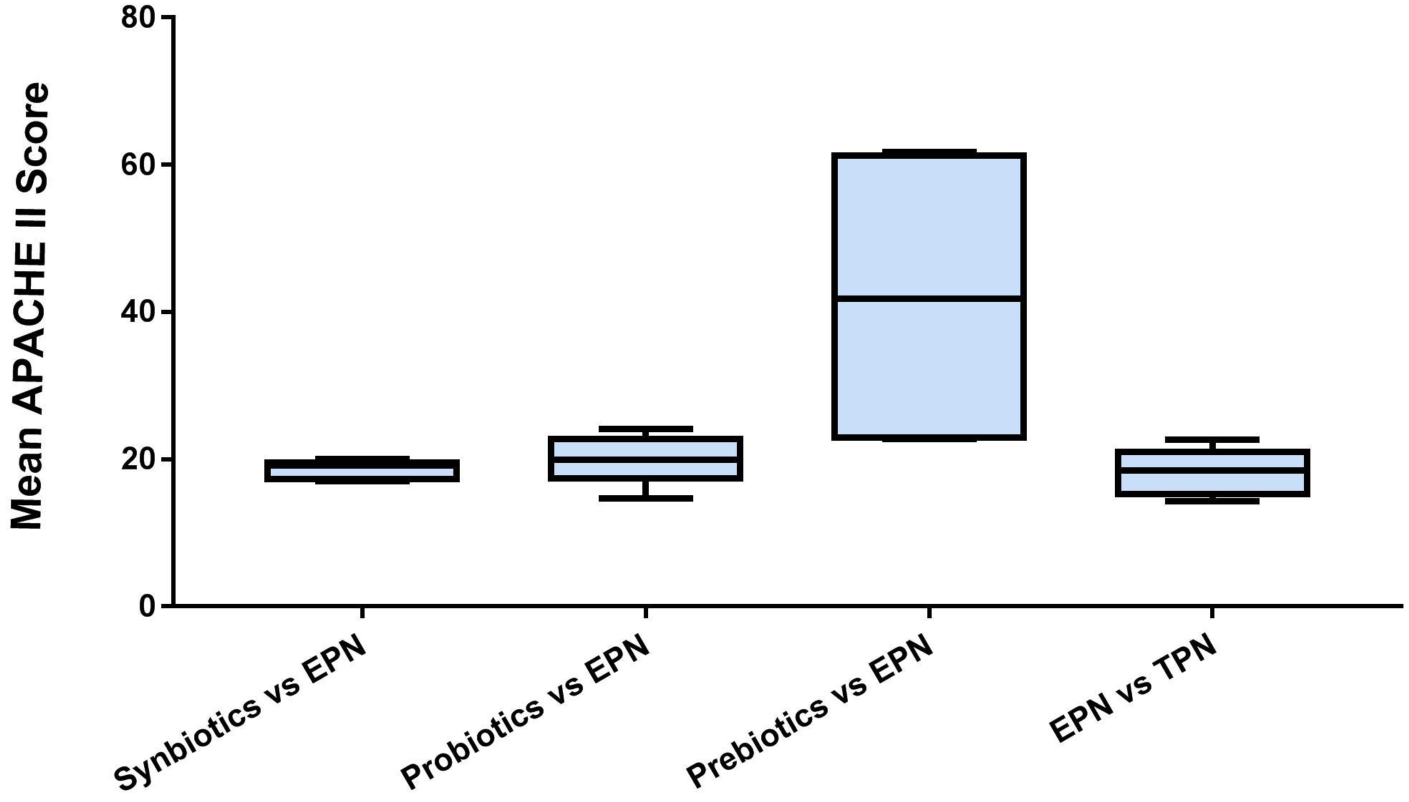

EPN: enteral nutrition and/or adjuvant peripheral parenteral nutrition. TPN: total parenteral nutrition.
