# Supplementary material for: Probiotic Supplementation Prevents the Development of Ventilator-Associated Pneumonia for Mechanically Ventilated ICU Patients: A Systematic Review and Network Meta-analysis of Randomized Controlled Trials
Source: Front Nutr. 2022 Jul 8;9:919156. doi: 10.3389/fnut.2022.919156 (PMC9307490; doi:10.3389/fnut.2022.919156)

Supplementary file 7
Comparison-adjusted funnel plot for each outcome form the network network meta-analysis

Figure S 7.1 Comparison-adjusted funnel plot for the network of ventilator-associated pneumonia in all comparisons

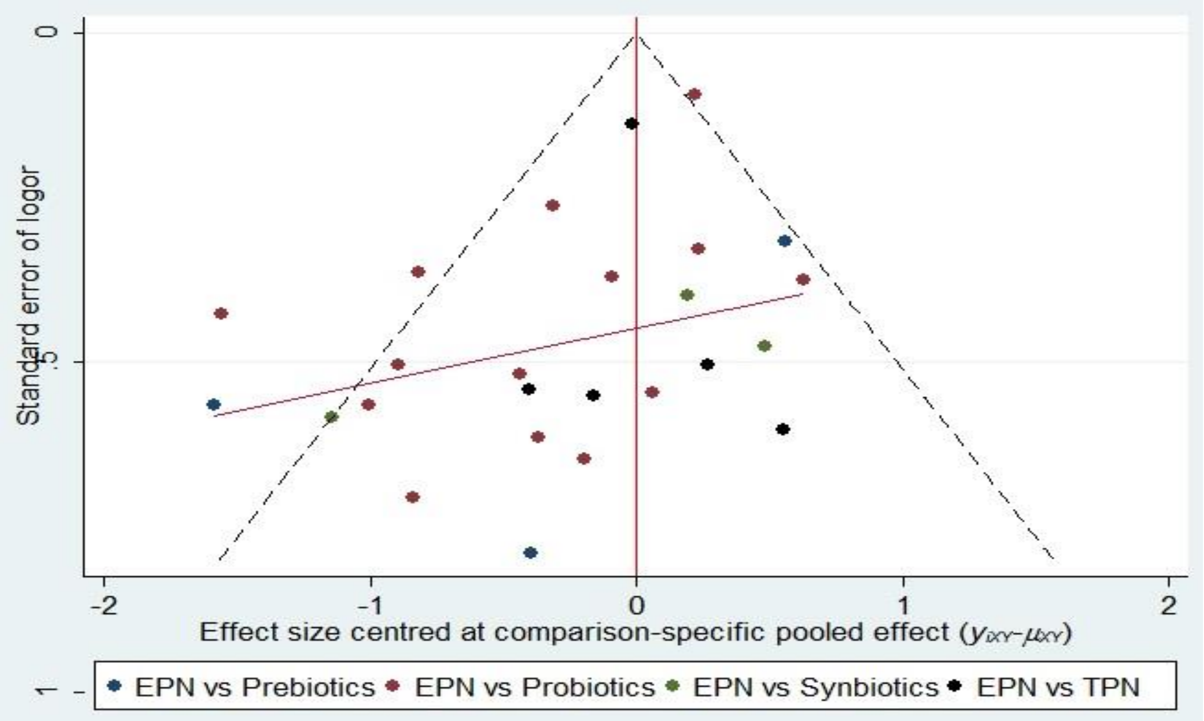

Figure S 7.2 Comparison-adjusted funnel plot for the network of nosocomial infection in all comparisons

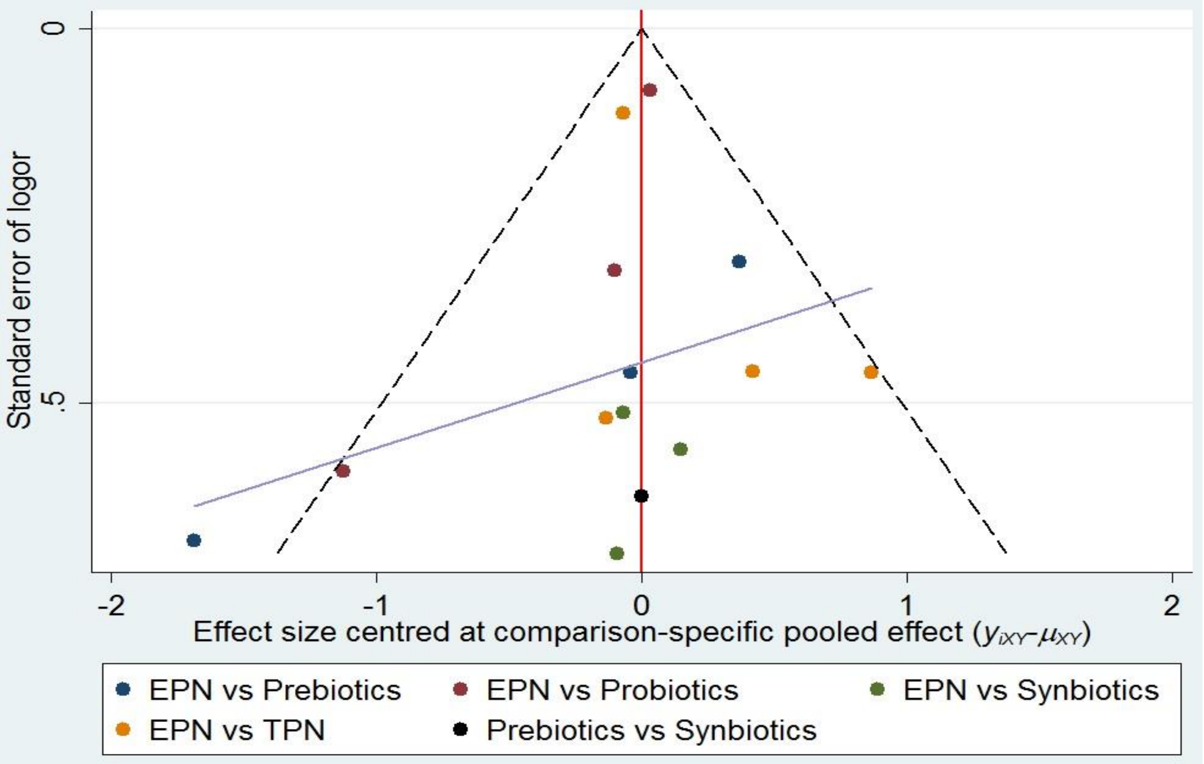

Figure S 7.3 Comparison-adjusted funnel plot for the network of bloodstream infection in all comparisons

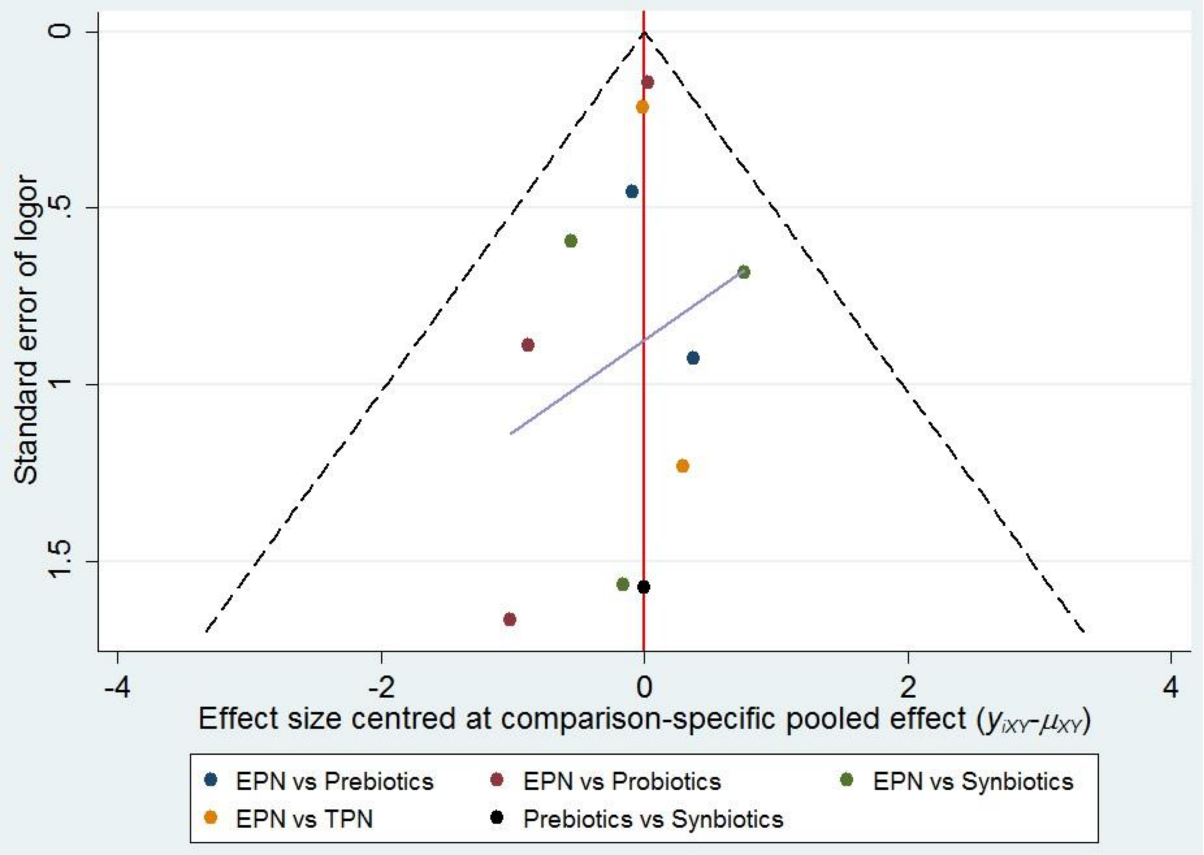

Figure S 7.4 Comparison-adjusted funnel plot for the network of urinary tract infection in all comparisons

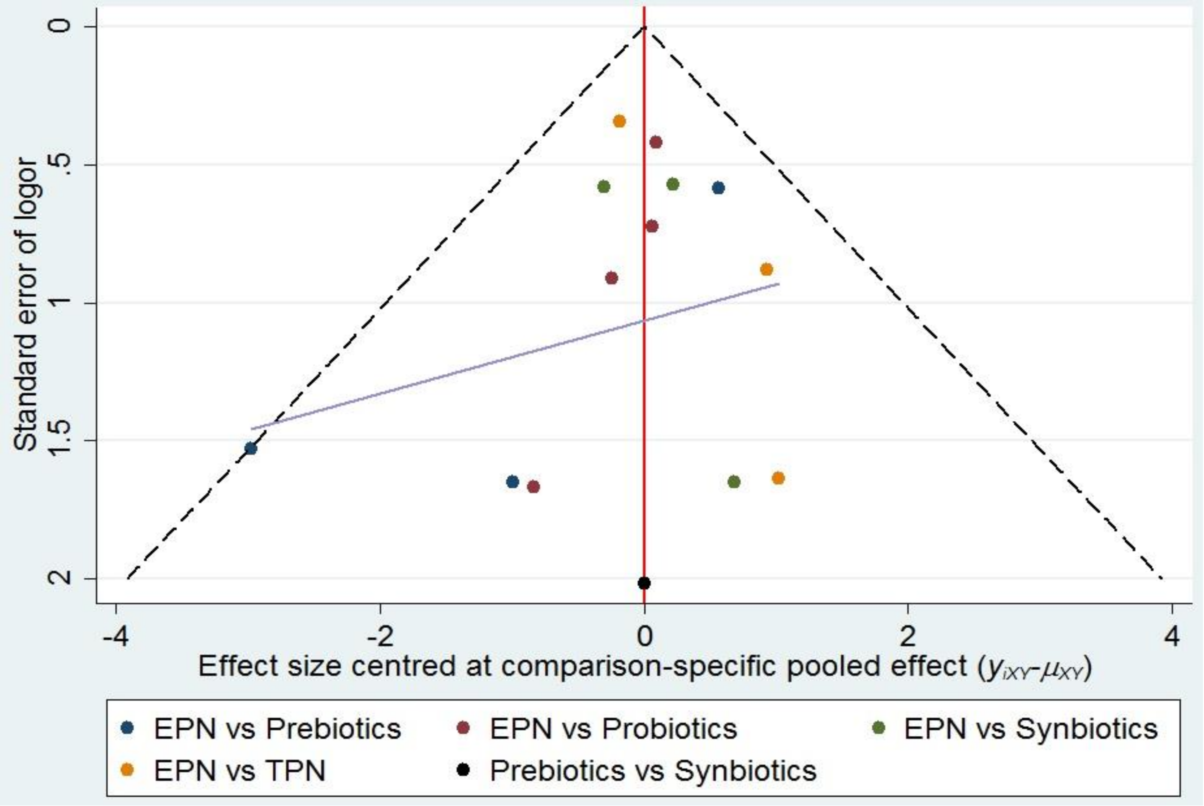

Figure S 7.5 Comparison-adjusted funnel plot for the network of diarrhea in all comparisons

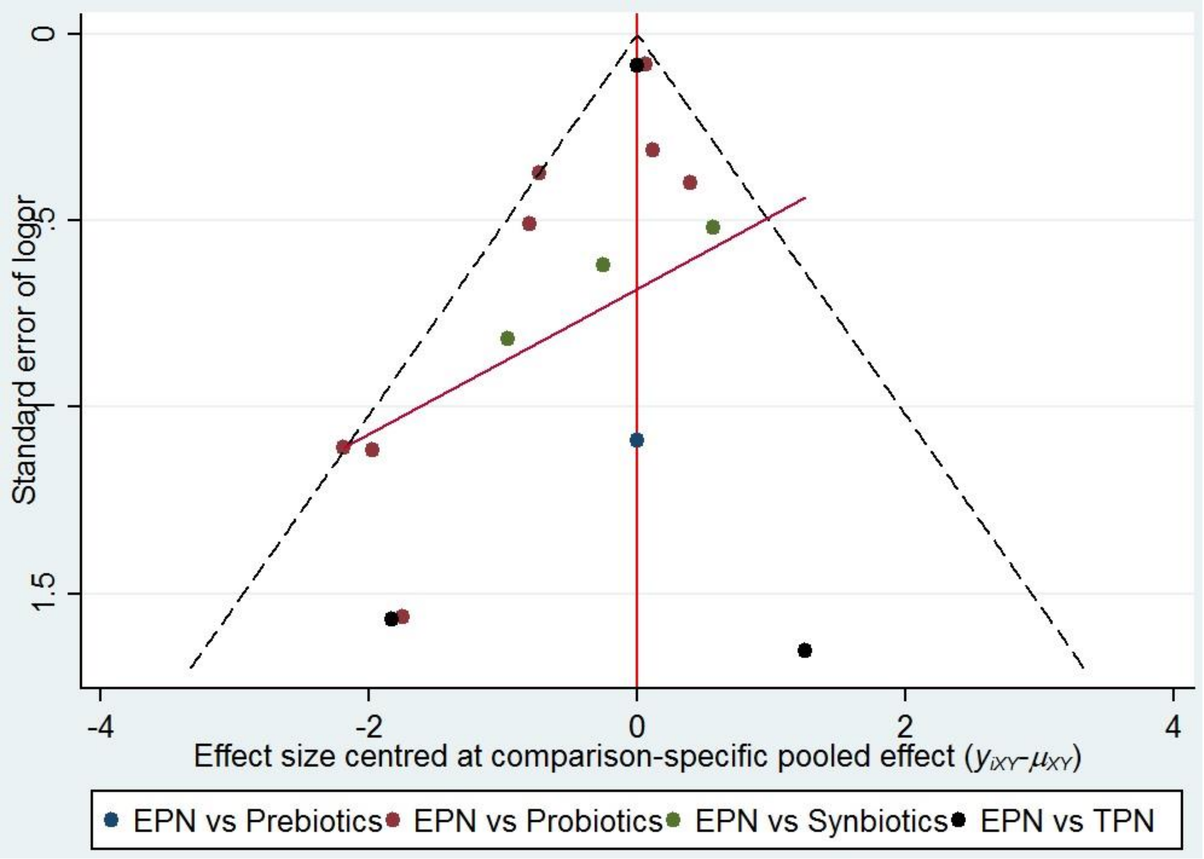

Figure S 7.6 Comparison-adjusted funnel plot for the network of hospital mortality in all comparisons

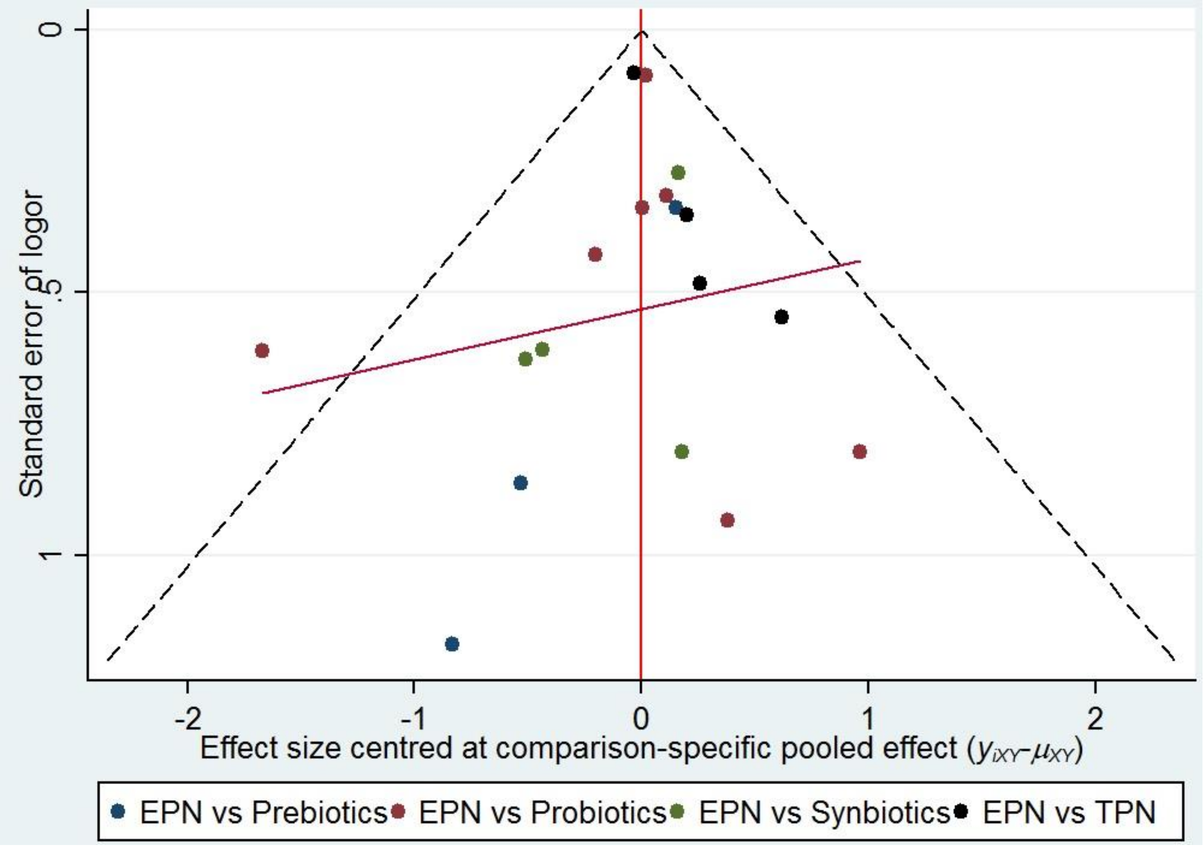

Figure S 7.7 Comparison-adjusted funnel plot for the network of ICU mortality comparisons

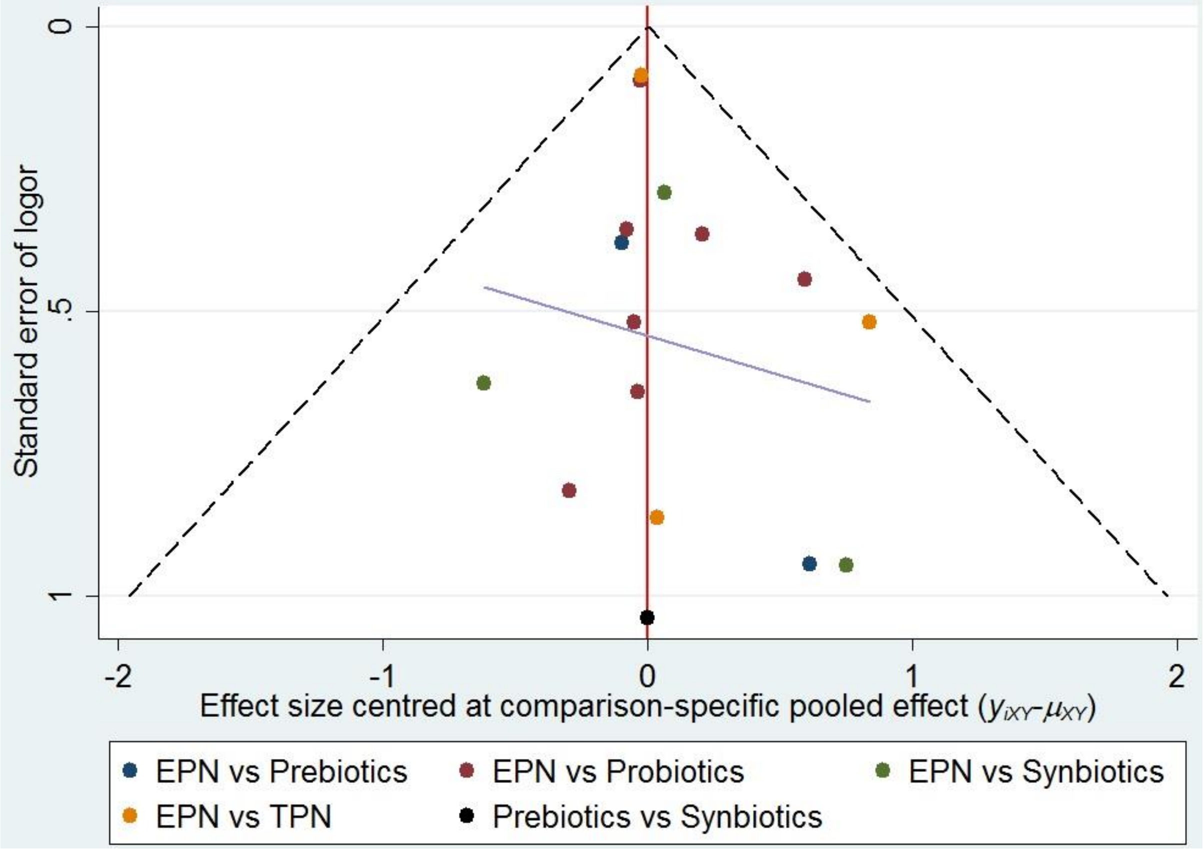

Figure S 7.8 Comparison-adjusted funnel plot for the network of hospital lengtn of stay comparisons

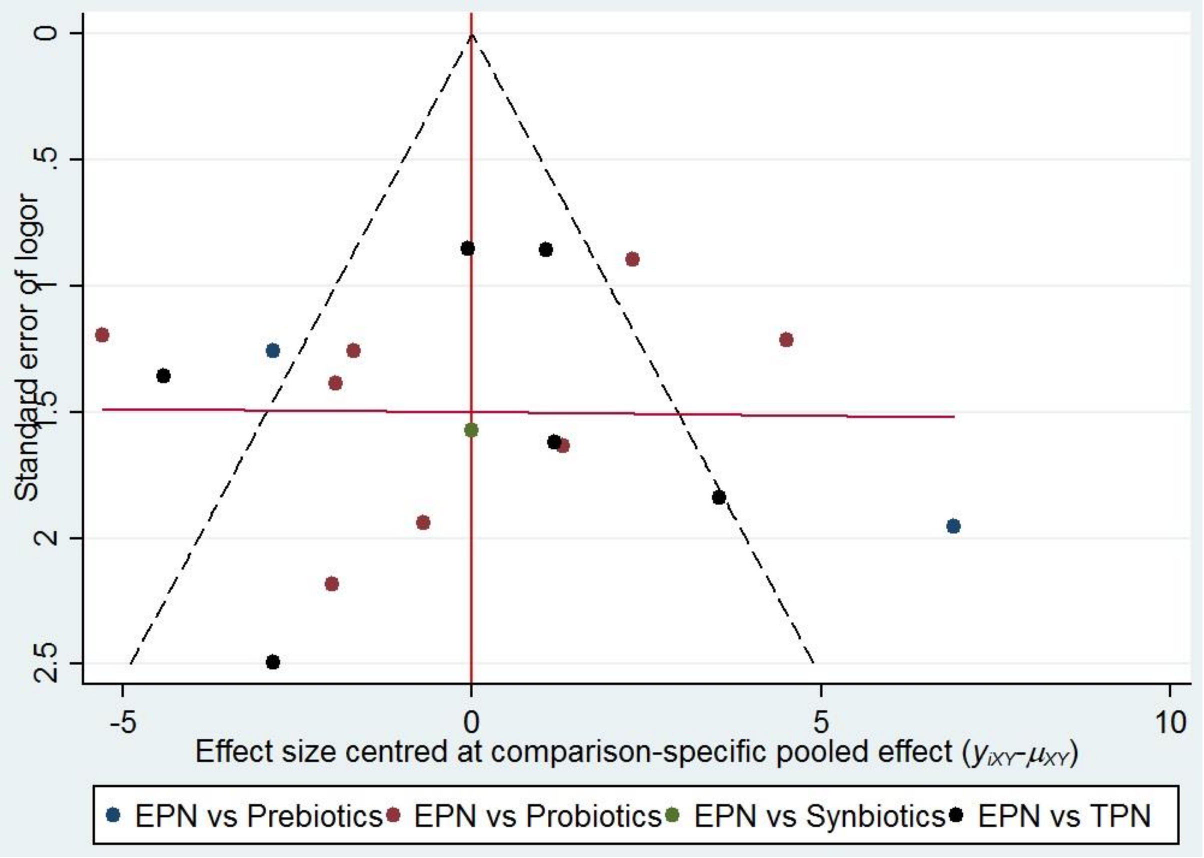

Figure S 7.9 Comparison-adjusted funnel plot for the network of ICU length of stay in all comparisons

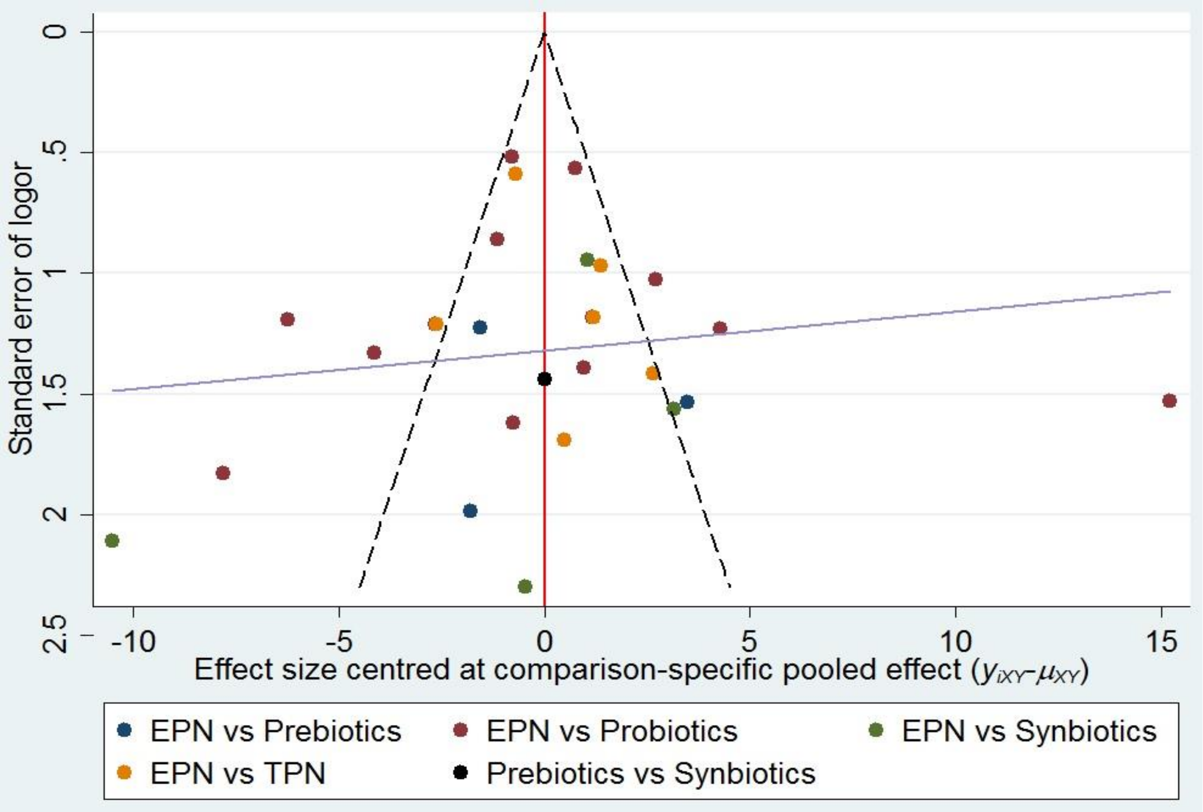

Figure S 7.10 Comparison-adjusted funnel plot for the network of the duration of mechanical ventilation in all comparisons

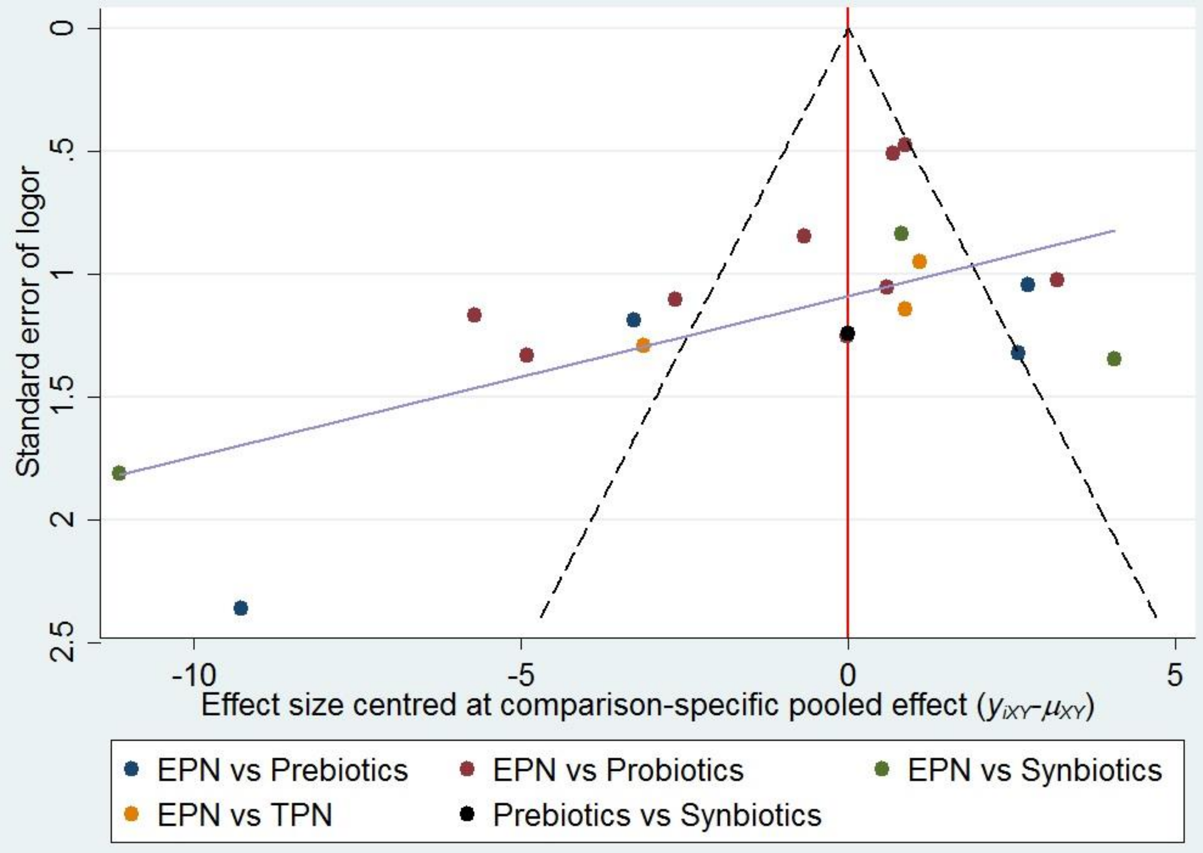

Supplement: Supplementary File 7 — Comparison-adjusted funnel plot for the network.pdf. [file Data_Sheet_7.PDF]
