# Supplementary material for: Probiotic Supplementation Prevents the Development of Ventilator-Associated Pneumonia for Mechanically Ventilated ICU Patients: A Systematic Review and Network Meta-analysis of Randomized Controlled Trials
Source: Front Nutr. 2022 Jul 8;9:919156. doi: 10.3389/fnut.2022.919156 (PMC9307490; doi:10.3389/fnut.2022.919156)
Supplement: Supplementary File 8 — GRADE for each outcome in networks.pdf. [file Data_Sheet_8.PDF]

Supplementary file 8
GRADE for the primary and second outcomes

Based on the above assessment of RoB for each comparison and the contribution matrix detailing contribution of each direct comparison to all network estimates, the following bar graphs show the percentage of low or moderate or high RoB contributions for each network estimate.

The judgements about study limitations in each direct comparison is shown at the beginning of the graph. Each bar corresponds to a NMA relative treatment effect and shows how much information comes from comparisons at low risk of bias [green] or moderate risk of bias [yellow].

Based on all the above information, we GRADEd each network estimate according to the following criteria.

(1)**Study limitations**: We downgraded by one level when the contributions from low RoB comparisons were less than 30% and contributions from moderate RoB comparisons were 70% or greater.

(2)**Imprecision**: For dichotomous variables, we considered a clinically meaningful threshold for OR to be 0.80 or 1.25 and downgraded the estimate if the OR point estimate is 1 or more and the lower limit of its confidence interval (CI) is below 0.80; or if the OR point estimate is less than 1 and the upper limit of its CI is above 1.25. For continuous variables, we downgrade when CI crosses null value or includes values favoring either treatment.

(3)**Inconsistency**: We rated two concepts, heterogeneity and incoherence (inconsistency), in this domain. For heterogeneity, we looked at the I^2 and found weather it is high compared to the expected value (50%). For inconsistency, we looked at the results of side splitting and 'design-by-treatment' interaction model. We did downgraded the comparisons with important inconsistency ($p<0.05$) (we could downgrade the same network estimate for both heterogeneity and inconsistency).

(4)**Indirectness**: We have assured transitivity in our network by limiting the included studies to critically ill adult patients. We assured that they did not violate transitivity of the network. Hence, we did not downgraded singly-connected nodes for indirectness because evaluation of transitivity for such nodes is clear.

(5)**Publication bias**: We managed to retrieve supplementary and unpublished information included in the available systematic reviews and network network meta-analysis, and we assessed publication bias using the comparison-adjusted funnel plots

Figure S 8.1 Contribution plot for ventilator-associated pneumonia and contribution of low or moderate RoB comparisons to each network estimate of ventilator-associated pneumonia

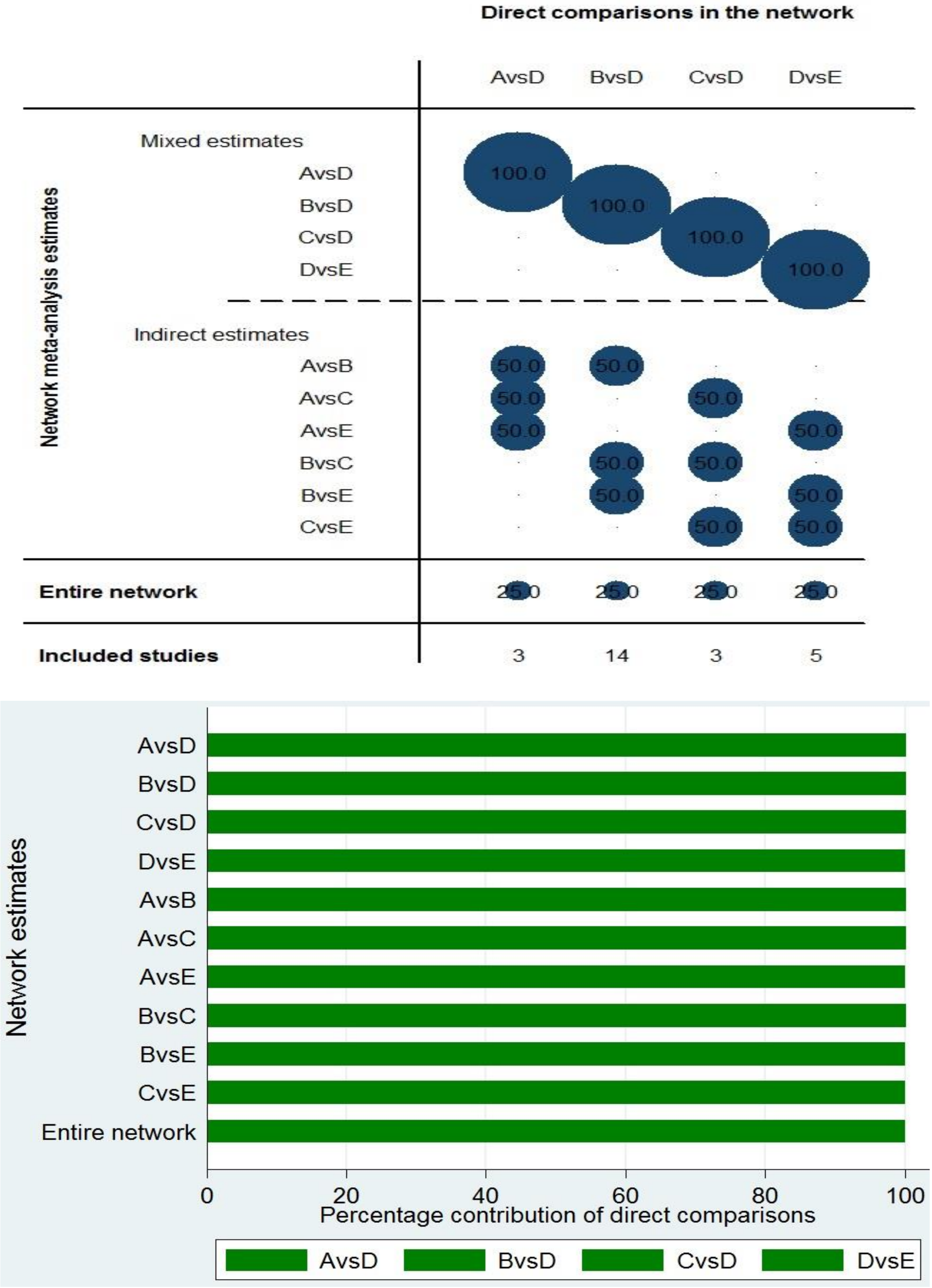

A: Synbiotics; B: Probiotics; C: Probiotics; D: EPN; E: TPN

Table S 8.1 Result of GRADE for ventilator-associated pneumonia

	Nature of the evidence	Study limitations	Imprecision	Inconsistency	Indirectness	Publication bias	Confidence	Downgrading due to
A vs B	Indirect estimated	No downgrade	No downgrade	No downgrade	No downgrade	No downgrade	HIGH	--
A vs C	Indirect estimated	No downgrade	No downgrade	No downgrade	No downgrade	No downgrade	HIGH	--
A vs D	Mixed estimated	No downgrade	Downgrade because point estimate >1.0 but lower limit<0.80	Downgrade because pair heterogeneity I^2=62.7%	No downgrade	No downgrade	LOW	Imprecision Inconsistency
A vs E	Indirect estimated	No downgrade	Downgrade because point estimate >1.0 but lower limit<0.80	No downgrade	No downgrade	No downgrade	MODERATE	Imprecision
B vs C	Indirect estimated	No downgrade	Downgrade because point estimate >1.0 but lower limit<0.80	No downgrade	No downgrade	No downgrade	MODERATE	Imprecision
B vs D	Mixed estimated	No downgrade	Downgrade because point estimate >1.0 but lower limit<0.80	No downgrade Downgrade because pair heterogeneity I^2=77.7%	No downgrade	Downgrade	VERY LOW	Imprecision Inconsistency Publication bias
B vs E	Indirect estimated	No downgrade	No downgrade	No downgrade	No downgrade	No downgrade	HIGH	--
C vs D	Mixed estimated	No downgrade	Downgrade because point estimate >1.0 but lower limit<0.80	Downgrade because pair heterogeneity I^2=84.4%	No downgrade	No downgrade	LOW	Imprecision Inconsistency
C vs E	Indirect estimated	No downgrade	No downgrade	No downgrade	No downgrade	Downgrade	MODERATE	Publication bias
D vs E	Mixed estimated	No downgrade	Downgrade because point estimate < 1.0 but upper limit >1.25	No downgrade	No downgrade	No downgrade	MODERATE	Imprecision
Ranking of treatments		No downgrade	No downgrade	Downgrade because global heterogeneity I^2=73.43%	No downgrade	Downgrade	LOW	Inconsistency Publication bias

A: Synbiotics; B: Probiotics; C: Probiotics; D: EPN; E: TPN

Figure S 8.2 Contribution plot for nosocomial infection and contribution of low or moderate RoB comparisons to each network estimate of nosocomial infection

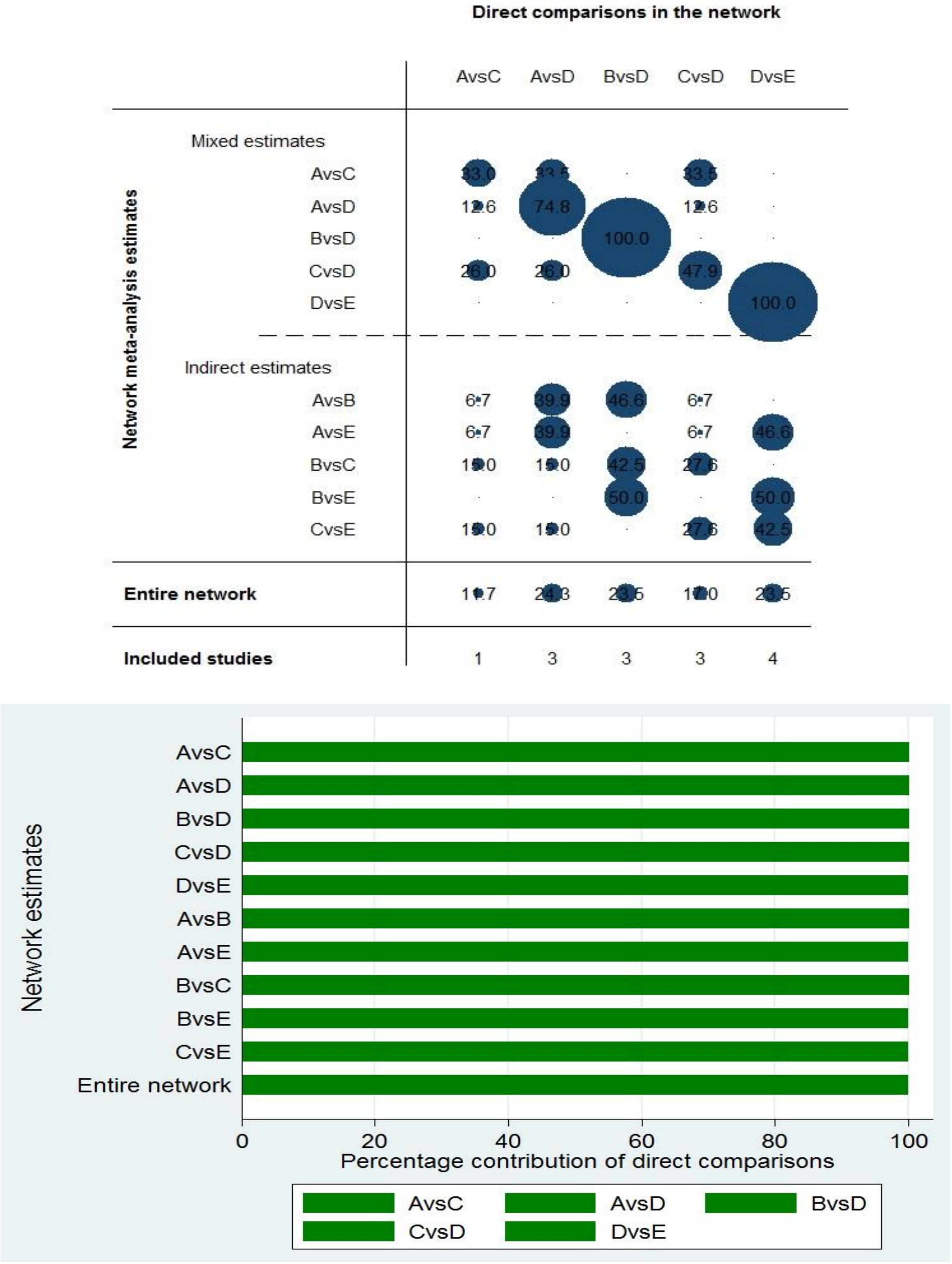

A: Synbiotics; B: Probiotics; C: Probiotics; D: EPN; E: TPN

Table S 8.2 Result of GRADE for nosocomial infection

	Nature of the evidence	Study limitations	Imprecision	Inconsistency	Indirectness	Publication bias	Confidence	Downgrading due to
A vs B	Indirect estimated	No downgrade	Downgrade because point estimate < 1.0 but upper limit >1.25	No downgrade	No downgrade	No downgrade	MODERATE	Imprecision
A vs C	Mixed estimated	No downgrade	Downgrade because point estimate >1.0 but lower limit<0.80	No downgrade	No downgrade	No downgrade	MODERATE	Imprecision
A vs D	Mixed estimated	No downgrade	Downgrade because point estimate < 1.0 but upper limit >1.25	No downgrade	No downgrade	No downgrade	MODERATE	Imprecision
A vs E	Indirect estimated	No downgrade	Downgrade because point estimate < 1.0 but upper limit >1.25	No downgrade	No downgrade	No downgrade	MODERATE	Imprecision
B vs C	Indirect estimated	No downgrade	Downgrade because point estimate >1.0 but lower limit<0.80	No downgrade	No downgrade	No downgrade	MODERATE	Imprecision -
B vs D	Mixed estimated	No downgrade	No downgrade	Downgrade because pair heterogeneity I^2=76.8%	No downgrade	No downgrade	MODERATE	Inconsistency
B vs E	Indirect estimated	No downgrade	No downgrade	No downgrade	No downgrade	No downgrade	HIGH	-
C vs D	Mixed estimated	No downgrade	Downgrade because point estimate >1.0 but lower limit<0.80	Downgrade because pair heterogeneity I^2=75.8%	No downgrade	No downgrade	LOW	Imprecision Inconsistency
C vs E	Indirect estimated	No downgrade	Downgrade because point estimate >1.0 but lower limit<0.80	No downgrade	No downgrade	Downgrade	LOW	Imprecision Publication bias
D vs E	Mixed estimated	No downgrade	Downgrade because point estimate >1.0 but lower limit<0.80	Downgrade because pair heterogeneity I^2=60.5%	No downgrade	No downgrade	LOW	Imprecision Inconsistency
Ranking of treatments		No downgrade	No downgrade	Downgrade because global heterogeneity I^2=58.27%	No downgrade	No downgrade	MODERATE	Inconsistency

A: Synbiotics; B: Probiotics; C: Probiotics; D: EPN; E: TPN

Figure S 8.3 Contribution plot for bloodstream infection and contribution of low or moderate RoB comparisons to each network estimate of bloodstream infection

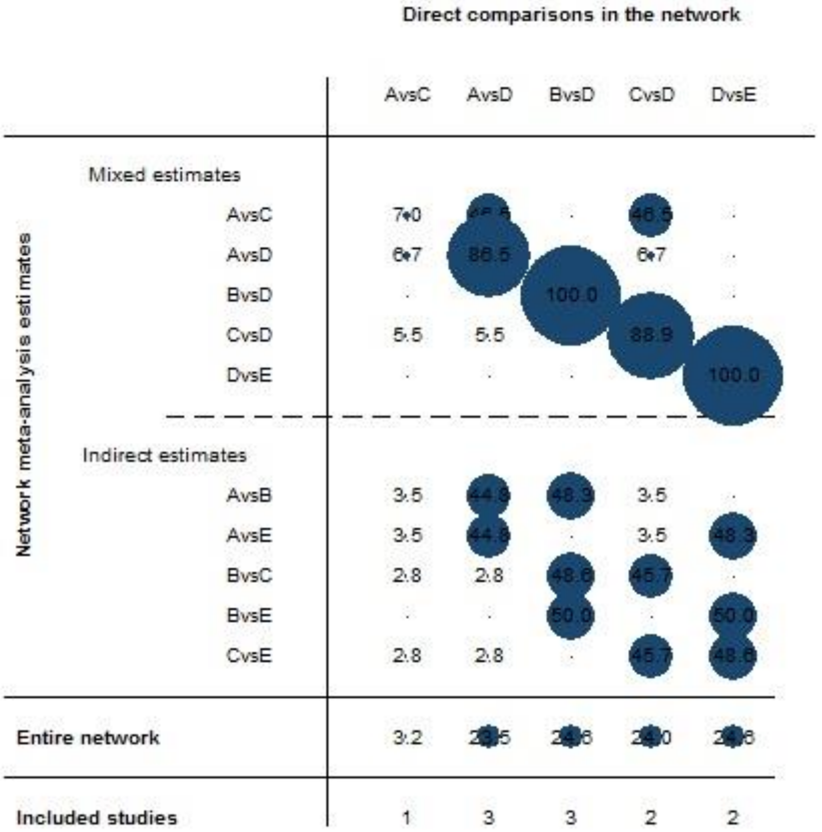

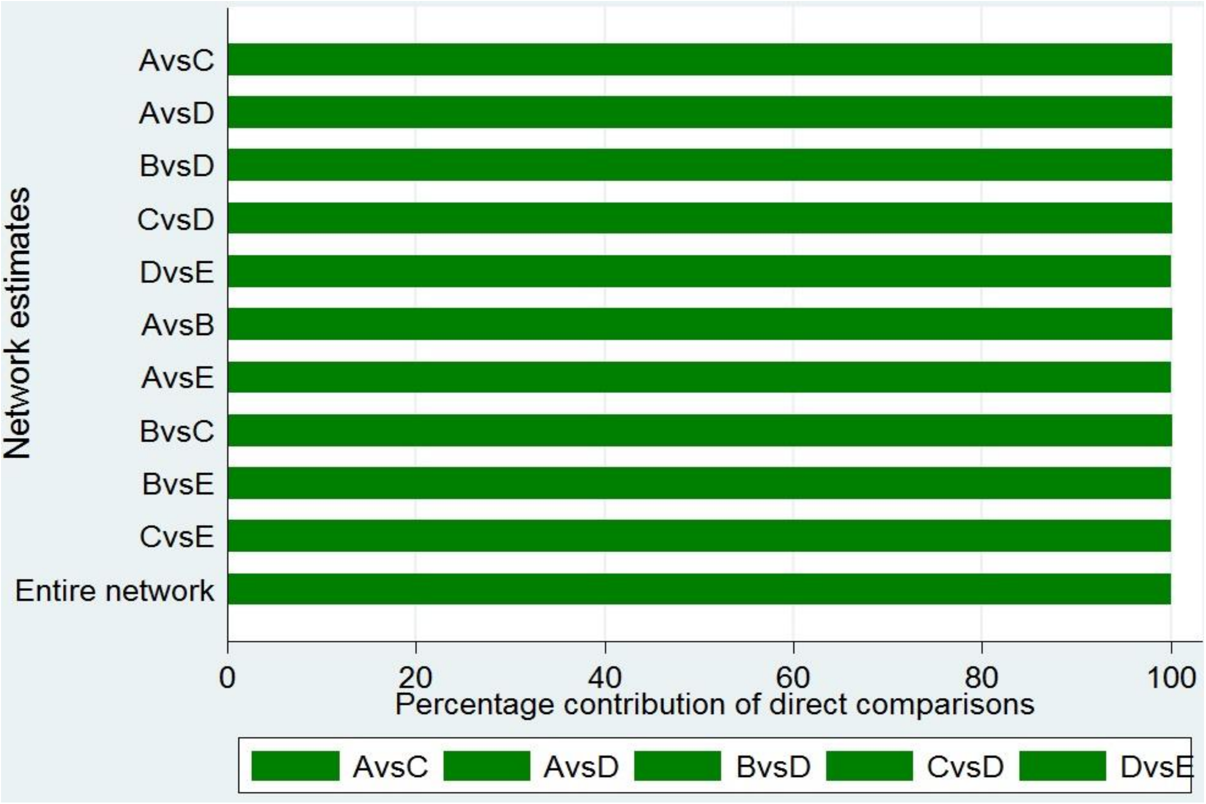

A: Synbiotics; B: Probiotics; C: Probiotics; D: EPN; E: TPN

Table S 8.3 Result of GRADE for bloodstream infection

	Nature of the evidence	Study limitations	Imprecision	Inconsistency	Indirectness	Publication bias	Confidence	Downgrading due to
A vs B	Indirect estimated	No downgrade	Downgrade because point estimate >1.0 but lower limit<0.80	No downgrade	No downgrade	No downgrade	MODERATE	Imprecision
A vs C	Mixed estimated	No downgrade	Downgrade because point estimate < 1.0 but upper limit >1.25	No downgrade	No downgrade	No downgrade	MODERATE	Imprecision
A vs D	Mixed estimated	No downgrade	Downgrade because point estimate < 1.0 but upper limit >1.25	No downgrade	No downgrade	No downgrade	MODERATE	Imprecision
A vs E	Indirect estimated	No downgrade	Downgrade because point estimate < 1.0 but upper limit >1.25	No downgrade	No downgrade	No downgrade	MODERATE	Imprecision
B vs C	Indirect estimated	No downgrade	Downgrade because point estimate >1.0 but lower limit<0.80	No downgrade	No downgrade	No downgrade	MODERATE	Imprecision
B vs D	Mixed estimated	No downgrade	Downgrade because point estimate >1.0 but lower limit<0.80	Downgrade because pair heterogeneity I^2=60.3%	No downgrade	No downgrade	LOW	Imprecision Inconsistency
B vs E	Indirect estimated	No downgrade	Downgrade because point estimate >1.0 but lower limit<0.80	No downgrade	No downgrade	No downgrade	MODERATE	Imprecision -
C vs D	Mixed estimated	No downgrade	Downgrade because point estimate >1.0 but lower limit<0.80	No downgrade	No downgrade	No downgrade	MODERATE	Imprecision -
C vs E	Indirect estimated	No downgrade	Downgrade because point estimate < 1.0 but upper limit >1.25	No downgrade	No downgrade	No downgrade	MODERATE	Imprecision -
D vs E	Mixed estimated	No downgrade	Downgrade because point estimate >1.0 but lower limit<0.80	No downgrade	No downgrade	No downgrade	MODERATE	Imprecision -
Ranking of treatments		No downgrade	No downgrade	No downgrade	No downgrade	No downgrade	HIGH	--

A: Synbiotics; B: Probiotics; C: Probiotics; D: EPN; E: TPN

Figure S 8.4 Contribution plot for urinary tract infection and contribution of low or moderate RoB comparisons to each network estimate of urinary tract infection

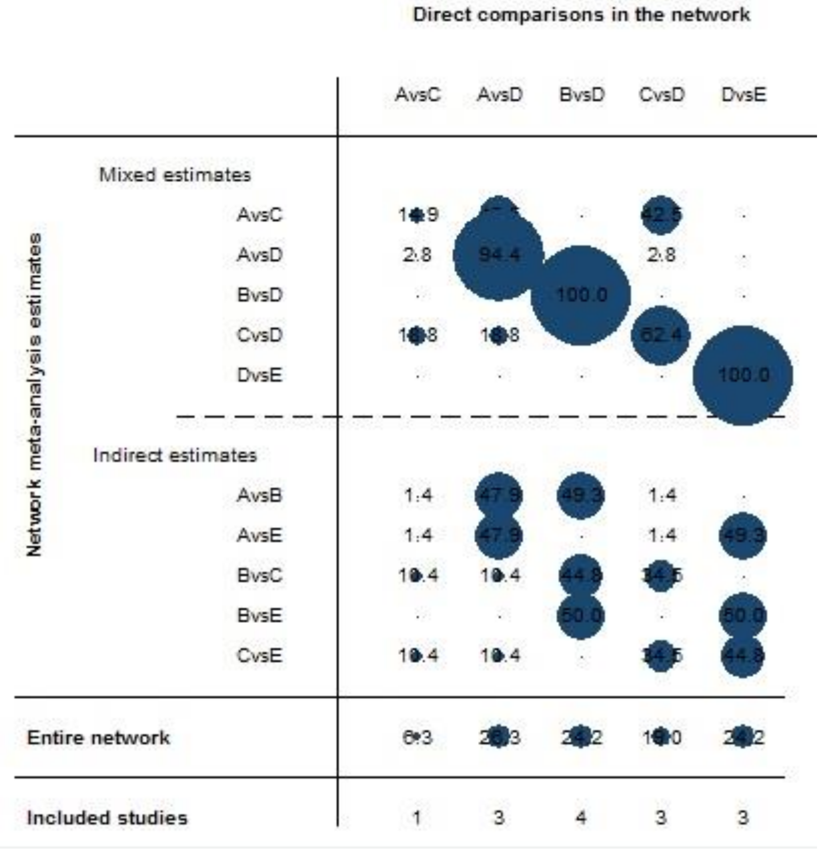

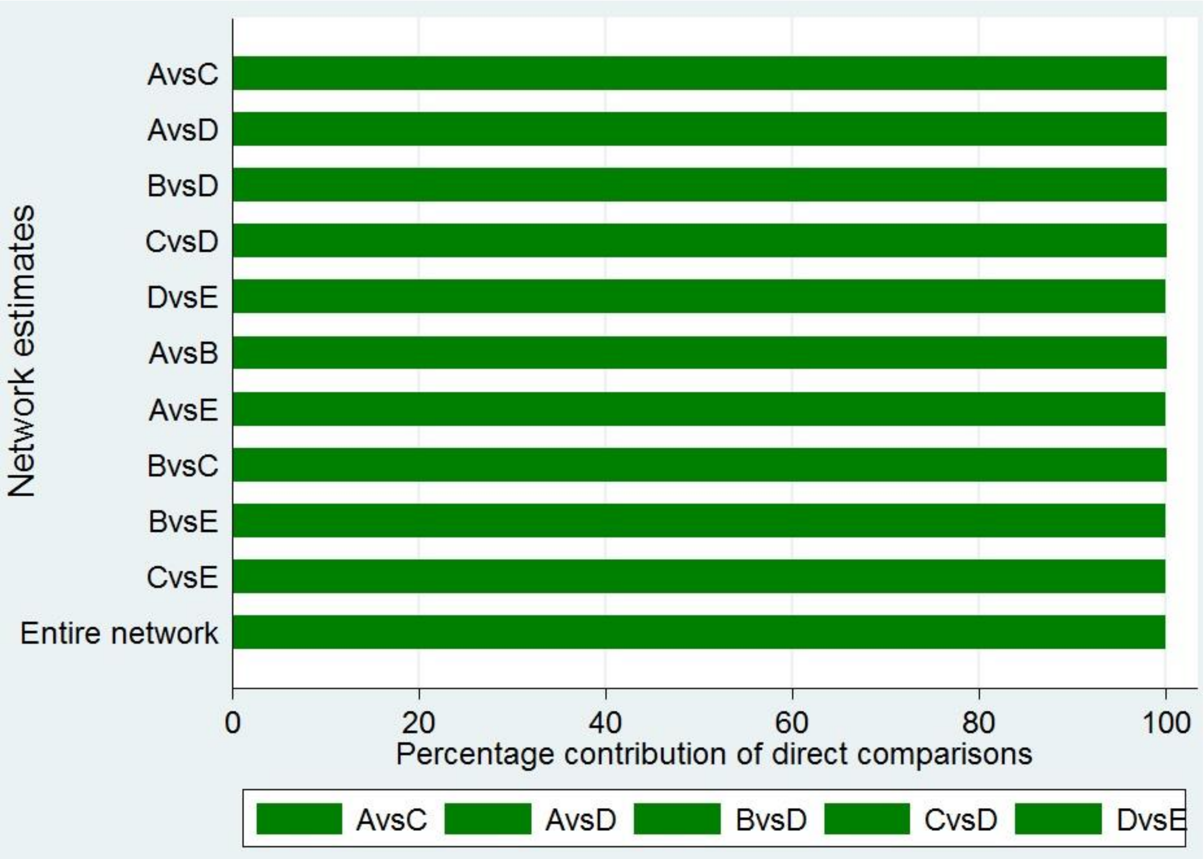

A: Synbiotics; B: Probiotics; C: Probiotics; D: EPN; E: TPN

Table S 8.4 Result of GRADE for urinary tract infection

	Nature of the evidence	Study limitations	Imprecision	Inconsistency	Indirectness	Publication bias	Confidence	Downgrading due to
A vs B	Indirect estimated	No downgrade	Downgrade because point estimate < 1.0 but upper limit >1.25	No downgrade	No downgrade	No downgrade	MODERATE	Imprecision
A vs C	Mixed estimated	No downgrade	Downgrade because point estimate < 1.0 but upper limit >1.25	No downgrade	No downgrade	No downgrade	MODERATE	Imprecision
A vs D	Mixed estimated	No downgrade	Downgrade because point estimate < 1.0 but upper limit >1.25	No downgrade	No downgrade	No downgrade	MODERATE	Imprecision
A vs E	Indirect estimated	No downgrade	Downgrade because point estimate >1.0 but lower limit<0.80	No downgrade	No downgrade	No downgrade	MODERATE	Imprecision
B vs C	Indirect estimated	No downgrade	Downgrade because point estimate >1.0 but lower limit<0.80	No downgrade	No downgrade	No downgrade	MODERATE	Imprecision
B vs D	Mixed estimated	No downgrade	Downgrade because point estimate < 1.0 but upper limit >1.25	No downgrade	No downgrade	No downgrade	MODERATE	Imprecision
B vs E	Indirect estimated	No downgrade	Downgrade because point estimate >1.0 but lower limit<0.80	No downgrade	No downgrade	No downgrade	MODERATE	Imprecision
C vs D	Mixed estimated	No downgrade	Downgrade because point estimate < 1.0 but upper limit >1.25	Downgrade because pair heterogeneity I^2=76.1%	No downgrade	No downgrade	LOW	Imprecision Inconsistency
C vs E	Indirect estimated	No downgrade	Downgrade because point estimate >1.0 but lower limit<0.80	No downgrade	No downgrade	No downgrade	MODERATE	Imprecision
D vs E	Mixed estimated	No downgrade	Downgrade because point estimate >1.0 but lower limit<0.80	Downgrade because pair heterogeneity I^2=61.2%	No downgrade	No downgrade	LOW	Imprecision Inconsistency
Ranking of treatments		No downgrade	No downgrade	No downgrade	No downgrade	No downgrade	HIGH	-

A: Synbiotics; B: Probiotics; C: Probiotics; D: EPN; E: TPN

Figure S 8.5 Contribution plot for diarrhea and contribution of low or moderate RoB comparisons to each network estimate of diarrhea

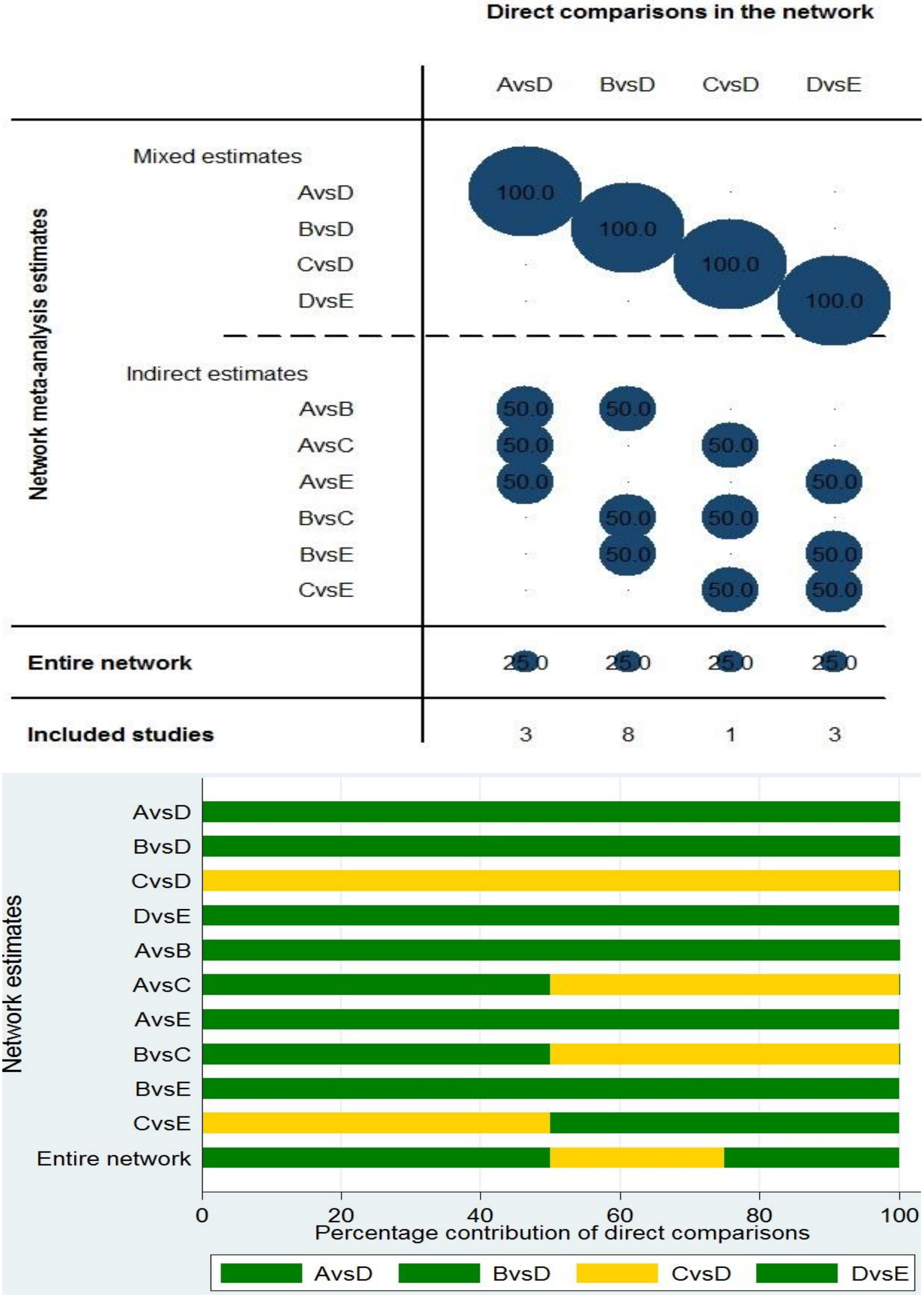

A: Synbiotics; B: Probiotics; C: Probiotics; D: EPN; E: TPN

Table S 8.5 Result of GRADE for diarrhea

	Nature of the evidence	Study limitations	Imprecision	Inconsistency	Indirectness	Publication bias	Confidence	Downgrading due to
A vs B	Indirect estimated	No downgrade	No downgrade	No downgrade	No downgrade	No downgrade	HIGH	--
A vs C	Indirect estimated	No downgrade	Downgrade because point estimate < 1.0 but upper limit >1.25	No downgrade	No downgrade	No downgrade	MODERATE	Imprecision
A vs D	Mixed estimated	No downgrade	No downgrade	No downgrade	No downgrade	No downgrade	HIGH	-
A vs E	Indirect estimated	No downgrade	Downgrade because point estimate < 1.0 but upper limit >1.25	No downgrade	No downgrade	No downgrade	MODERATE	Imprecision
B vs C	Indirect estimated	No downgrade	Downgrade because point estimate >1.0 but lower limit<0.80	No downgrade	No downgrade	No downgrade	MODERATE	Imprecision
B vs D	Mixed estimated	No downgrade	Downgrade because point estimate < 1.0 but upper limit >1.25	Downgrade because pair heterogeneity I^2=88.8%	No downgrade	No downgrade	LOW	Imprecision Inconsistency
B vs E	Indirect estimated	No downgrade	Downgrade because point estimate >1.0 but lower limit<0.80	No downgrade	No downgrade	No downgrade	MODERATE	Imprecision
C vs D	Mixed estimated	Downgrade because ＞70% contribution from moderate Rob comparisons	No downgrade	No downgrade	No downgrade	No downgrade	MODERATE	Study limitation
C vs E	Indirect estimated	No downgrade	Downgrade because point estimate >1.0 but lower limit<0.80	No downgrade	No downgrade	No downgrade	MODERATE	Imprecision
D vs E	Mixed estimated	No downgrade	No downgrade	Downgrade because pair heterogeneity I^2=66.2%	No downgrade	No downgrade	MODERATE	Inconsistency
Ranking of treatments		No downgrade	No downgrade	Downgrade because global heterogeneity I^2=84.83%	No downgrade	No downgrade	MODERATE	Inconsistency

A: Synbiotics; B: Probiotics; C: Probiotics; D: EPN; E: TPN

Figure S 8.6 Contribution plot for hospital mortality and contribution of low or moderate RoB comparisons to each network estimate of hospital mortality

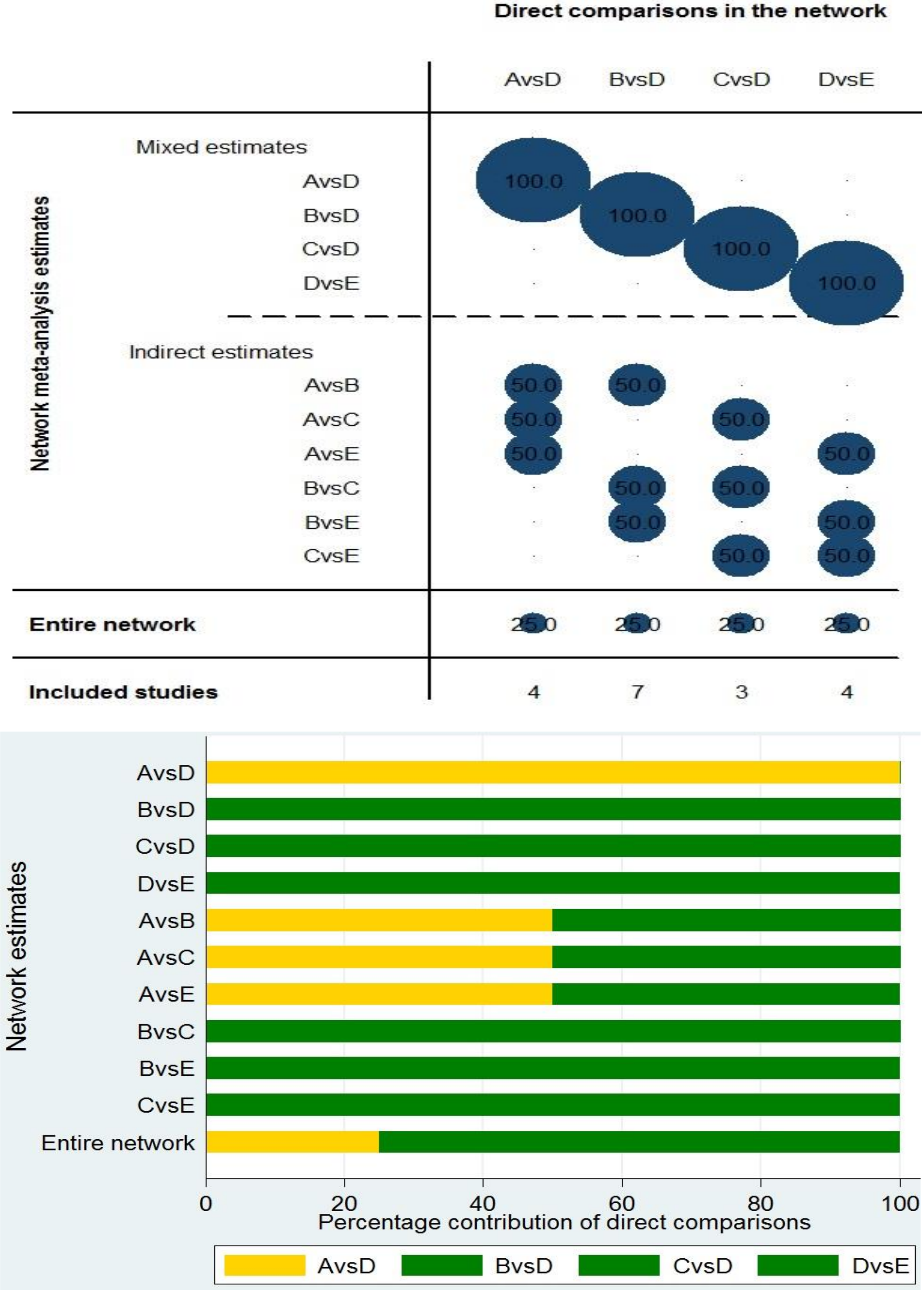

A: Synbiotics; B: Probiotics; C: Probiotics; D: EPN; E: TPN

Table S 8.6 Result of GRADE for hospital mortality

	Nature of the evidence	Study limitations	Imprecision	Inconsistency	Indirectness	Publication bias	Confidence	Downgrading due to
A vs B	Indirect estimated	No downgrade	No downgrade	No downgrade	No downgrade	No downgrade	HIGH	-
A vs C	Indirect estimated	No downgrade	No downgrade	No downgrade	No downgrade	No downgrade	HIGH	-
A vs D	Mixed estimated	Downgrade because ＞70% contribution from moderate Rob comparisons	No downgrade	No downgrade	No downgrade	No downgrade	MODERATE	Study limitations
A vs E	Indirect estimated	No downgrade	No downgrade	No downgrade	No downgrade	No downgrade	HIGH	-
B vs C	Indirect estimated	No downgrade	No downgrade	No downgrade	No downgrade	No downgrade	HIGH	--
B vs D	Mixed estimated	No downgrade	No downgrade	Downgrade because pair heterogeneity I^2=58.6%	No downgrade	Downgrade	LOW	Inconsistency Publication bias
B vs E	Indirect estimated	No downgrade	No downgrade	No downgrade	No downgrade	No downgrade	HIGH	--
C vs D	Mixed estimated	No downgrade	No downgrade	No downgrade	No downgrade	No downgrade	HIGH	--
C vs E	Indirect estimated	No downgrade	No downgrade	No downgrade	No downgrade	No downgrade	HIGH	--
D vs E	Mixed estimated	No downgrade	No downgrade	No downgrade	No downgrade	No downgrade	HIGH	--
Ranking of treatments		No downgrade	No downgrade	No downgrade	No downgrade	Downgrade	MODERATE	Publication bias

A: Synbiotics; B: Probiotics; C: Probiotics; D: EPN; E: TPN

Figure S 8.7 Contribution plot for ICU mortality and contribution of low or moderate RoB comparisons to each network estimate of ICU mortality

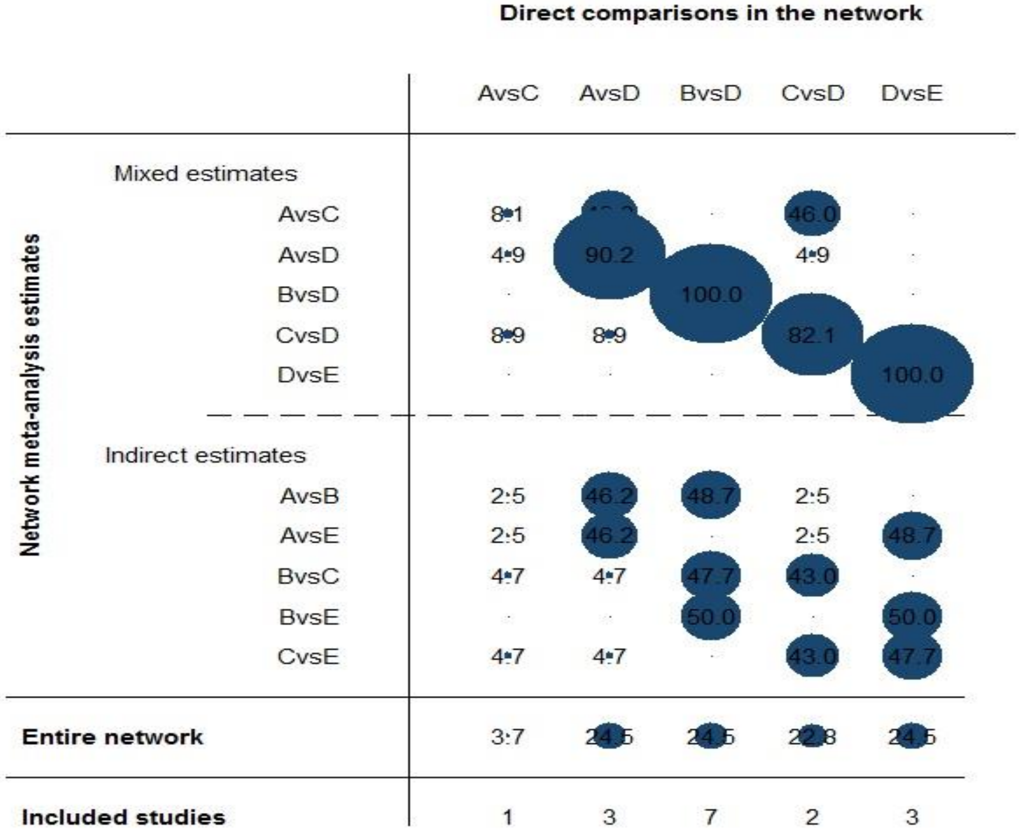

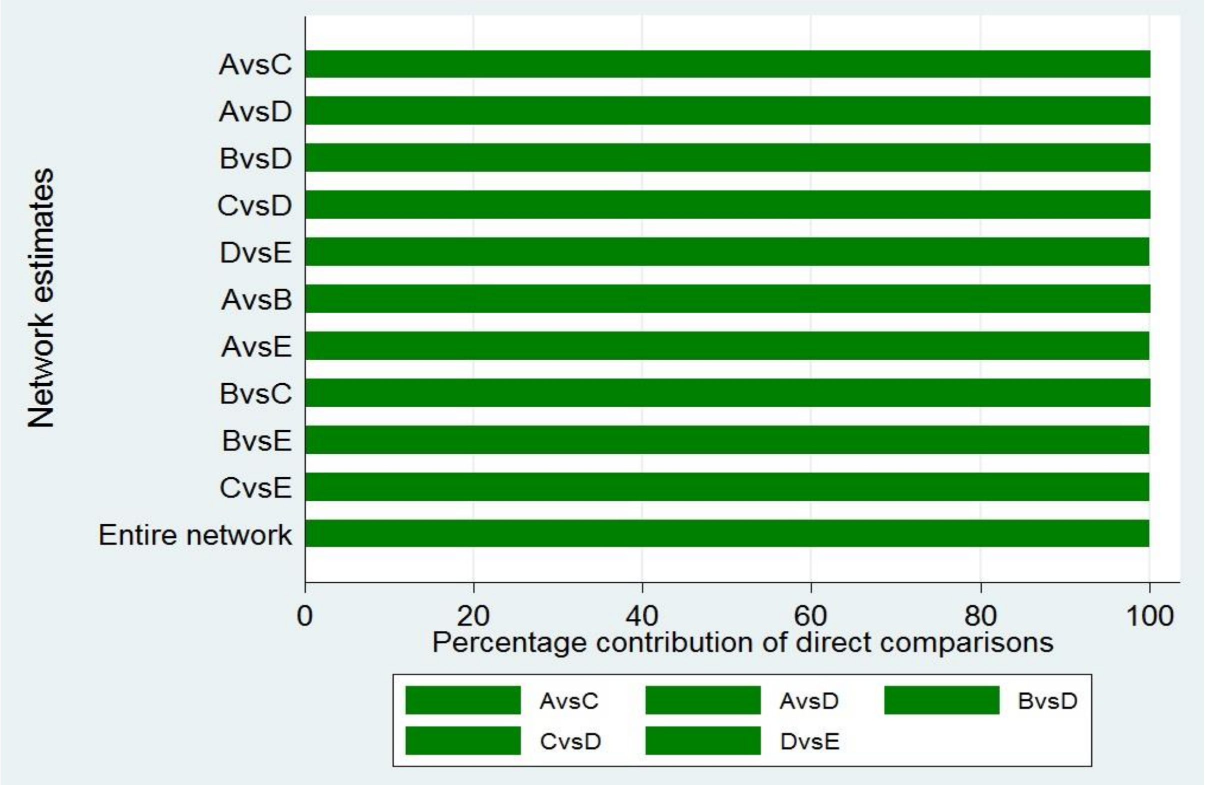

A: Synbiotics; B: Probiotics; C: Probiotics; D: EPN; E: TPN

Table S 8.7 Result of GRADE for ICU mortality

	Nature of the evidence	Study limitations	Imprecision	Inconsistency	Indirectness	Publication bias	Confidence	Downgrading due to
A vs B	Indirect estimated	No downgrade	Downgrade because point estimate < 1.0 but upper limit >1.25	No downgrade	No downgrade	No downgrade	MODERATE	Imprecision
A vs C	Mixed estimated	No downgrade	Downgrade because point estimate >1.0 but lower limit<0.80	No downgrade	No downgrade	No downgrade	MODERATE	Imprecision
A vs D	Mixed estimated	No downgrade	No downgrade	No downgrade	No downgrade	No downgrade	MODERATE	Imprecision
A vs E	Indirect estimated	No downgrade	Downgrade because point estimate < 1.0 but upper limit >1.25	No downgrade	No downgrade	No downgrade	MODERATE	Imprecision
B vs C	Indirect estimated	No downgrade	Downgrade because point estimate < 1.0 but upper limit >1.25	No downgrade	No downgrade	No downgrade	MODERATE	Imprecision
B vs D	Mixed estimated	No downgrade	Downgrade because point estimate < 1.0 but upper limit >1.25	No downgrade	No downgrade	No downgrade	MODERATE	Imprecision
B vs E	Indirect estimated	No downgrade	Downgrade because point estimate < 1.0 but upper limit >1.25	No downgrade	No downgrade	No downgrade	MODERATE	Imprecision
C vs D	Mixed estimated	No downgrade	Downgrade because point estimate >1.0 but lower limit<0.80	No downgrade	No downgrade	No downgrade	MODERATE	Imprecision
C vs E	Indirect estimated	No downgrade	No downgrade	No downgrade	No downgrade	No downgrade	HIGH	--
D vs E	Mixed estimated	No downgrade	Downgrade because point estimate < 1.0 but upper limit >1.25	No downgrade	No downgrade	No downgrade	MODERATE	Imprecision
Ranking of treatments		No downgrade	No downgrade	No downgrade	No downgrade	No downgrade	HIGH	-

A: Synbiotics; B: Probiotics; C: Probiotics; D: EPN; E: TPN

Figure S 8.8 Contribution plot for hospital length of stay and contribution of low or moderate RoB comparisons to each network estimate of hospital length of stay

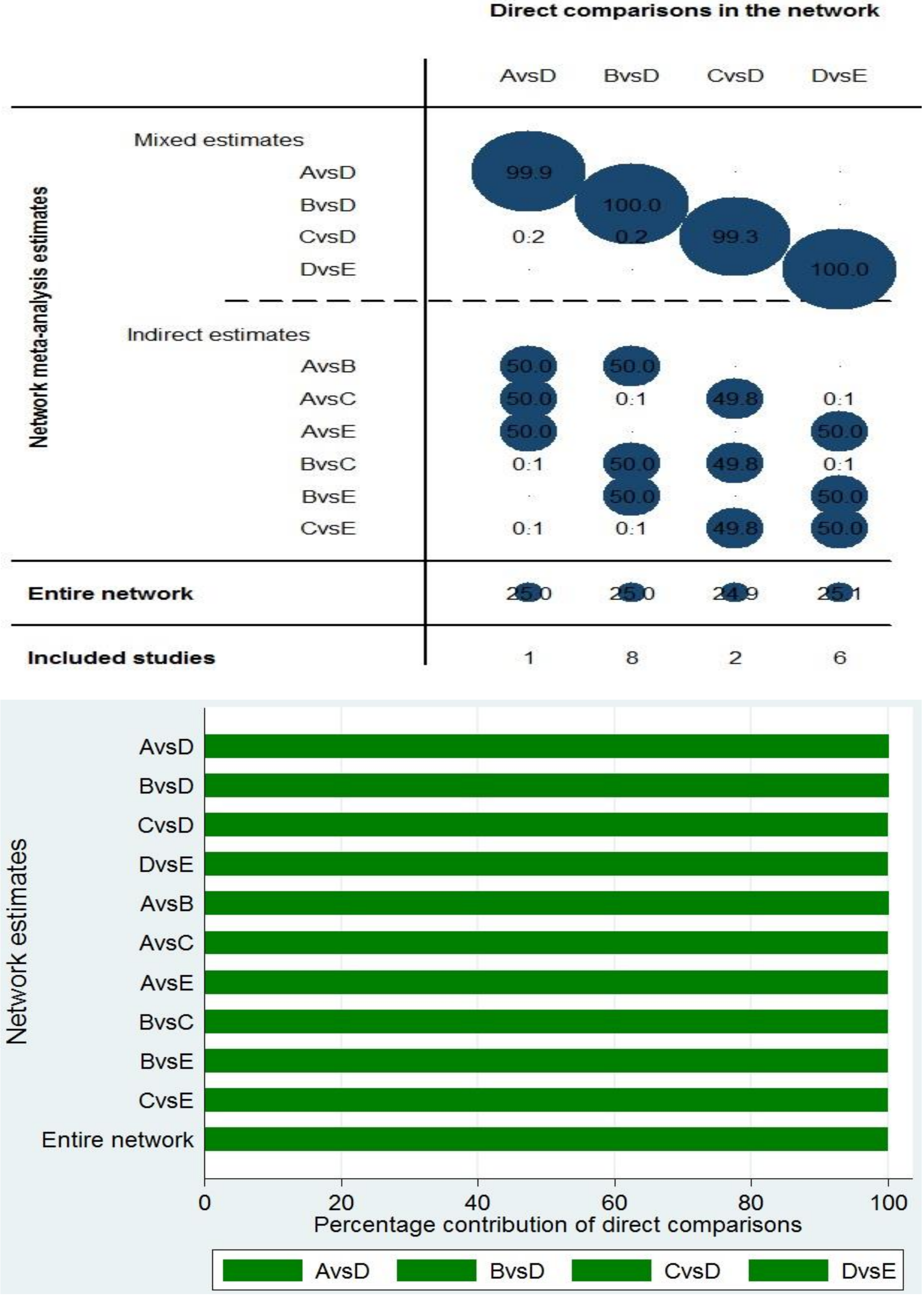

A: Synbiotics; B: Probiotics; C: Probiotics; D: EPN; E: TPN

Table S 8.8 Result of GRADE for hospital length of stay

	Nature of the evidence	Study limitations	Imprecision	Inconsistency	Indirectness	Publication bias	Confidence	Downgrading due to
A vs B	Indirect estimated	No downgrade	Downgrade because point estimate >1.0 but lower limit<0.80	No downgrade	No downgrade	No downgrade	MODERATE	Imprecision
A vs C	Indirect estimated	No downgrade	Downgrade because point estimate < 1.0 but upper limit >1.25	No downgrade	No downgrade	No downgrade	MODERATE	Imprecision
A vs D	Mixed estimated	No downgrade	Downgrade because point estimate < 1.0 but upper limit >1.25	No downgrade	No downgrade	No downgrade	HIGH	--
A vs E	Indirect estimated	No downgrade	Downgrade because point estimate >1.0 but lower limit<0.80	No downgrade	No downgrade	No downgrade	MODERATE	Imprecision
B vs C	Indirect estimated	No downgrade	Downgrade because point estimate < 1.0 but upper limit >1.25	No downgrade	No downgrade	Downgrade	LOW	Imprecision<br>Publication bias
B vs D	Mixed estimated	No downgrade	Downgrade because point estimate < 1.0 but upper limit >1.25	Downgrade because pair heterogeneity I^2=79.5%	No downgrade	Downgrade	VERY LOW	Imprecision<br>Inconsistency<br>Publication bias
B vs E	Indirect estimated	No downgrade	Downgrade because point estimate < 1.0 but upper limit >1.25	No downgrade	No downgrade	No downgrade	MODERATE	Imprecision
C vs D	Mixed estimated	No downgrade	Downgrade because point estimate >1.0 but lower limit<0.80	Downgrade because pair heterogeneity I^2=86.8%	No downgrade	No downgrade	LOW	Imprecision<br>Inconsistency
C vs E	Indirect estimated	No downgrade	Downgrade because point estimate >1.0 but lower limit<0.80	No downgrade	No downgrade	No downgrade	HIGH	--
D vs E	Mixed estimated	No downgrade	Downgrade because point estimate >1.0 but lower limit<0.80	No downgrade	No downgrade	Downgrade	LOW	Imprecision<br>Publication bias
Ranking of treatments		No downgrade	No downgrade	Downgrade because global heterogeneity I^2=74.57%	No downgrade	Downgrade	LOW	Inconsistency<br>Publication bias

A: Synbiotics; B: Probiotics; C: Probiotics; D: EPN; E: TPN

Figure S 8.9 Contribution plot for ICU length of stay and contribution of low or moderate RoB comparisons to each network estimate of ICU length of stay

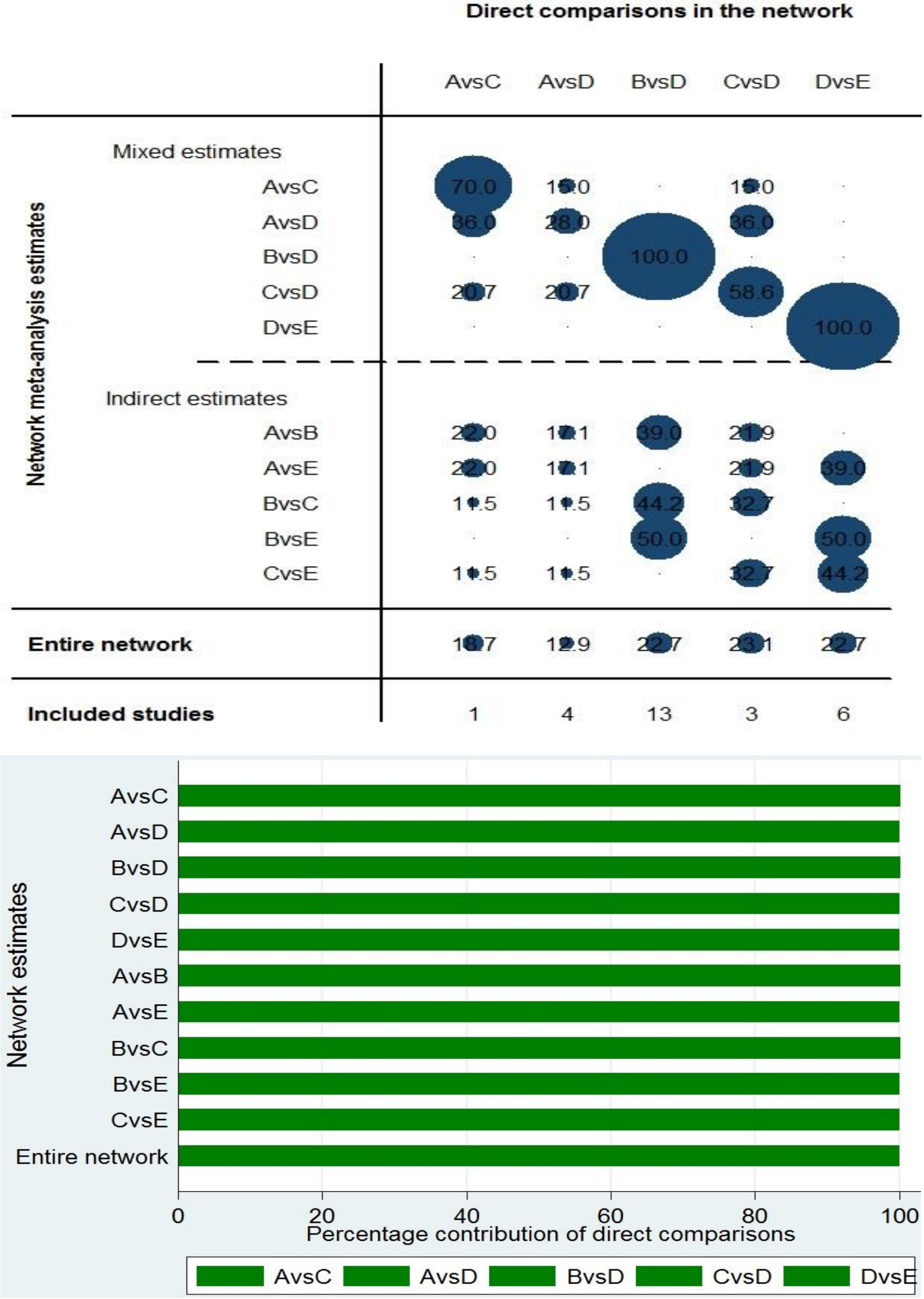

A: Synbiotics; B: Probiotics; C: Probiotics; D: EPN; E: TPN

Table S 8.9 Result of GRADE for ICU length of stay

	Nature of the evidence	Study limitations	Imprecision	Inconsistency	Indirectness	Publication bias	Cinfidence	Downgrading due to
A vs B	Indirect estimated	No downgrade	Downgrade because point estimate < 1.0 but upper limit >1.25	No downgrade	No downgrade	No downgrade	MODERATE	Imprecision
A vs C	Mixed estimated	No downgrade	Downgrade because point estimate >1.0 but lower limit<0.80	No downgrade	No downgrade	No downgrade	MODERATE	Imprecision
A vs D	Mixed estimated	No downgrade	Downgrade because point estimate < 1.0 but upper limit >1.25	Downgrade because pair heterogeneity I^2=70.6%	No downgrade	Downgrade	VERY LOW	Imprecision Inconsistency Publication bias
A vs E	Indirect estimated	No downgrade	Downgrade because point estimate < 1.0 but upper limit >1.25	No downgrade	No downgrade	No downgrade	MODERATE	Imprecision
B vs C	Indirect estimated	No downgrade	Downgrade because point estimate < 1.0 but upper limit >1.25	No downgrade	No downgrade	No downgrade	MODERATE	Imprecision
B vs D	Mixed estimated	No downgrade	Downgrade because point estimate >1.0 but lower limit<0.80	Downgrade because pair heterogeneity I^2=89.1%	No downgrade	Downgrade	VERY LOW	Imprecision Inconsistency Publication bias
B vs E	Indirect estimated	No downgrade	Downgrade because point estimate >1.0 but lower limit<0.80	No downgrade	No downgrade	No downgrade	MODERATE	Imprecision
C vs D	Mixed estimated	No downgrade	Downgrade because point estimate >1.0 but lower limit<0.80	No downgrade	No downgrade	Downgrade	LOW	Imprecision Publication bias
C vs E	Indirect estimated	No downgrade	Downgrade because point estimate >1.0 but lower limit<0.80	No downgrade	No downgrade	No downgrade	MODERATE	Imprecision
D vs E	Mixed estimated	No downgrade	Downgrade because point estimate >1.0 but lower limit<0.80	Downgrade because pair heterogeneity I^2=68.5%	No downgrade	Downgrade	VERY LOW	Imprecision Inconsistency Publication bias
Ranking of treatments		No downgrade	No downgrade	Downgrade because global heterogeneity I^2=83.60%	No downgrade	Downgrade	LOW	Imprecision Publication bias

A: Synbiotics; B: Probiotics; C: Probiotics; D: EPN; E: TPN

Figure S 8.10 Contribution plot for the duration of mechanical ventilation mortality and of low or moderate RoB comparisons to each network estimate of the duration of mechanical ventilation

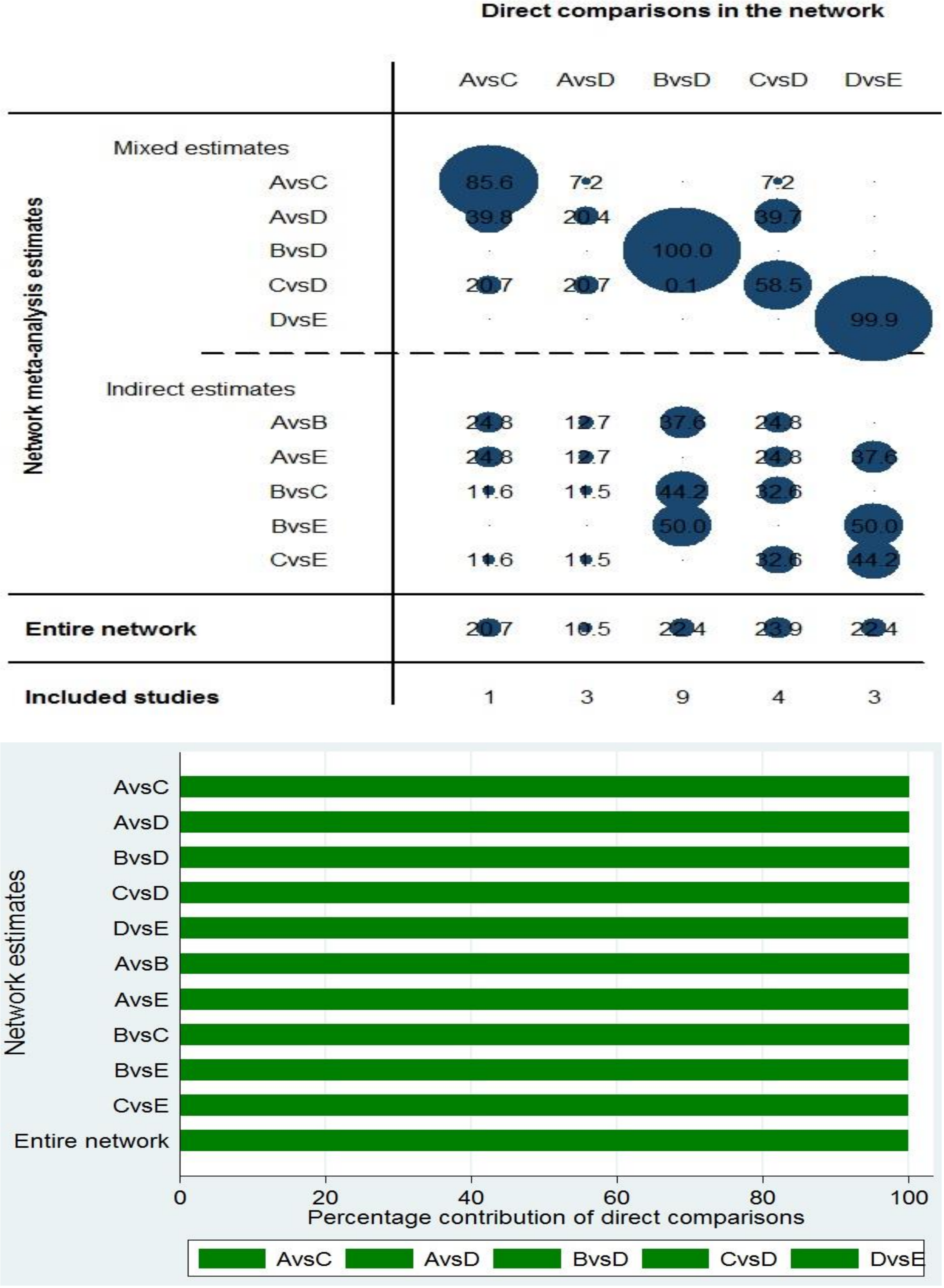

A: Synbiotics; B: Probiotics; C: Probiotics; D: EPN; E: TPN

Table S 8.10 Result of GRADE for the duration of mechanical ventilation

	Nature of the evidence	Study limitations	Imprecision	Inconsistency	Indirectness	Publication bias	Confidence	Downgrading due to
A vs B	Indirect estimated	No downgrade	Downgrade because point estimate < 1.0 but upper limit >1.25	No downgrade	No downgrade	No downgrade	MODERATE	Imprecision
A vs C	Mixed estimated	No downgrade	Downgrade because point estimate >1.0 but lower limit<0.80	No downgrade	No downgrade	No downgrade	MODERATE	Imprecision
A vs D	Mixed estimated	No downgrade	Downgrade because point estimate < 1.0 but upper limit >1.25	Downgrade because pair heterogeneity I^2=89.3%	No downgrade	Downgrade	VERY LOW	Imprecision Inconsistency Publication bias
A vs E	Indirect estimated	No downgrade	No downgrade	No downgrade	No downgrade	No downgrade	HIGH	--
B vs C	Indirect estimated	No downgrade	Downgrade because point estimate < 1.0 but upper limit >1.25	No downgrade	No downgrade	No downgrade	MODERATE	Imprecision
B vs D	Mixed estimated	No downgrade	Downgrade because point estimate < 1.0 but upper limit >1.25	Downgrade because pair heterogeneity I^2=93.6%	No downgrade	Downgrade	VERY LOW	Imprecision Inconsistency Publication bias
B vs E	Indirect estimated	No downgrade	Downgrade because point estimate < 1.0 but upper limit >1.25	No downgrade	No downgrade	No downgrade	MODERATE	Imprecision
C vs D	Mixed estimated	No downgrade	Downgrade because point estimate >1.0 but lower limit<0.80	Downgrade because pair heterogeneity I^2=83.2%	No downgrade	Downgrade	VERY LOW	Imprecision Inconsistency Publication bias
C vs E	Indirect estimated	No downgrade	Downgrade because point estimate < 1.0 but upper limit >1.25	No downgrade	No downgrade	No downgrade	MODERATE	Imprecision
D vs E	Mixed estimated	No downgrade	Downgrade because point estimate < 1.0 but upper limit >1.25	Downgrade because pair heterogeneity I^2=66.7%	No downgrade	Downgrade	LOW	Imprecision Inconsistency
Ranking of treatments		No downgrade	No downgrade	Downgrade because global heterogeneity I^2=90.55%	No downgrade	Downgrade	LOW	Inconsistency Publication bias

A: Synbiotics; B: Probiotics; C: Probiotics; D: EPN; E: TPN
