# Supplementary material for: Probiotic Supplementation Prevents the Development of Ventilator-Associated Pneumonia for Mechanically Ventilated ICU Patients: A Systematic Review and Network Meta-analysis of Randomized Controlled Trials
Source: Front Nutr. 2022 Jul 8;9:919156. doi: 10.3389/fnut.2022.919156 (PMC9307490; doi:10.3389/fnut.2022.919156)
Supplement: Supplementary File 9 — Treatment ranking and SUCRA ranking curve for each outcome.pdf. [file Data_Sheet_9.PDF]

Supplementary file 9
Treatment ranking and surface under the cumulative ranking curves (SUCRA) for each outcome

Figure S 9.1 Treatment ranking and SUCRA ranking curve for nosocomial infection

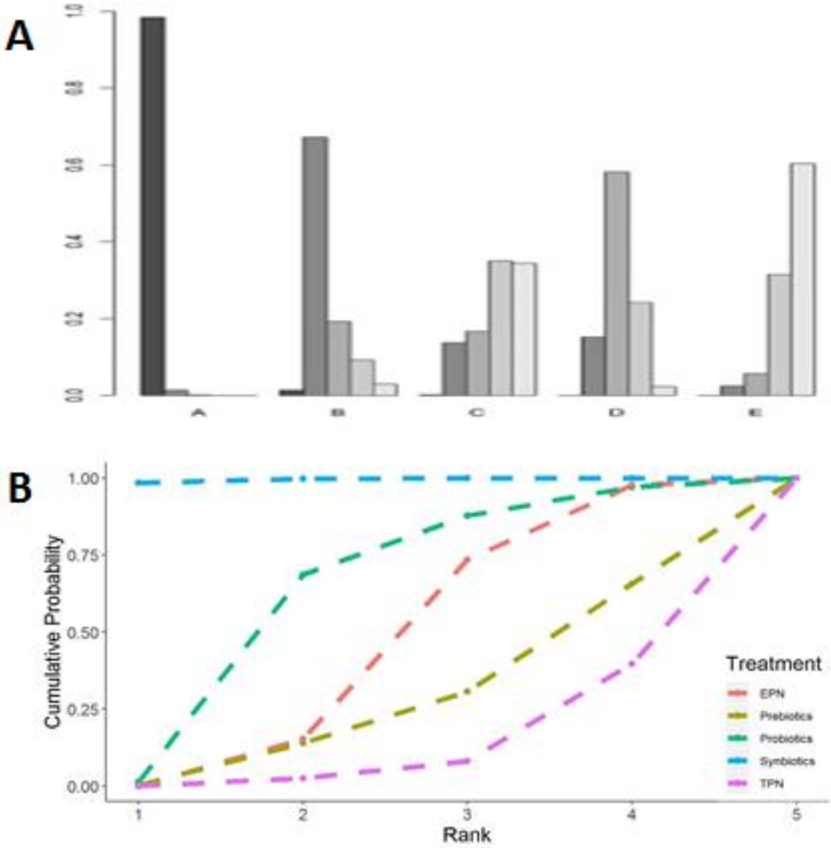

A: Synbiotics; B: Probiotics; C: Probiotics; D: EPN; E: TPN

Figure S 9.2 Treatment ranking and SUCRA ranking curve for bloodstream infection

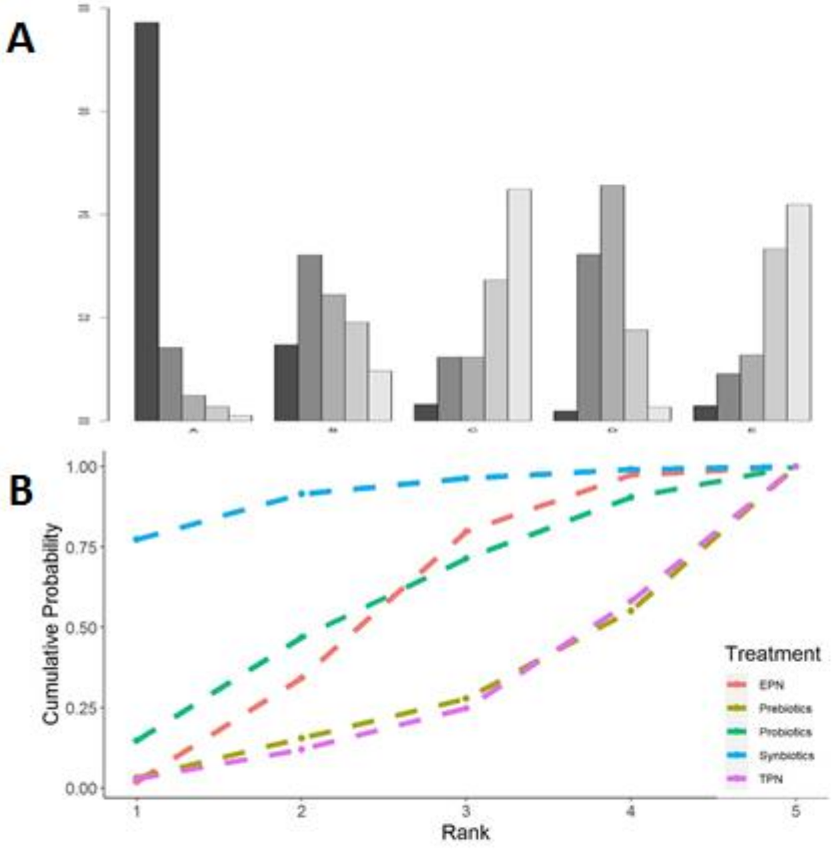

A: Synbiotics; B: Probiotics; C: Probiotics; D: EPN; E: TPN

Figure S 9.3 Treatment ranking and SUCRA ranking curve for urinary tract infection

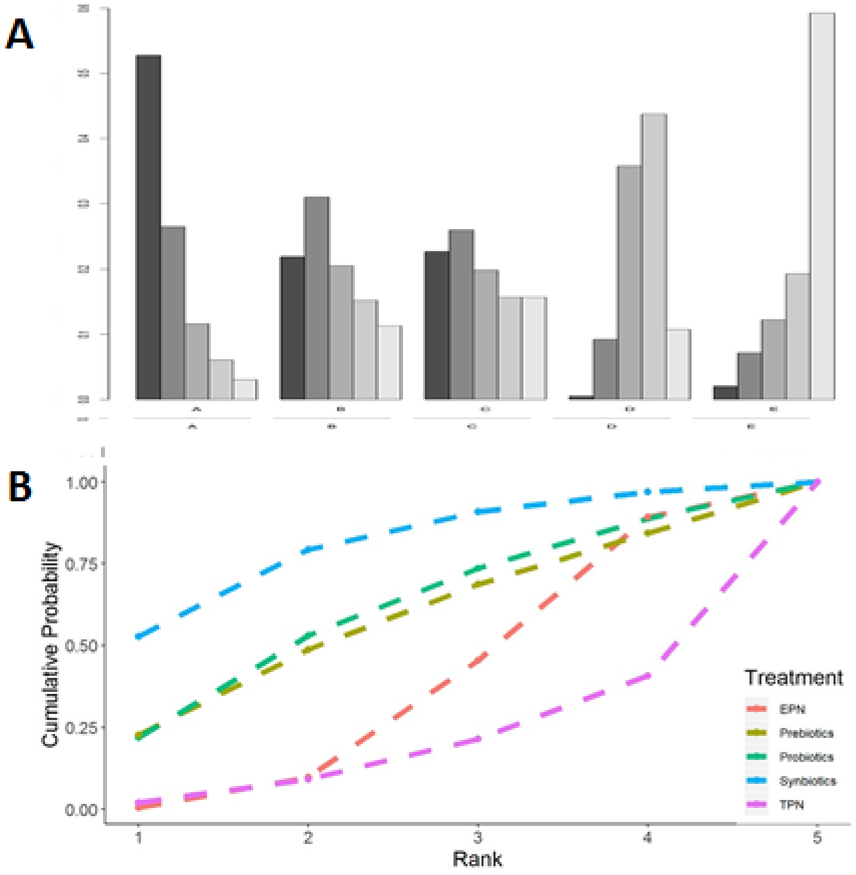

A: Synbiotics; B: Probiotics; C: Probiotics; D: EPN; E: TPN

Figure S 9.4 Treatment ranking and SUCRA ranking curve for diarrhea

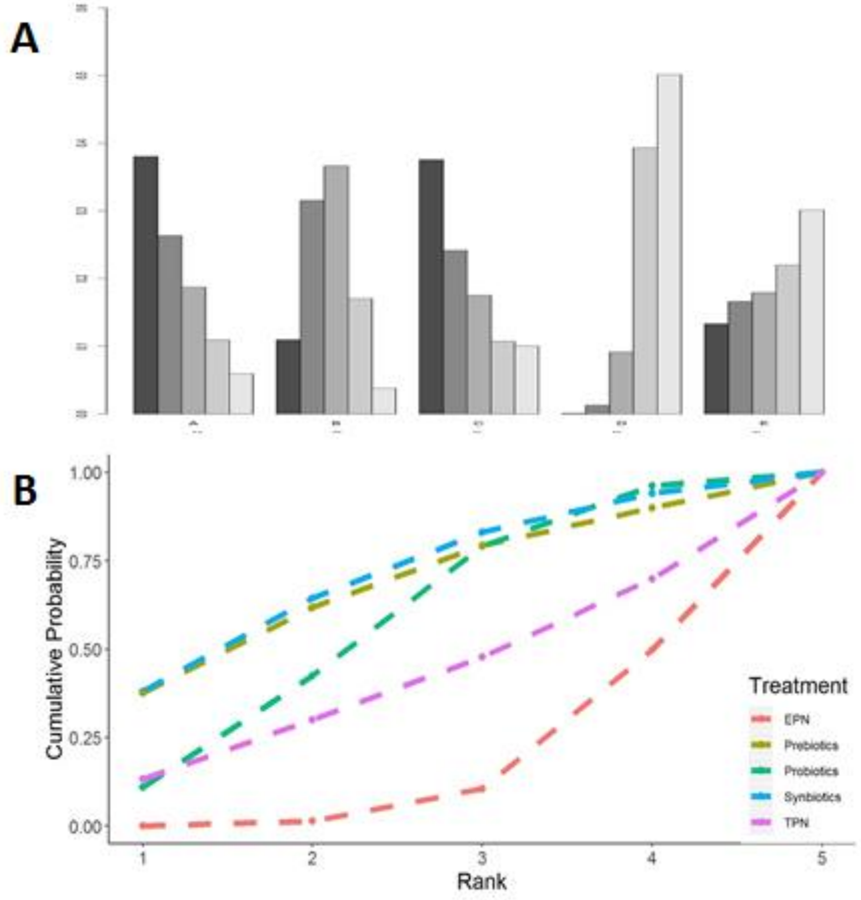

A: Synbiotics; B: Probiotics; C: Probiotics; D: EPN; E: TPN

Figure S 9.5 Treatment ranking and SUCRA ranking curve for hospital mortality

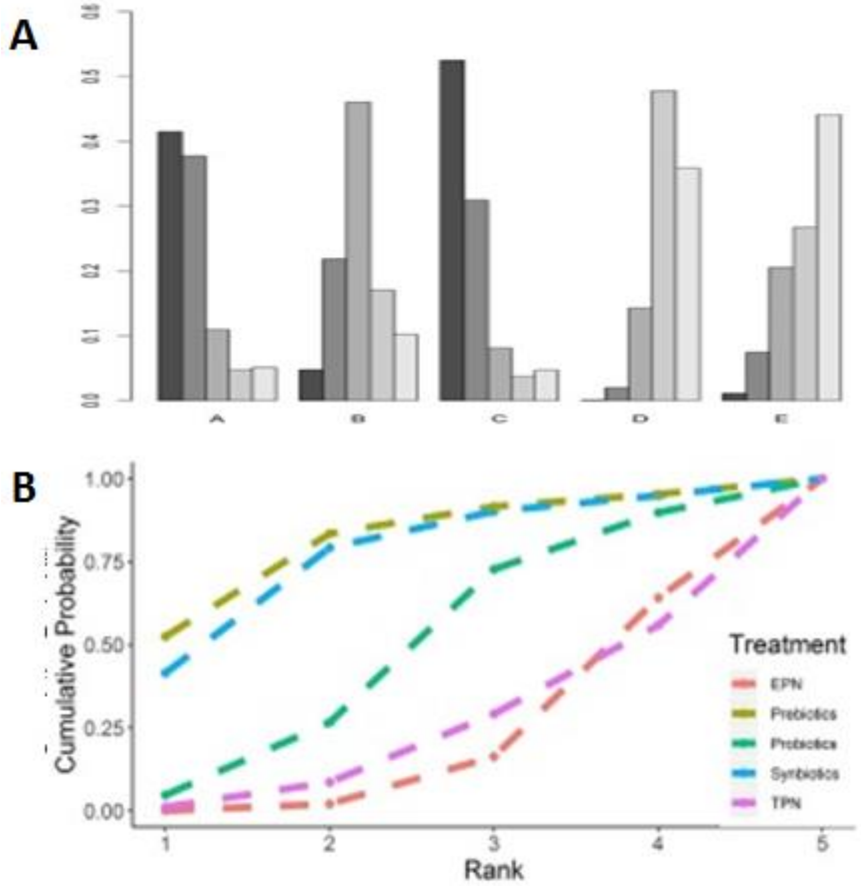

A: Synbiotics; B: Probiotics; C: Probiotics; D: EPN; E: TPN

Figure S 9.6 Treatment ranking and SUCRA ranking curve for ICU mortality

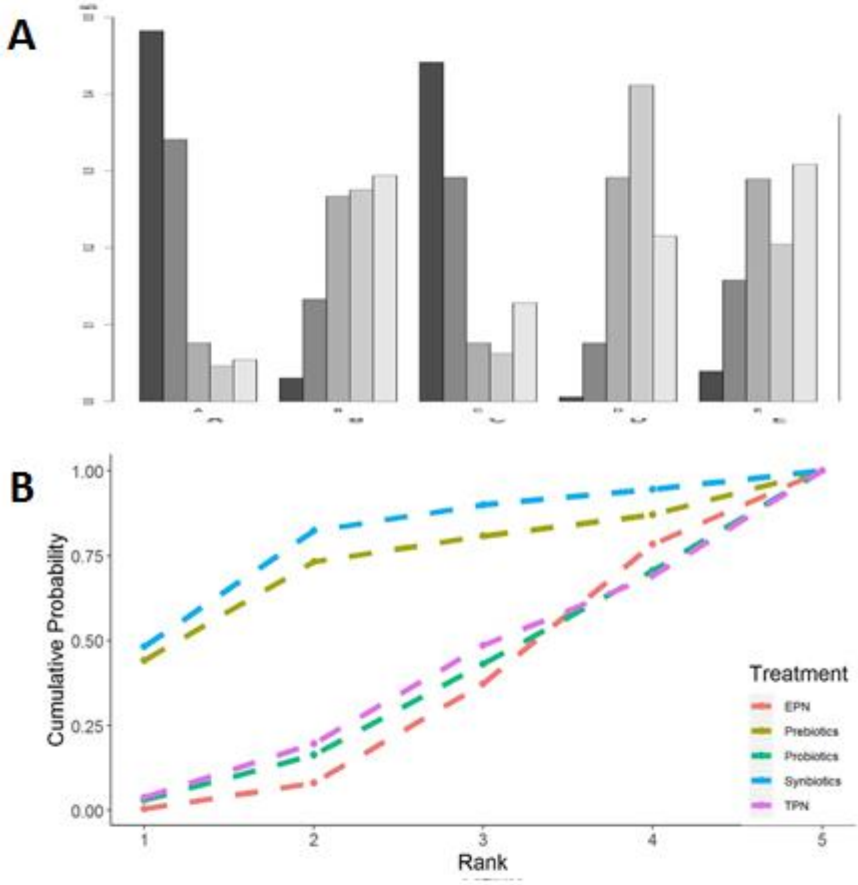

A: Synbiotics; B: Probiotics; C: Probiotics; D: EPN; E: TPN

Figure S 9.7 Treatment ranking and SUCRA ranking curve for hospital lengtn of stay

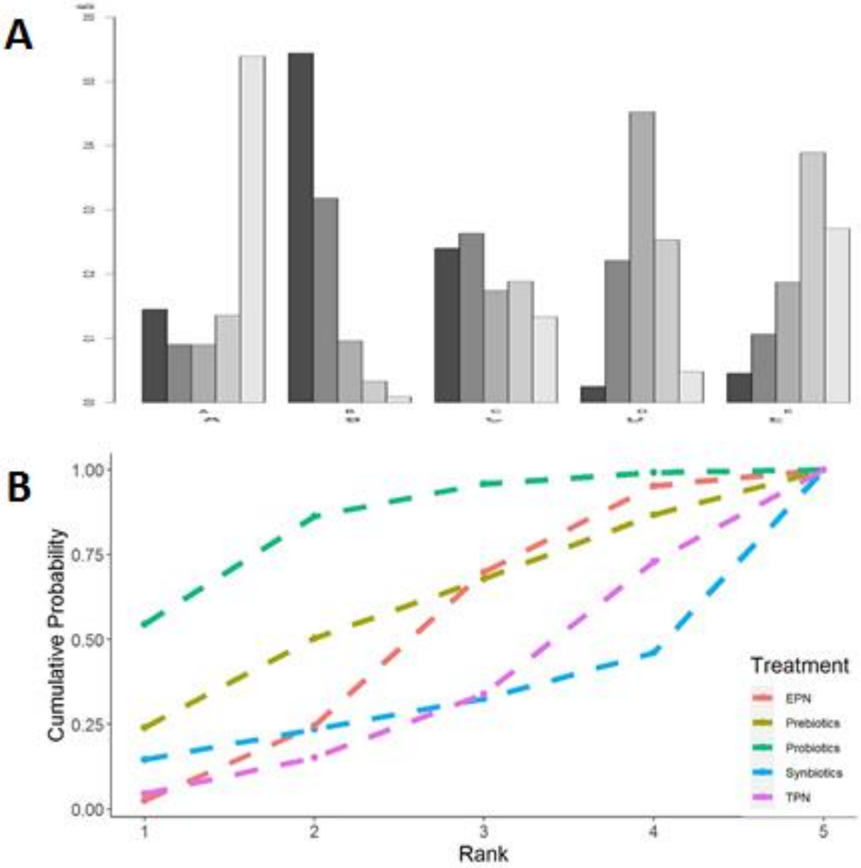

A: Synbiotics; B: Probiotics; C: Probiotics; D: EPN; E: TPN

Figure S 9.8 Treatment ranking and SUCRA ranking curve for ICU length of stay

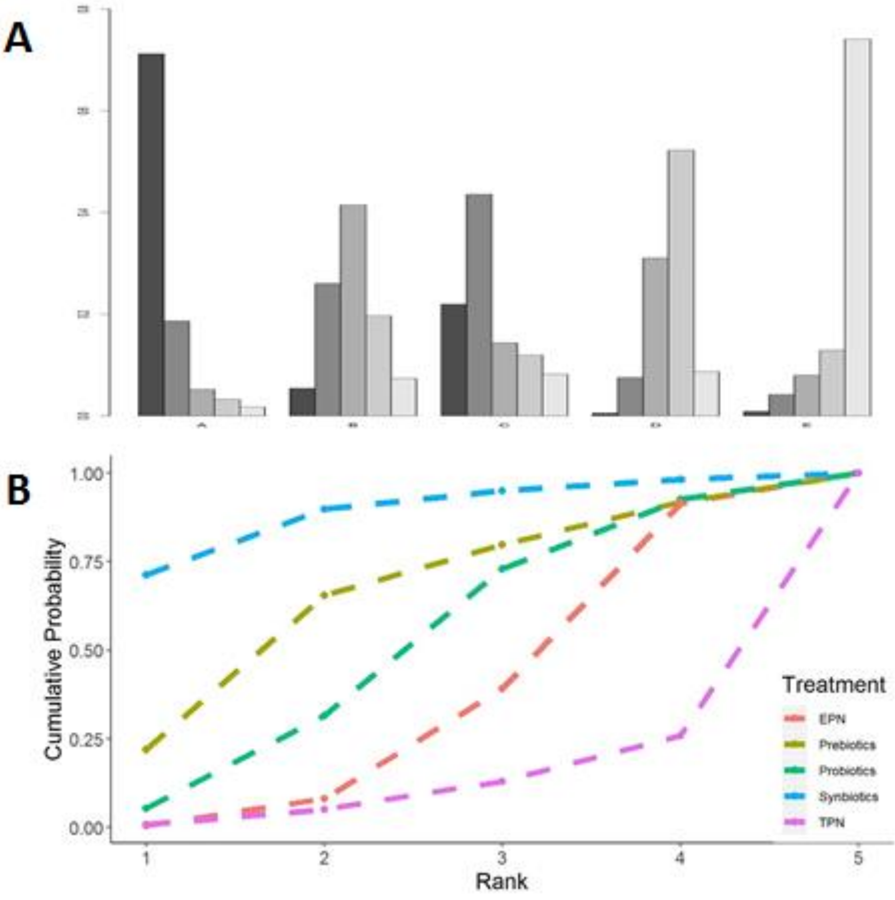

A: Synbiotics; B: Probiotics; C: Probiotics; D: EPN; E: TPN

Figure S 9.9 Treatment ranking and SUCRA ranking curve for the duration of mechanical ventilation

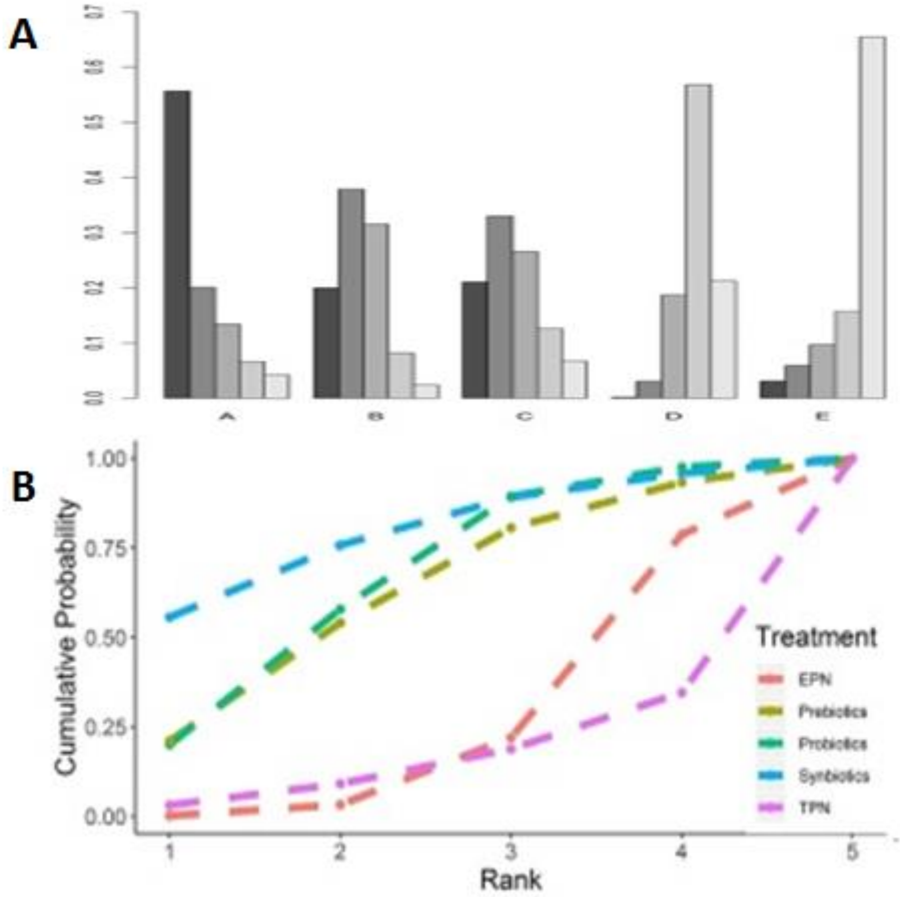

A: Synbiotics; B: Probiotics; C: Probiotics; D: EPN; E: TPN
