# Supplementary material for: Probiotic Supplementation Prevents the Development of Ventilator-Associated Pneumonia for Mechanically Ventilated ICU Patients: A Systematic Review and Network Meta-analysis of Randomized Controlled Trials
Source: Front Nutr. 2022 Jul 8;9:919156. doi: 10.3389/fnut.2022.919156 (PMC9307490; doi:10.3389/fnut.2022.919156)
Supplement: Supplementary File 10 — Sensitivity analyses for the risk of primary outcome.pdf. [file Data_Sheet_10.PDF]

Supplementary file 10
Sensitivity analyses

Table S 10 Sensitivity analyses for the risk of ventilation-associated pneumonia (OR with 95% CrI and rank order)

	Synbiotics	Probiotics	Prebiotics	EPN	TPN	Number of studies	Partcipants
**Overall patients**	0.66 (0.37, 1.15)<br>Rank 1	**0.75 (0.58, 0.95)**<br>**Rank 2**	1.14 (0.63, 1.98)<br>Rank 5	reference<br>Rank 3	1.01 (0.67, 1.54)<br>Rank 4	25	7721
**Studies with low-moderate ROB**	0.66 (0.38, 1.12)<br>Rank 1	**0.76 (0.57, 0.98)**<br>**Rank 2**	1.69 (0.81, 3.45)<br>Rank 5	Reference<br>Rank 3	0.98 (0.58, 1.67)<br>Rank 4	20	7439
**Studies with robust diagnostic criteria for VAP**	0.66 (0.37, 1.14)<br>Rank 1	**0.76 (0.58, 0.99)**<br>**Rank 2**	1.14 (0.61, 2.00)<br>Rank 5	reference<br>Rank 3	1.01 (0.66, 1.56)<br>Rank 4	23	7596
**Multicentric studies**	0.89 (0.22, 3.59)<br>Rank 2	0.70 (0.34, 1.18)<br>Rank 1	2.63 (0,79, 8.82)<br>Rank 5	Reference<br>Rank 3	1.12 (0.49, 2.75)<br>Rank 4	8	5920
**Single centre studies**	0.36 (0.11, 1.21)<br>Rank 1	0.59 (0.31, 1.00)<br>Rank 2	0.81 (0.27, 2.28)<br>Rank 3	Reference<br>Rank 4	1.05 (0.37, 3.00)<br>Rank 5	17	1801

OR:odds ratio. CrI:credible interval. EPN: enteral nutrition and/or adjuvant peripheral parenteral nutrition. ROB: Risk of bia; TPN: total parenteral nutrition.VAP: ventilation-associated pneumonia.
